# Synthesis of Quinoline and Dihydroquinoline Embelin Derivatives as Cardioprotective Agents

**DOI:** 10.1021/acs.jnatprod.2c00924

**Published:** 2023-02-07

**Authors:** Pedro Martín-Acosta, Irene Cuadrado, Laura González-Cofrade, Roberto Pestano, Sonsoles Hortelano, Beatriz de las Heras, Ana Estévez-Braun

**Affiliations:** † Instituto Universitario de Bio-Orgánica Antonio González, Departamento de Química Orgánica, Universidad de La Laguna, Avenida Astrofísico Francisco Sánchez N° 2, 38206, La Laguna, Tenerife, Spain; ‡ Departamento de Farmacología, Farmacognosia y Botánica, Facultad de Farmacia, Universidad Complutense de Madrid (UCM), Plaza Ramón y Cajal s/n, 28040, Madrid, Spain; § Unidad de Terapias Farmacológicas, Área de Genética Humana, Instituto de Investigación de Enfermedades Raras (IIER), Instituto de Salud Carlos III, Carretera de Majadahonda-Pozuelo Km 2, 28220, Madrid, Spain

## Abstract

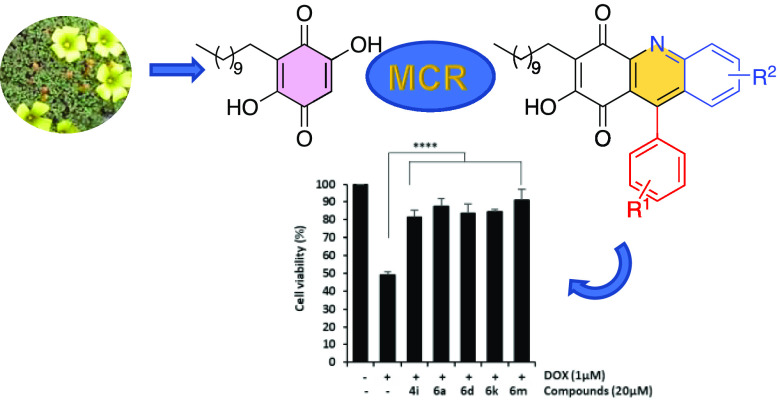

A set of new dihydroquinoline embelin derivatives was obtained from the reaction of the natural benzoquinone embelin (**1**) with anilines and aromatic aldehydes in the presence of AgOTf. The synthesis of these compounds involves the formation of a Knoevenagel adduct, followed by nucleophilic addition of aniline and subsequent electrocyclic ring closure. The scope of the reaction regarding the aldehydes and anilines was determined. Quinoline derivatives were also obtained from the corresponding dihydroquinolines under oxidation with DDQ. The cardioprotective activity of the synthesized compounds was screened using a doxorubicin-induced cardiotoxicity model in H9c2 cardiomyocytes. Some structure–activity relationships were outlined, and the best activities were achieved with quinoline-embelin derivatives having a 4-nitrophenyl group attached at the pyridine ring. The obtained results indicated that embelin derivatives **4i**, **6a**, **6d**, **6k**, and **6m** could have potential as cardioprotective agents, as they attenuated a DOX-induced cardiotoxicity effect acting on oxidative stress and apoptosis.

Quinolines and dihydroquinolines are important biologically active nitrogen-containing heterocycles found widespread in nature.^1^ These molecules present a wide range of applications, particularly in industry as sensors,^2^ agrochemicals,^3^ or luminescent materials,^4^ and in medicinal chemistry, where they play an important role in drug discovery. Many molecules that contain quinoline structures as a core structure or fused to other bioactive relevant scaffolds exhibit antimalarial,^5,6^ antibacterial,^7^ anticancer,^8^ anti-inflammatory,^9^ and antioxidant activity.^10^ Some representative examples are the antimalarial quinine,^11^ megistoquinones I and II with antibacterial activity,^12^ ciprofloxacin used for the treatment of respiratory and urinary infections,^13^ and lenvatinib, a tyrosine kinase inhibitor used as an effective antitumor agent.^14^ Another example of special interest is 4-azapodophyllotoxin, a potent inhibitor of tubulin polymerization,^15^ synthesized to overcome some therapeutic limitations of podophyllotoxins such as water solubility, metabolic inactivation, or toxicity (Figure 1).^16−18^

**Figure 1:**
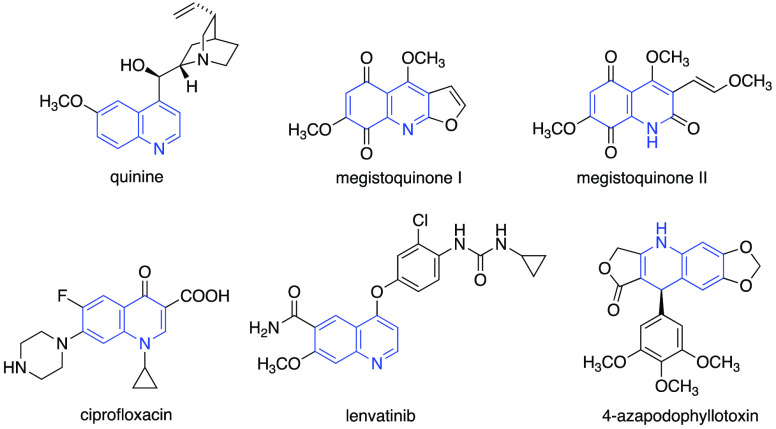
Representative bioactive compounds with quinoline and dihydroquinoline structures.

Quinolines can be obtained through diverse methodologies such as the Skraup,^19^ Doebner–von Miller,^20^ Friedleander,^21^ Gould–Jacobs,^22^ Pfitzinger,^23^ and Combes syntheses.^24^ However, efficient access to compounds that have quinoline-type structures fused to other biologically relevant scaffolds continues to represent a challenge from an academic and industrial perspective. In this sense, the three-component reaction of aromatic amines, aldehydes, and 1,3-dicarbonyl compounds represents an efficient method for the synthesis of these potentially bioactive heterocycles.^25^

Only a few methodologies are reported to access molecules with a quinone core fused to quinoline or dihydroquinoline heterocycles, all of which are limited to 2-hydroxy-1,4-naphthoquinone as starting material (Scheme 1). Shunjun et al. reported the first synthesis of this type of compounds fused to *p*-naphthoquinone fragments employing ionic liquids,^26^ while Singh et al. reported the first synthesis of quinoline derivatives incorporating an *o*-naphthoquinone moiety.^27^ Aiming to obtain 4-azapodophyllotoxin analogs, Wu’s group optimized a synthesis by condensation of 3,4-methylenedioxyaniline, aldehydes, and 2-hydroxy-1,4-naphthoquinone in the presence of l-proline.^28^ Pélinski et al. synthesized this type of compounds from 2-aminobenzyl alcohols and 2-hydroxy-1,4-naphthoquinone.^29^

**Scheme 1:**
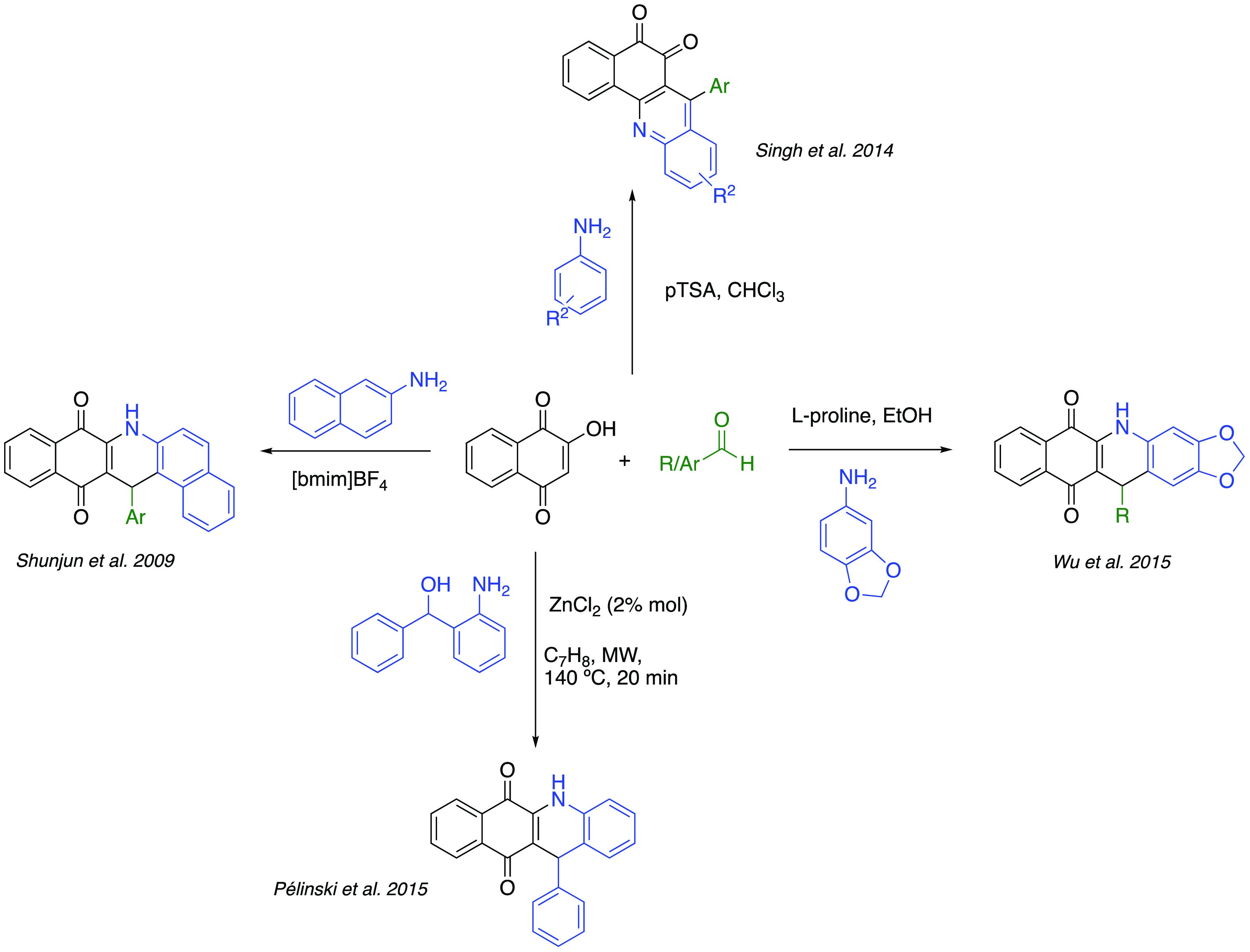
Synthetic Approaches toward Quinoline and Dihydroquinoline Fused to Naphthoquinones

Embelin (**1**) is a natural benzoquinone isolated from *Oxalis erythrorhiza*.^30^ This compound and some derivatives have shown activity against multiple targets such as CK2,^31,32^ XIAP,^33^ α-glucosidase,^34^ B-RAF,^35^ or PKC.^36^ It acts as an inhibitor of neuroserpin polymerization^37^ and as a GPR84 agonist,^38^ modulates Akt/β-catenin^39^ and p38 MAPK,^40^ and blocks the NF-κβ signaling pathway.^41,42^ This plethora of protein targets and pathway modulations have attracted the interest of medicinal chemists.^43−45^

Our research group improved selectivity and potency of this natural product by increasing its complexity and structural diversification, using a domino Knoevenagel–Michael addition–intramolecular cyclization sequence developed by us.^46,47^ Aiming to extend the chemical diversity around nitrogen-containing heterocycles fused to this natural benzoquinone, we decided to carry out the synthesis of embelin derivatives fused to dihydroquinoline and quinoline structures.

Previous studies have reported the protective effects of embelin against myocardial injury.^48−50^ However, embelin has not been tested so far for its cardioprotective effects against anthracycline-induced toxicity. In this work we investigated whether cardiotoxicity induced by doxorubicin could be attenuated by embelin derivatives.

## Results and Discussion

### Chemistry

Initially, we carried out the reaction from embelin (**1**), 4-nitrobenzaldehyde (**2a**), and aniline (**3a**) in CHCl_3_, under reflux with pTSA (20 mol %) as a catalyst, obtaining product **4a** in 39% yield (Table 1, entry 1). Next, we tried the use of microwave irradiation to improve the yields and reduce the reaction time. When the reaction mixture was irradiated at 120 °C for 1 h, the dihydropyridine adduct (**4a**) was obtained (41%) together with arylaminoembelin (**5a**) (14%), resulting in the direct nucleophilic attack of aniline (entry 2). Additionally, other reaction conditions were explored. Thus, an excess of aldehyde and aniline (1.5 equiv) led to an improvement in the yield of the process, obtaining **4a** in a 63% yield together with **5a** (18%) (entry 3). Other types of catalysts were used such as EDDA, Et_3_N, TFA, and Lewis acids (InCl_3_, FeCl_3_, BiCl_3_, AgOTf). Different solvents, reaction times, and temperatures were also evaluated. The results obtained are shown in Table 1.

**Table 1:**
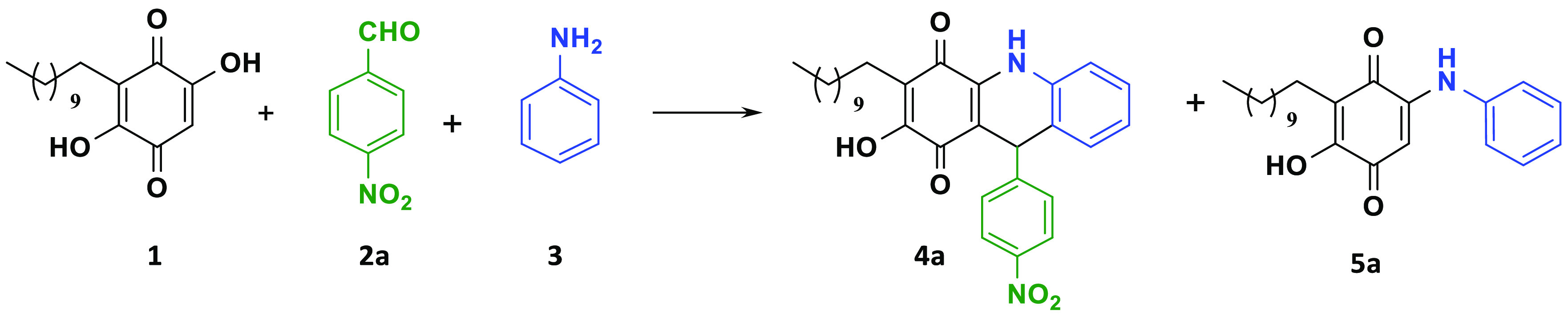
Improvement of Reaction Conditions with Embelin (**1**), 4-Nitrobenzaldehyde (**2**), and Aniline (**3**)

					Yield (%)	
Entry	**1**/**2a**/**3**	Catalyst	Reaction conditions	Time	**4a**	**5a**
1	1.0/1.0/1.0	20% pTSA	CHCl_3_, reflux	48 h	39	
2	1.0/1.0/1.0	20% pTSA	CHCl_3_, MW, 120 °C	1 h	41	14
3	1.0/1.5/1.5	20% pTSA	CHCl_3_, MW, 120 °C	1 h	63	18
4	1.0/1.5/1.5	20% pTSA	CHCl_3_, MW, 150 °C	30 min	49	25
5	1.0/1.5/1.5	20% pTSA	DCE, MW, 120 °C	1 h	33	18
6	1.0/1.5/1.5	20% pTSA	EtOH, MW, 120 °C	30 min	56	31
7	1.0/1.5/1.5	20% pTSA	EtOH, MW, 150 °C	20 min	66	20
8	1.0/1.5/1.5	20% TFA	EtOH, MW, 150 °C	15 min	43	27
9	1.0/1.5/1.5	20% EDDA	DCE, MW, 120 °C	15 min		
10	1.0/1.5/1.5	20% Et_3_N	EtOH, MW, 150 °C	15 min	28	
11	1.0/1.5/1.5	20% InCl_3_	EtOH, MW, 150 °C	15 min	57	24
12	1.0/1.5/1.5	20% FeCl_3_	EtOH, MW, 150 °C	15 min	31	14
13	1.0/1.5/1.5	20% BiCl_3_	EtOH, MW, 150 °C	15 min	43	21
14	1.0/1.5/1.5	20% AgOTf	EtOH, MW, 150 °C	15 min	80	13
15	1.0/1.5/1.5	10% AgOTf	EtOH, MW, 150 °C	15 min	64	11
16	1.0/1.5/1.5	20% AgOTf	H_2_O, MW, 150 °C	15 min	46	6

Use of polar and more environmentally friendly solvents, such as EtOH instead of CHCl_3_, as well as higher reaction temperatures (150 °C, entry 7), afforded product **4a** in a 66% yield in 20 min. Different Lewis acids such as InCl_3_, FeCl_3_, BiCl_3_, and AgOTf were evaluated, obtaining the best yields with AgOTf (20% mol) as catalyst in EtOH after 15 min of reaction at 150 °C (entry 14). Under these reaction conditions, **4a** was obtained in 80% yield together with **5a** (13%). At this point, and since the arylamino embelin sideproduct could be of interest for their biological potential, the reaction conditions shown in entry 14 were selected.

The improved reaction conditions were used to evaluate the scope of the reaction with respect to the aldehydes and aromatic amines. The use of substituted aromatic aldehydes with electron-withdrawing groups such 4-nitro (**4a**, 80%) or 4-methyl ester (**4g**, 81%) led to the best yields. Moderate to good yields (62–74%) were obtained when introducing halogens in the aromatic ring (**4c**–**4f**). Good results were also obtained with the use of heteroaromatic aldehydes such as 3-thiophenecarboxaldehyde (**4j**, 83%). On the contrary, the introduction of electron-donating groups like 3,4-dimethyl or dimethoxy substituents (**4i** and **4j**) decreased the yields (52% and 56%, respectively). The formation of the corresponding products was not observed when aliphatic aldehydes were used.

We also evaluated the scope with different substituted aromatic and heteroaromatic amines, finding, as expected, a behavior opposite to that observed with the aldehydes. The use of anilines substituted with electron-donating groups such as 4-methoxyaniline (**3b**) (entry 11) and 3,5-dimethylaniline (**3c**) (entry 12) led to the corresponding dihydropyridines **4k** and **4l** in 80% and 92% yield, respectively. A moderate yield (67%) was obtained with 4-bromoaniline (**3d**) (entry 13), while the use of aromatic anilines substituted with an electron-withdrawing group (3-nitroaniline) (**3e**) did not afford the corresponding dihydropyridin derivative, and only the 3-nitrophenylembeline derivative (**5d**) was quantitatively formed.

**Table 2:**
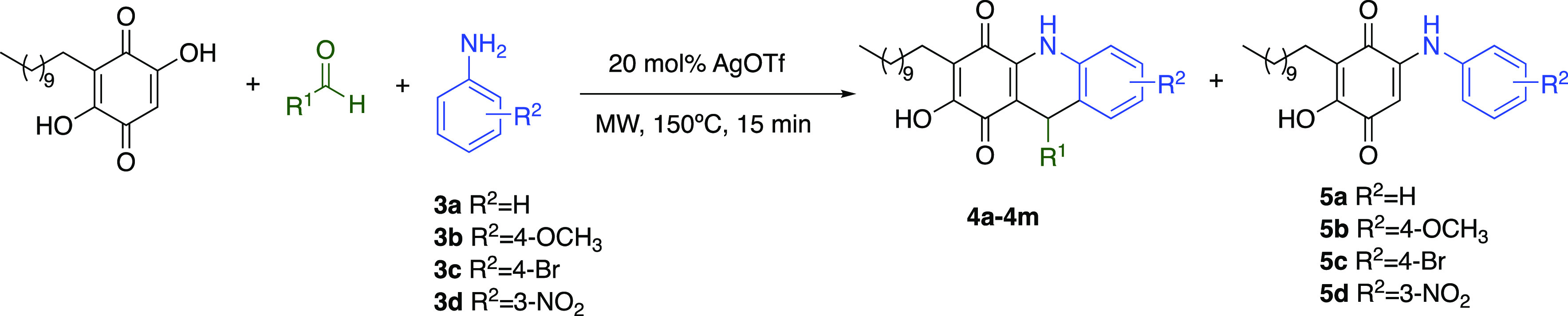
Scope of the Aldehydes and Anilines

Entry	R^1^	R^2^	Yield (%)
1	4-NO_2_-Ph	H	**4a** (80%) + **5a** (13%)
2	Ph	H	**4b** (70%) + **5a** (17%)
3	4-Cl-Ph	H	**4c** (74%) + **5a** (11%)
4	4-Br-Ph	H	**4d** (62%) + **5a** (15%)
5	4-F-Ph	H	**4e** (72%) + **5a** (10%)
6	3-F-Ph	H	**4f** (72%) + **5a** (12%)
7	4-COOCH_3_	H	**4g** (81%) + **5a** (14%)
8	3-thienyl	H	**4h** (83%) + **5a** (10%)
9	3,4-(CH_3_)_2_-Ph	H	**4i** (52%) + **5a** (21%)
10	3,4-(OCH_3_)_2_-Ph	H	**4j** (56%) + **5a** (25%)
11	4-NO_2_-Ph	4-OCH_3_	**4k** (80%) + **5b** (14%)
12	4-NO_2_-Ph	3,5-(CH_3_)_2_	**4l** (92%)
13	4-NO_2_-Ph	4-Br	**4m** (67%) + **5c** (29%)
14	4-NO_2_-Ph	3-NO_2_	**4n** (0%) + **5d** (100%)

Regarding the reaction mechanism, different mechanistic proposals are found in the literature for similar types of transformations. Some of them involve either the direct nucleophilic attack of the carbon next to the amino group^51−53^ or the nucleophilic attack of the amino group to a previously generated Michael adduct and subsequent electrocyclic ring closure.^27,54^ Recently, Sun et al. demonstrated the formation of dimeric species between the aldehyde and the corresponding 1,3-dicarbonyl compounds as reaction intermediates.^55^ This dimers evolve through the nucleophilic attack of the aromatic amine, subsequent hydrolysis, and electrocyclic ring closure.^55^

**Figure 2:**
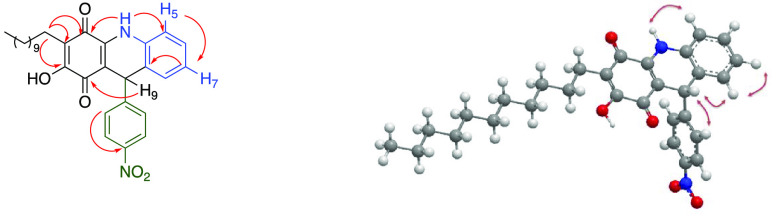
Key HMBC and ROESY correlations for compound **4a**.

The structure of product **4a** was corroborated by the key correlations observed in the HMBC spectrum and the NOE effects observed in selective NOE experiments (Figure 2).

Quinoline derivatives were obtained from the dehydrogenation of the corresponding dihydroquinoline derivatives. A wide range of oxidizing reagents was used (MnO_2_, NBS, Br_2_, *p*-chloroanil, and DDQ), of which DDQ turned out to be the most suitable not only for the short reaction times but also for the easy isolation of the corresponding reaction products. All the dihydroquinoline derivatives previously synthesized (**4a**–**4m**) were treated with DDQ, affording the corresponding quinoline derivatives (**6a**–**6m**) in moderate to good yields (55–88%). Results are depicted in Table 3.

**Table 3:**
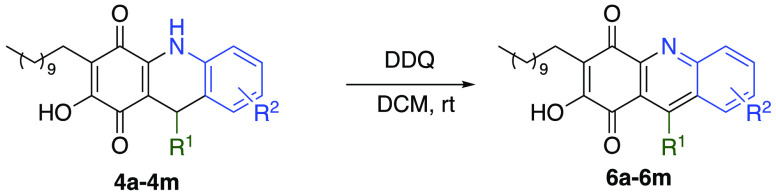

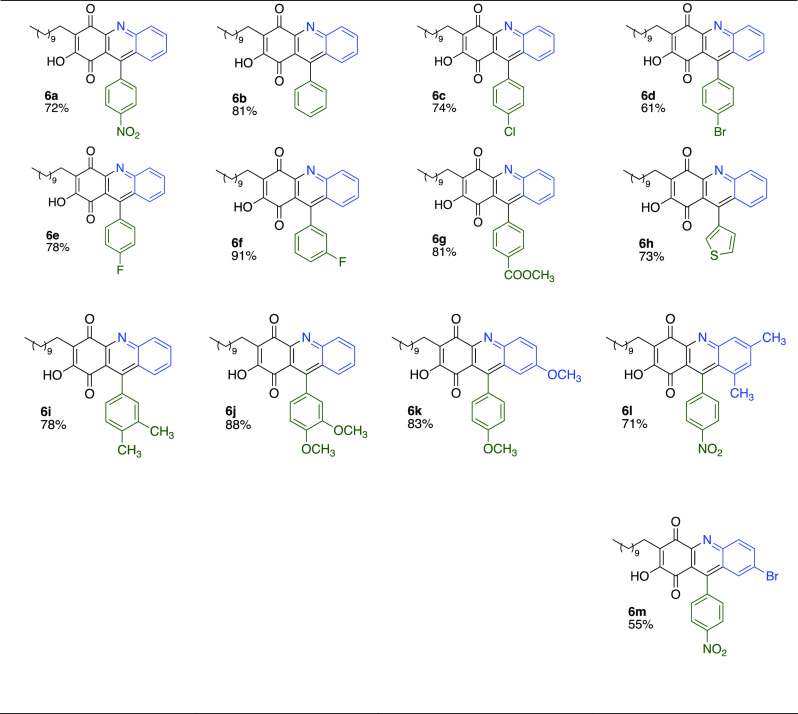
Synthesis of Pyridine Embelin Derivatives

### Cardioprotective Effects

Doxorubicin, which possesses an anthracycline core, is frequently used to treat various types of cancers, including hematological neoplasia and solid malignancies. However, its cardiac side effects, leading to cardiomyopathy and congestive heart failure, have limited the clinical use of this potent anticancer drug.^56^ Therefore, more effective therapeutic agents are needed considering previous reports of embelin against myocardial injury,^48−50^ and since so far protective effects of embelin and embelin derivatives against anthracycline-induced cardiotoxicity have not been tested,^57^ in the present study, we aimed to determine whether cardiotoxicity induced by chemotherapy could be attenuated by embelin derivatives when administrated in conjunction with chemotherapeutical agents.

The potential cytotoxicity of embelin (**1**) together with derivatives (**4a**–**4m**, **5a**–**5d**, **6a**–**6m**) was tested in cardiomyocytes and macrophages in order to ensure that compounds lack toxicity also in immune cells. Cell viability was determined by the MTT assay.^58^ Most of the compounds exhibited cytotoxicity when incubated with cells for 24 h, as shown in Table 4. Four dihydroquinoline derivatives (**4b**, **4f**, **4i**, and **4m**) and six quinoline compounds (**6a**, **6d**, **6e**, **6i**, **6k**, and **6m**) maintained higher viable cell rates in both types of cells. IC_50_ values of these derivatives were higher than 40 μM and were selected for further analysis.

**Table 4:** Effects of Embelin (**1**) and Nitrogenated Embelin Derivatives (**4a**–**4m**, **5a**–**5d**, **6a**–**6m**) on Cell Viabilities in J774 Macrophages and H9c2 Cardiomyocytes^a^

	Cell viability (%)			
	J774 macrophages		H9c2 cardiomyocytes	
Compound	10 μM	20 μM	10 μM	20 μM
**1**	21.3 ± 2.7^e^	15.3 ± 0.8^e^	36.9 ± 5.8^e^	8.3 ± 0.1^e^
**4a**	50.0 ± 5.6^e^	43.7 ± 2.0^e^	94.8 ± 3.5	89.4 ± 0.6^b^
**4b**	99.7 ± 1.1	98.7 ± 1.2	98.1 ± 6.8	95.8 ± 7.7
**4c**	49.1 ± 3.4^e^	39.5 ± 3.6^e^	98.9 ± 3.4	93.9 ± 2.3
**4d**	38.5 ± 5.9^e^	32.9 ± 3.4^e^	99.5 ± 2.7	98.9 ± 6.1
**4e**	57.3 ± 3.8^e^	44.6 ± 3.3^e^	93.4 ± 7.2	83.7 ± 1.5^e^
**4f**	99.5 ± 1.3	97.8 ± 0.6	88.7 ± 5.3^b^	81.1 ± 2.9^e^
**4g**	31.2 ± 5.3^e^	29.6 ± 3.9^e^	72.6 ± 1.1^e^	62.1 ± 1.3^e^
**4h**	84.9 ± 2.6^e^	51.4 ± 2.9^e^	92.5 ± 6.6	75.8 ± 2.3^e^
**4i**	96.2 ± 6.8	72.6 ± 1.7^e^	84.3 ± 6.8^e^	82.2 ± 6.2^e^
**4j**	89.1 ± 5.5^d^	36.9 ± 3.8^e^	68.9 ± 3.6^e^	60.6 ± 4.9^e^
**4k**	55.6 ± 6.7^e^	37.8 ± 4.8^e^	99.7 ± 1.1	90.7 ± 1.5
**4l**	46.7 ± 6.6^e^	33.1 ± 4.5^e^	98.2 ± 6.6	88.3 ± 6.1^b^
**4m**	94.7 ± 4.2	77.2 ± 4.9^e^	96.5 ± 5.5	96.2 ± 4.8
**5a**	56.9 ± 4.0^e^	56.2 ± 2.0^e^	99.0 ± 7.1	83.0 ± 7.0^e^
**5b**	68.4 ± 3.8^e^	48.3 ± 1.7^e^	89.3 ± 9.2^b^	87.4 ± 6.7^c^
**5c**	56.9 ± 5.5^e^	39.4 ± 5.3^e^	61.9 ± 2.7^e^	56.1 ± 3.4^e^
**5d**	65.5 ± 1.9^e^	50.8 ± 7.0^e^	67.8 ± 2.1^e^	57.5 ± 1.4^e^
**6a**	96.3 ± 2.1	76.9 ± 2.8^e^	97.6 ± 7.0	87.8 ± 6.9^c^
**6b**	81.8 ± 4.7^e^	63.9 ± 6.6^e^	99.7 ± 2.1	68.1 ± 4.6^e^
**6c**	83.4 ± 0.5^e^	55.8 ± 5.7^e^	95.0 ± 8.4	74.3 ± 4.8^e^
**6d**	82.1 ± 3.7^e^	74.5 ± 3.9^e^	99.4 ± 0.2	99.3 ± 2.6
**6e**	93.1 ± 4.5	76.6 ± 5.9^e^	96.7 ± 8.1	93.9 ± 0.9
**6f**	79.3 ± 4.6^e^	62.2 ± 4.3^e^	98.3 ± 4.9	84.5 ± 4.3^d^
**6g**	78.4 ± 5.7^e^	42.2 ± 0.3^e^	88.7 ± 10.4^b^	70.6 ± 3.0^e^
**6h**	73.5 ± 2.8^e^	56.7 ± 3.3^e^	75.3 ± 5.0^e^	71.1 ± 2.8^e^
**6i**	93.3 ± 4.3	75.1 ± 2.9^e^	99.8 ± 3.0	92.6 ± 5.4
**6j**	85.2 ± 3.7^e^	64.0 ± 2.6^e^	85.5 ± 6.2^d^	68.4 ± 8.6^e^
**6k**	81.9 ± 4.6^e^	76.3 ± 5.3^e^	99.2 ± 0.8	94.4 ± 6.3
**6l**	82.3 ± 0.3^e^	63.8 ± 4.8^e^	64.8 ± 6.7^e^	55.9 ± 9.4^e^
**6m**	99.6 ± 4.6	86.4 ± 8.7^e^	89.8 ± 7.6^b^	85.9 ± 8.2^d^
control	100	100	100	100

a Results are expressed as mean ± SD. *n* = 3.

b *p* < 0.05.

c *p* < 0.01.

d *p* < 0.001.

e *p* < 0.0001 vs control (untreatred) cells.

**Figure 3:**
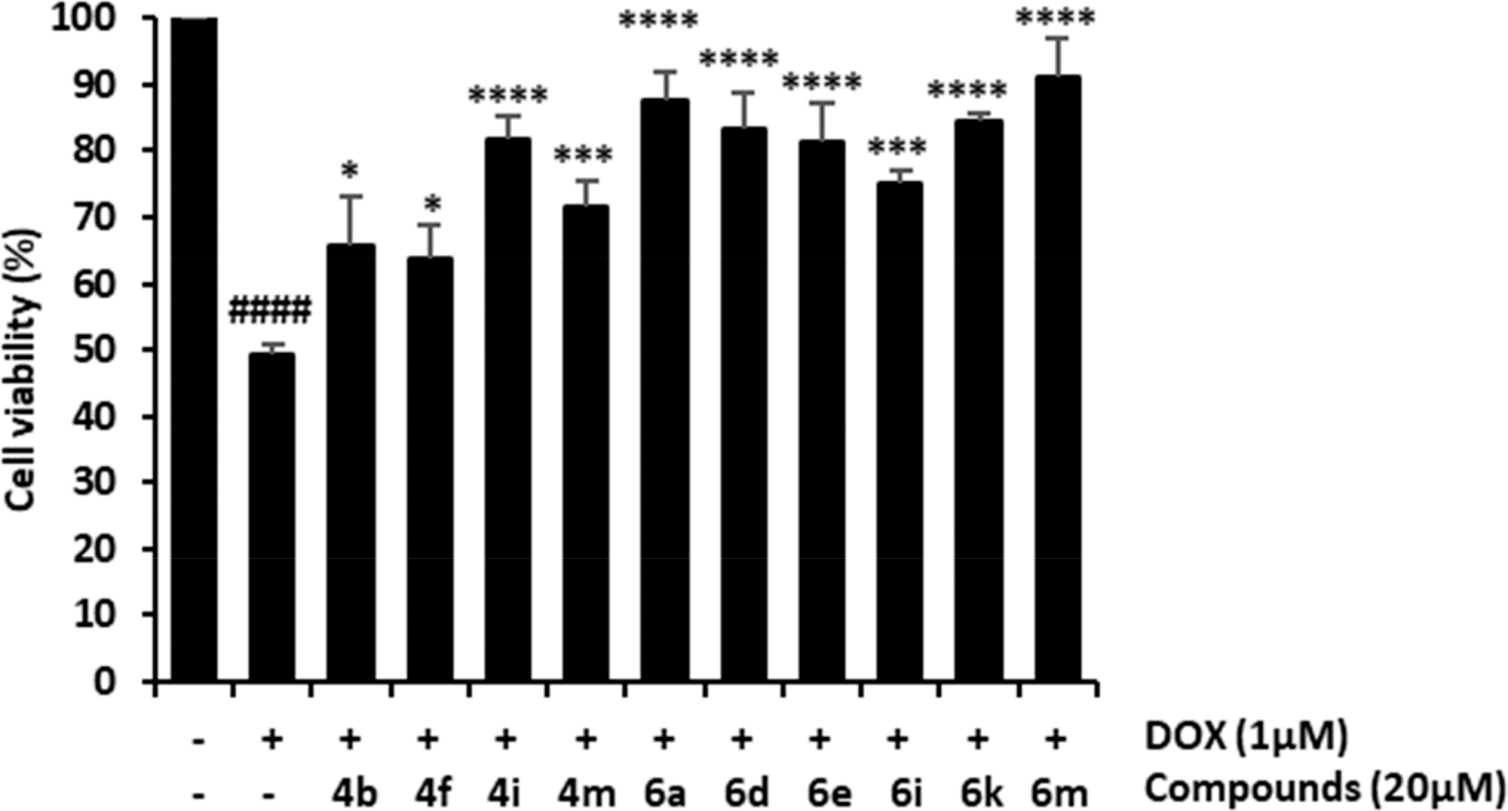
Protective effects of embelin derivatives on DOX-induced cardiotoxicity in H9c2 cells. H9c2 cells were co-treated with DOX (1 μM) and compounds **4b**, **4f**, **4i, 4m**, **6a**, **6d**, **6e**, **6i**, **6k**, and **6m** (20 μM) for 24 h. Cell viability was measured by MTT assay. Data are expressed as the mean ± SD (*n* = 3). *p < 0.05, ***p < 0.001, and ****p < 0.0001 vs DOX-treated cells and ^####^*p* < 0.0001 vs untreated cells.

To evaluate the cardioprotective potential of selected compounds, H9c2 cardiomyocytes were exposed to compounds (20 μM) and doxorubicin (1 μM) for 24 h. A significant loss in cell viability was observed in DOX-treated cells versus untreated cells (IC_50_ = 0.83 μM). Co-treatment with DOX and embelin derivatives significantly attenuated DOX-induced cardiotoxicity. The most prominent cardioprotective effects were observed with compounds **4i**, **6a**, **6d**, **6e**, **6k**, and **6m**, as they significantly increased cell viability reduced by DOX treatment over 80% (81.6 ± 3.7%, 87.5 ± 4.3%, 83.4 ± 5.5%, 81.4 ± 5.9%, 84.3 ± 1.3%, and 91.2 ± 5.9%, respectively) (Figure 3). Derivatives with a quinoline core were more active than the corresponding dihydroquinoline (i.e., **4m** vs **6m**). Regarding the type of substituent at the aromatic ring attached at the pyridine core, the best activities were achieved with electron-withdrawing substituents in the *para* position such as nitro, fluoro, or bromo. The presence of a bromo at the aromatic ring fused to the pyridine led to an improved activity (i.e., **6a** vs **6m**).

**Figure 4:**
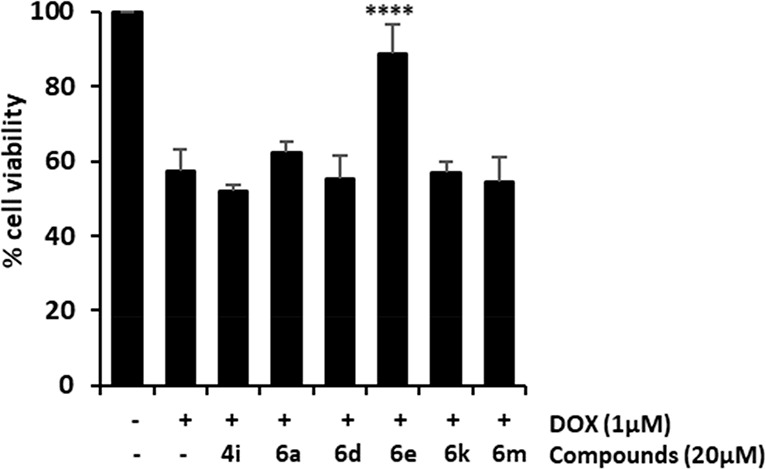
Effects of derivatives **4i**, **6a**, **6d**, **6e**, **6k**, and **6m** on the chemotherapeutic activity of DOX in MCF-7 human breast tumor cells. Cells were co-treated with selected compounds at 20 μM and DOX (1 μM) for 24 h. Cell viability was measured by MTT assay. Results are reported as mean of cell viability ± SD (*n* = 3). ****p < 0.0001 vs DOX-treated cells.

Next, cytotoxicity of the selected compounds was also tested in MCF-7 breast cancer cells to disconfirm that protective effects might antagonize the antitumor effects of doxorubicin. As shown in Figure 4, all compounds, except derivative **6e**, did not affect the antitumor activity of DOX.

Further investigations were focused on the molecular mechanisms of the cardioprotective properties of derivatives **4i**, **6a**, **6d**, **6k**, and **6m**. Multiple pathways have been proposed for the cardiotoxic effects of DOX. Among the reported mechanisms, DOX-induced cardiotoxicity is said to be driven by increased oxidative stress and aggravated apoptosis.^59^

**Figure 5:**
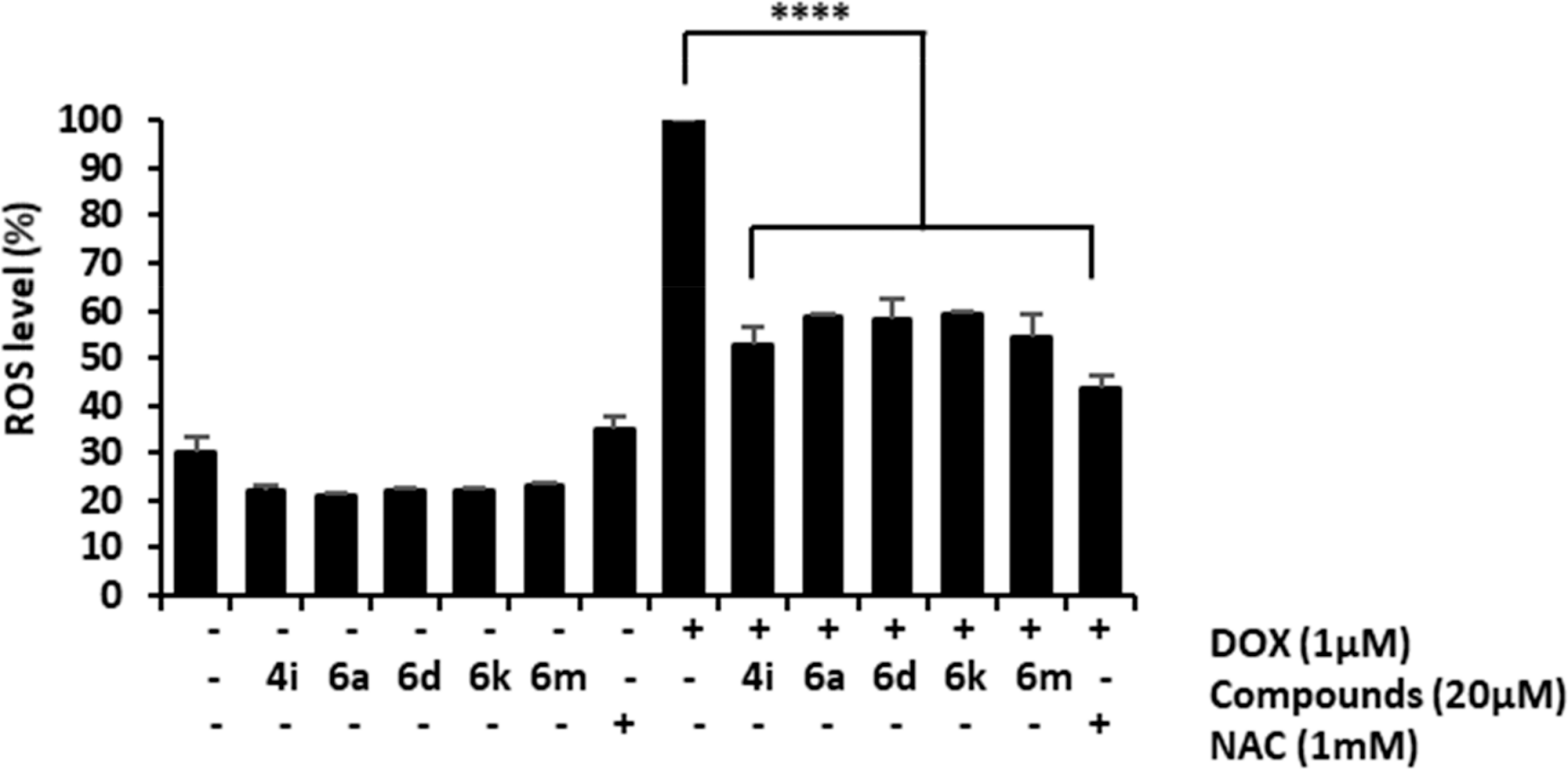
Derivatives **4i**, **6a**, **6d**, **6k**, and **6m** reduced ROS levels produced by DOX in H9c2 cells. Levels of ROS in H9c2 cells were measured fluorometrically after treatment with DOX (1 μM) alone or co-treatment with *N*-acetyl cysteine (NAC, 1 mM) or selected derivatives (20 μM) for 24 h, using the 2′,7′-dichlorfluorescein-diacetate (DCFH-DA) assay. Values are expressed as mean ± SD with respect to DOX-treated cells (*n* = 3). ****p < 0.0001 vs DOX-treated cells.

Oxidative stress is produced by an imbalance between radical oxygen species (ROS) formation and endogenous antioxidant activation in reaction to cell injury, leading to myocardial toxicity. Accordingly, DOX-induced ROS production in the H9c2 cells was quantified with the DCFH-DA fluorogenic dye. DOX significantly increased the levels of ROS compared to untreated cells (Figure 5). No effects on ROS production were observed when compounds were incubated alone with cells. Co-treatment with DOX plus embelin derivatives **4i**, **6a**, **6d**, **6k**, and **6m** significantly inhibited ROS production, reducing Dox-induced oxidative stress. The inhibitory effects of embelin derivatives were similar to those induced by *N*-acetyl-cysteine (NAC), a well-known antioxidant.

The phosphatidylinositol-3-kinase (PI3K/Akt) and mitogen-activated protein kinase (MAPK) signaling pathways play important roles in regulating cell survival. Oxidative stress is involved in the activation of MAPK signaling pathways that leads to DOX-induced cardiomyocyte apoptosis, via phosphorylation of extracellular signal-regulated kinase (ERK).^60^ In contrast, activation of PI3K/Akt has been described to be an important mechanism for cardioprotection.^61^ In order to further understand the cardioprotective mechanism of these five derivatives, we investigated the expression of survival and apoptosis-related proteins (mainly Bcl-2 family genes) following DOX treatment in H9c2 cells.

**Figure 6:**
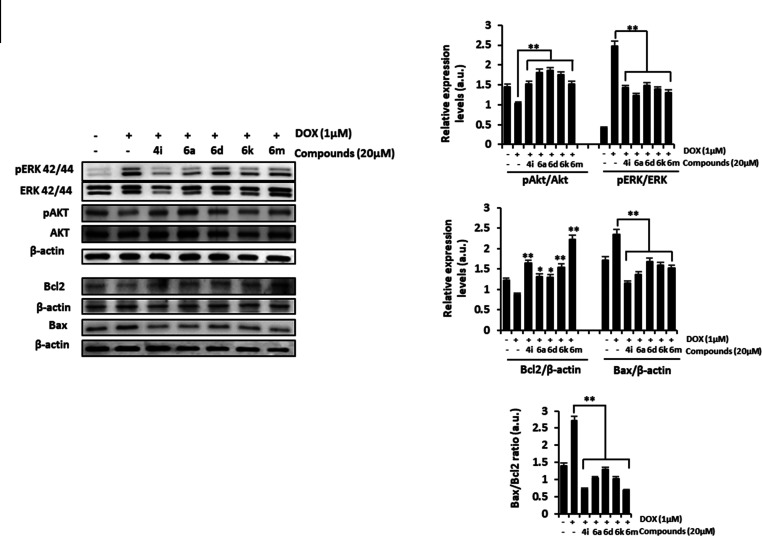
Analysis of survival and apoptotic protein expression in H9c2 cells incubated with derivatives in combination with DOX. Western blot analysis of pAkt, pERK, Bcl2, and Bax protein expression in cells following exposure to DOX (1 μM) alone or in combination with derivatives (20 μM). β-Actin was immunoblotted as a loading control. Densitometric analysis of the relative expression of pERK, pAkt, Bcl2, and Bax. Data are presented as mean ± SD (*n* = 3). *p < 0.05, **p < 0.01 vs DOX-treated cells.

As shown in Figure 6, all active derivatives suppressed ERK phosphorylation induced by DOX, as determined by Western blot analysis. Moreover, co-treatment with DOX and embelin derivatives resulted in a significant activation of Akt. Finally, our results from Western blot also revealed that in the presence of embelin derivatives pro-apoptotic Bax protein expression was decreased while the expression of anti-apoptotic protein Bcl-2 was increased. Collectively, these results indicate that cell damage induced by DOX was reversed after treatment with embelin derivatives, supporting the cardioprotective effects of these compounds.

In conclusion, a set of dihydroquinoline embelin derivatives was synthesized by reaction between embeline (**1**), aromatic aldehydes, and anilines. With improved reaction conditions in hand, the substrate scope of the reaction with respect to aldehyde and aniline was evaluated. Dihydroquinolines treated with DDQ gave the corresponding quinoline derivatives in high yields. Due to previous reports of embelin against myocardial injury, the cardioprotective effects of the synthesized compounds were evaluated. The results indicated that the presence of a quinoline moiety substituted by electron-withdrawing groups seems to play an important role for the activity. Five embelin derivatives, **4i**, **6a**, **6d**, **6k**, and **6m**, could attenuate DOX-induced cardiotoxicity without reducing DOX’s chemotherapeutic effect. Our findings also indicate that these derivatives reduce oxidative stress induced by DOX and may have a potential benefit in preventing cardiotoxicity by doxorubicin.

## Experimental Section

### General Experimental Procedures

IR spectra were obtained using a Cary 630 FTIR spectrometer. UV spectra were obtained in absolute EtOH on a Thermo Scientific Genesys 180 spectrophotometer. NMR spectra were recorded on a Bruker Avance 500 or Bruker Avance 600 in CDCl_3_ or DMSO at 500 or 600 MHz for ^1^H NMR and 125 or 150 MHz for ^13^C NMR. Chemical shifts are given in (δ) parts per million, and coupling constants (*J*) in hertz (Hz). ^1^H and ^13^C spectra were referenced using the solvent signal as internal standard. Melting points were taken on a capillary melting point apparatus and are uncorrected. EIMS and HREIMS were recorded on a Micromass Autospec spectrometer. HREIMS were recorded using a high-resolution magnetic trisector (EBE) mass analyzer. Analytical thin-layer chromatography plates (Polygram-Sil G/UV254) were used. Preparative thin-layer chromatography was carried out with Analtech silica gel GF plates (20 × 20 cm, 1000 μm) using appropriate mixtures of EtOAc and hexanes. All solvents and reagents were purified by standard techniques reported^62^ or used as supplied from commercial sources. All compounds were named using the ACD40 Name-Pro program, which is based on IUPAC rules. The embelin (**1**) used in the reactions was obtained from *Oxalis erythrorhiza* Gillies ex Hook. & Arn. following the procedure described in ref (30).

### General Procedure for the Synthesis of 9,10-Dihydroacridine-1,4-dione Derivatives

In a MW vial equipped with a magnetic stir bar, 20 mg of embelin (0.068 mmol), 1.5 equiv of aldehyde, 1.5 equiv of aniline, and 20 mol % of AgOTf as catalyst were dissolved in 2 mL of EtOH. The MW tube was sealed, and the reaction mixture irradiated at 150 °C for 15 min. The solvent was removed under reduced pressure, and the reaction mixtures were purified by preparative-TLC.

#### 2-Hydroxy-9-(4-nitrophenyl)-3-undecyl-9,10-dihydroacridine-1,4-dione (**4a**)

The reaction mixture purified by preparative TLC with 20% n-Hex/AcOEt yielded 27.4 mg (80%) of **4a** as an amorphous violet solid and 3.2 mg of **5a** (13%) as a red solid. Mp 164–166 °C; UV (EtOH) λ_max_ 290, 340 nm; IR (film) ν_max_ 3317, 2963, 1572, 1513, 1442, 1223, 1081, 798 cm^–1^; ^1^H NMR (600 MHz, DMSO-*d*_6_) δ 0.84 (t, *J* = 7.1 Hz, 3H), 1.22 (bs, 16H), 1.37 (m, 2H), 2.29 (t, *J* = 7.6 Hz, 2H), 5.46 (s, 1H), 6.98 (td, *J* = 0.9, 7.5 Hz, 1H), 7.18 (m, 2H), 7.48 (d, *J* = 8.3 Hz, 1H), 7.52 (d, *J* = 8.8 Hz, 2H), 8.12 (d, *J* = 8.8 Hz, 2H), 10.16 (s, 1H); ^13^C NMR (125 MHz, CDCl_3_) δ 14.1 (CH_3_), 22.5 (CH_2_), 22.7 (CH_2_), 28.1 (CH_2_), 29.3 (CH_2_), 29.4 (CH_2_), 29.5 (CH_2_), 29.6 (CH_2_ × 2), 29.7 (CH_2_), 31.9 (CH_2_), 40.7 (CH), 105.6 (C), 115.1 (C), 116.6 (C), 117.3 (CH), 123.9 (CH × 2), 125.9 (CH), 128.6 (CH), 128.8 (CH × 2), 130.5 (CH), 133.9 (C), 139.3 (C), 146.7 (C), 152.9 (C), 153.9 (C), 178.4 (C), 181.7 (C); EIMS *m*/*z* (%) 502 ([M^+^], 97), 380 (100), 361 (31), 240 (12); HRMS 502.2644 (calcd for C_30_H_34_N_2_O_5_ [M^+^] 502.2468).

#### 2-Hydroxy-5-(phenylamino)-3-undecylcyclohexa-2,5-diene-1,4-dione (**5a**)

^1^H NMR (500 MHz, CDCl_3_) δ 0.88 (t, *J* = 7.1 Hz, 3H), 1.26 (bs, 16H), 1.48 (m, 2H), 2.45 (t, *J* = 7.8 Hz, 2H), 6.00 (s, 1H), 7.24 (m, 3H), 7.42 (t, *J* = 7.6 Hz, 2H), 7.96 (bs, 1H); ^13^C NMR (125 MHz, CDCl_3_) δ 14.1 (CH_3_), 22.7 (CH_2_), 22.8 (CH_2_), 28.1 (CH_2_), 29.3 (CH_2_), 29.4 (CH_2_), 29.5 (CH_2_ × 2), 29.6 (CH_2_), 29.7 (CH_2_), 31.9 (CH_2_), 94.5 (CH), 116.3 (C), 122.8 (CH × 2), 126.3 (CH), 129.7 (CH × 2), 136.9 (C), 146.0 (C), 154.3 (C), 180.4 (C), 182.9 (C); EIMS *m*/*z* (%) 369 ([M^+^], 100), 229 (33), 228 (46), 200 (14); HREIMS 369.2298 (calcd for C_23_H_31_NO_3_ [M^+^] 369.2304).

#### 2-Hydroxy-9-phenyl-3-undecyl-9,10-dihydroacridine-1,4-dione (**4b**)

The reaction mixture purified by PTLC with 20% n-Hex/AcOEt yielded 22.1 mg (70%) of **4b** as an amorphous violet solid (mp 161–162 °C) and 4.2 mg of **5a** (17%) as a red solid. UV (EtOH) λ_max_ 288, 322 nm; IR (film) ν_max_ 3310, 2926, 2363, 1599, 1442, 1222, 798 cm^–1^; ^1^H NMR (500 MHz, CDCl_3_) δ 0.87 (t, *J* = 7.0 Hz, 3H), 1.25 (bs, 16H), 1.45 (m, 2H), 2.39 (t, *J* = 8.3 Hz, 2H), 5.33 (s, 1H), 6.98 (d, *J* = 8.0 Hz, 2H), 7.02 (t, *J* = 7.4 Hz, 1H), 7.11 (d, *J* = 7.6 Hz, 1H), 7.17 (m, 2H), 7.25 (m, 4H), 7.83 (bs, 1H); ^13^C NMR (125 MHz, CDCl_3_) δ 14.1 (CH_3_), 22.5 (CH_2_), 22.7 (CH_2_), 28.1 (CH_2_), 29.3 (CH_2_), 29.4 (CH_2_), 29.5 (CH_2_), 29.6 (CH_2_ × 2), 29.7 (CH_2_), 31.9 (CH_2_), 40.6 (CH), 107.0 (C), 115.9 (C), 116.9 (CH), 125.3 (C), 125.5 (CH), 126.7 (CH), 127.8 (CH × 4), 128.6 (CH), 130.9 (CH), 133.9 (C), 139.1 (C), 146.4 (C), 154.0 (C), 178.5 (C), 182.2 (C); EIMS *m*/*z* (%) 475 ([M^+^], 90), 381 (69), 380 (100), 316 (35), 240 (16); HREIMS 475.2612 (calcd for C_30_H_34_NO_3_F [M^+^] 475.2617).

#### 2-Hydroxy-9-(4-chlorophenyl)-3-undecyl-9,10-dihydroacridine-1,4-dione (**4c**)

The reaction mixture purified by PTLC with 20% n-Hex/AcOEt yielded 24.8 mg (74%) of **4c** as an amorphous violet solid and 2.8 mg of **5a** (11%) as a red solid. Mp 139–141 °C; UV (EtOH) λ_max_ 287, 380 nm; IR (film) ν_max_ 3317, 3239, 2963, 2918, 2363, 1643, 1572, 1223, 798 cm^–1^; ^1^H NMR (500 MHz, CDCl_3_) δ 0.87 (t, *J* = 7.0 Hz, 3H), 1.25 (bs, 16H), 1.44 (m, 2H), 2.40 (t, *J* = 7.7 Hz, 2H), 5.30 (s, 1H), 5.31 (bs, 1H), 5.25 (s, 1H), 6.99 (d, *J* = 8.1 Hz, 1H), 7.03 (t, *J* = 7.6 Hz, 1H), 7.08 (d, *J* = 6.9 Hz, 2H), 7.17 (d, *J* = 8.5 Hz, 2H), 7.20 (m, 3H), 7.84 (bs, 1H); ^13^C NMR (125 MHz, CDCl_3_) δ 14.1 (CH_3_), 22.4 (CH_2_), 22.7 (CH_2_), 28.1 (CH_2_), 29.3 (CH_2_), 29.4 (CH_2_), 29.5 (CH_2_), 29.6 (CH_2_ × 2), 29.7 (CH_2_), 31.9 (CH_2_), 40.1 (CH), 106.5 (C), 116.1 (C), 116.9 (CH), 124.8 (C), 125.7 (CH), 128.1 (CH), 128.7 (CH × 2), 129.2 (CH × 2), 130.6 (CH), 132.6 (C), 133.9 (C), 139.1 (C), 144.9 (C), 153.9 (C), 178.5 (C), 182.0 (C); EIMS *m*/*z* (%) 591 ([M^+^], 64), 380 (100), 349 (22), 238 (20); HREIMS 493.2211 (calcd for C_30_H_34_NO_3_^37^Cl [M^+^] 493.2198), 491.2207 (calcd for C_30_H_34_NO_3_^35^Cl [M^+^] 491.2227).

#### 2-Hydroxy-9-(4-bromophenyl)-3-undecyl-9,10-dihydroacridine-1,4-dione (**4d**)

The reaction mixture purified by PTLC with 20% n-Hex/AcOEt yielded 22.6 mg (62%) of **4d** as an amorphous violet solid and 3.7 mg of **5a** (15%) as a red solid. Mp 139–140 °C; UV (EtOH) λ_max_ 290, 295, 325, 339 nm; IR (film) ν_max_ 3317, 3239, 2963, 2918, 2851, 2363, 1643, 1573, 1442, 1222, 1080, 798 cm^–1^; ^1^H NMR (600 MHz, DMSO-*d*_6_) δ 0.84 (t, *J* = 6.9 Hz, 3H), 1.22 (bs, 16H), 1.37 (m, 2H), 2.30 (t, *J* = 7.8 Hz, 2H), 5.27 (s, 1H), 6.98 (t, *J* = 7.2 Hz, 1H), 7.16 (m, 2H), 7.18 (d, *J* = 8.4 Hz, 2H), 7.42 (d, *J* = 8.4 Hz, 2H), 7.45 (d, *J* = 8.1 Hz, 2H), 10.09 (s, 1H); ^13^C NMR (125 MHz, CDCl_3_) δ 13.92 (CH_3_), 21.83 (CH_2_), 22.05 (CH_2_), 27.54 (CH_2_), 28.68 (CH_2_), 28.79 (CH_2_), 28.89 (CH_2_ × 2), 28.98 (CH_2_), 29.00 (CH_2_), 31.25 (CH_2_), 39.22 (CH), 105.81 (C), 116.00 (C), 117.78 (CH), 119.29 (C), 124.48 (C), 124.50 (CH), 127.54 (CH), 129.48 (CH × 2), 129.71 (CH), 131.22 (CH × 2), 134.83 (C), 139.35 (C), 146.73 (C), 155.61 (C), 178.35 (C), 182.05 (C); EIMS *m*/*z* (%) 535 ([M^+^], 49), 393 (11), 380 (31), 380 (100); HRMS 537.1682 (calcd for C_30_H_34_NO_3_^81^Br [M^+^] 537.1702), 535.1725 (calcd for C_30_H_34_NO_3_^79^Br [M^+^] 535.1722).

#### 2-Hydroxy-9-(4-fluorophenyl)-3-undecyl-9,10-dihydroacridine-1,4-dione (**4e**)

The reaction mixture purified by PTLC with 20% n-Hex/AcOEt yielded 23.2 mg (72%) of **4e** as an amorphous violet solid and 1.6 mg of **5a** (10%) as a red solid. Mp 140–142 °C; UV (EtOH) λ_max_ 289, 294, 340 nm; IR (film) ν_max_ 3317, 3239, 2963, 2918, 2851, 2363, 1643, 1573, 1442, 1222, 798 cm^–1^; ^1^H NMR (500 MHz, CDCl_3_) δ 0.87 (t, *J* = 7.0 Hz, 3H), 1.24 (bs, 16H), 1.44 (m, 2H), 2.39 (t, *J* = 7.7 Hz, 2H), 5.32 (s, 1H), 6.92 (t, *J* = 8.8 Hz, 2H), 6.99 (d, *J* = 7.8 Hz, 1H), 7.03 (t, *J* = 6.7 Hz, 1H), 7.09 (d, *J* = 7.8 Hz, 1H), 7.20 (m, 3H), 7.85 (bs, 1H); ^13^C NMR (125 MHz, CDCl_3_) δ 14.1 (CH_3_), 22.5 (CH_2_), 22.7 (CH_2_), 28.1 (CH_2_), 29.3 (CH_2_), 29.4 (CH_2_), 29.5 (CH_2_), 29.6 (CH_2_ × 2), 29.7 (CH_2_), 31.9 (CH_2_), 39.9 (CH), 106.8 (C), 115.4 (CH × 2, *J*_C–F_ = 21.5 Hz), 116.0 (C), 116.9 (CH), 125.1 (C), 125.6 (CH), 127.9 (CH), 129.4 (CH × 2, *J*_C–F_ = 7.5 Hz), 130.5 (CH), 133.9 (C), 139.0 (C), 154.0 (C), 160.6 (C), 162.5 (C), 178.5 (C), 182.1 (C); EIMS *m*/*z* (%) 475 ([M^+^], 89), 381 (30), 380 (100), 334 (23); HREIMS 475.2513 (calcd for C_30_H_34_NO_3_F [M^+^] 475.2523).

#### 2-Hydroxy-9-(3-fluorophenyl)-3-undecyl-9,10-dihydroacridine-1,4-dione (**4f**)

The reaction mixture purified by PTLC with 20% n-Hex/AcOEt yielded 23.1 mg (72%) of **4f** as an amorphous violet solid and 3.0 mg of **5a** (12%) as a red solid. Mp 240–242 °C; UV (EtOH) λ_max_ 288, 294, 337, 392 nm; IR (film) ν_max_ 3317, 3239, 2963, 2918, 2851, 2363, 1643, 1572, 1513, 1442, 1223, 1080, 798 cm^–1^; ^1^H NMR (500 MHz, CDCl_3_) δ 0.87 (t, *J* = 6.9 Hz, 3H), 1.24 (bs, 16H), 1.45 (m, 2H), 2.40 (t, *J* = 7.9 Hz, 2H), 5.34 (s, 1H), 6.85 (td, *J* = 1.7, 8.2 Hz, 1H), 6.91 (dt, *J* = 2.1, 9.8 Hz, 1H), 7.00 (d, *J* = 7.9 Hz, 2H), 7.05 (m, 2H), 7.11 (d, *J* = 7.1 Hz, 1H), 7.21 (m, 2H), 7.85 (bs, 1H); ^13^C NMR (125 MHz, CDCl_3_) δ 14.1 (CH_3_), 22.5 (CH_2_), 22.7 (CH_2_), 28.1 (CH_2_), 29.3 (CH_2_), 29.4 (CH_2_), 29.5 (CH_2_), 29.6 (CH_2_ × 2), 29.7 (CH_2_), 31.9 (CH_2_), 40.4 (CH), 106.4 (C), 113.7 (CH, *J*_C–F_ = 21.2 Hz), 114.9 (CH, *J*_C–F_ = 21.7 Hz), 116.1 (C), 117.0 (CH), 123.4 (CH, *J*_C–F_ = 2.6 Hz), 124.6 (C), 125.7 (CH), 128.1 (CH), 129.9 (CH, *J*_C–F_ = 8.1 Hz), 130.4 (CH), 133.9 (C), 139.2 (C), 148.7 (C, *J*_C–F_ = 6.3 Hz), 153.9 (C), 163.1 (C, *J*_C–F_ = 245.6 Hz), 178.4 (C), 182.0 (C); EIMS *m*/*z* (%) 475 ([M^+^], 60), 381 (23), 380 (100), 334 (23), 240 (11); HREIMS 475.2519 (calcd for C_30_H_34_NO_3_F [M^+^] 475.2523).

#### Methyl 4-(2-hydroxy-1,4-dioxo-3-undecyl-1,4,9,10-tetrahydroacridin-9-yl)benzoate (**4g**)

The reaction mixture purified by PTLC with 20% n-Hex/AcOEt yielded 28.2 mg (81%) of **4g** as an amorphous violet solid and 3.6 mg of **5a** (14%) as a red solid. Mp 146–148 °C; UV (EtOH) λ_max_ 286, 293, 340, 378 nm; IR (film) ν_max_ 3317, 3239, 2963, 2918, 2851, 2363, 1644, 1573, 1513, 1442, 1223, 1081, 798 cm^–1^; ^1^H NMR (500 MHz, CDCl_3_) δ 0.87 (t, *J* = 7.1 Hz, 3H), 1.25 (bs, 16H), 1.45 (m, 2H), 2.40 (t, *J* = 7.8 Hz, 2H), 3.86 (s, 3H), 5.39 (s, 1H), 7.00 (d, *J* = 8.1 Hz, 1H), 7.04 (dd, *J* = 0.8, 7.5 Hz, 1H), 7.08 (d, *J* = 6.7 Hz, 1H), 7.20 (td, *J* = 1.3, 8.0 Hz, 1H), 7.32 (d, *J* = 8.3 Hz, 2H), 7.88 (bs, 1H), 7.92 (d, *J* = 8.2 Hz, 2H); ^13^C NMR (125 MHz, CDCl_3_) δ 14.1 (CH_3_), 22.5 (CH_2_), 22.7 (CH_2_), 28.1 (CH_2_), 29.3 (CH_2_), 29.4 (CH_2_), 29.5 (CH_2_), 29.6 (CH_2_ × 2), 29.7 (CH_2_), 31.9 (CH_2_), 40.7 (CH), 52.1 (CH_3_), 106.2 (C), 116.1 (C), 117.1 (CH), 122.8 (C), 124.5 (C), 125.7 (CH), 127.9 (CH × 2), 128.2 (CH), 130.0 (CH × 2), 130.5 (CH), 133.9 (C), 139.2 (C), 151.1 (C), 153.9 (C), 166.8 (C), 178.4 (C), 181.9 (C); EIMS *m*/*z* (%) 515 ([M^+^], 90), 381 (39), 380 (100), 374 (22), 240 (10); HREIMS 515.2672 (calcd for C_32_H_37_NO_5_ [M^+^] 515.2672).

#### 2-Hydroxy-9-(thiophen-3-yl)-3-undecyl-9,10-dihydroacridine-1,4-dione (**4h**)

The reaction mixture purified by PTLC with 20% n-Hex/AcOEt yielded 26.1 mg (83%) of **4h** as an amorphous violet solid and 2.6 mg of **5a** (10%) as a red solid. Mp 190–192 °C; UV (EtOH) λ_max_ 290, 295, 360 nm; IR (film) ν_max_ 3317, 3239, 2963, 2918, 2851, 2363, 1644, 1573, 1442, 1223, 1080, 798 cm^–1^; ^1^H NMR (500 MHz, CDCl_3_) δ 0.87 (t, *J* = 7.1 Hz, 3H), 1.25 (bs, 16H), 1.44 (m, 2H), 2.39 (t, *J* = 7.7 Hz, 2H), 5.47 (s, 1H), 6.90 (m, 2H), 6.99 (d, *J* = 7.8 Hz, 1H), 7.08 (t, *J* = 7.4 Hz, 1H), 7.20 (m, 3H), 7.88 (bs, 1H); ^13^C NMR (125 MHz, CDCl_3_) δ 14.1 (CH_3_), 22.5 (CH_2_), 22.7 (CH_2_), 28.1 (CH_2_), 29.3 (CH_2_), 29.4 (CH_2_), 29.5 (CH_2_), 29.6 (CH_2_ × 2), 29.7 (CH_2_), 31.9 (CH_2_), 35.5 (CH), 106.4 (C), 115.9 (C), 116.8 (CH), 121.2 (CH), 124.6 (C), 125.5 (CH), 125.8 (CH), 127.3 (CH), 127.9 (CH), 130.3 (CH), 134.2 (C), 139.2 (C), 146.3 (C), 154.0 (C), 178.5 (C), 182.2 (C); EIMS *m*/*z* (%) 463 ([M^+^], 100), 381 (23), 380 (84), 322 (43), 238 (29); HREIMS 463.2162 (calcd for C_28_H_33_NO_3_S [M^+^] 463.2181).

#### 2-Hydroxy-9-(3,4-dimethylphenyl)-3-undecyl-9,10-dihydroacridine-1,4-dione (**4i**)

The reaction mixture purified by PTLC with 20% n-Hex/AcOEt yielded 17.3 mg (52%) of **4i** as an amorphous violet solid and 5.2 mg of **5a** (21%) as a red solid. Mp 106–108 °C; UV (EtOH) λ_max_ 286, 294, 342, 365 nm; IR (film) ν_max_ 3317, 3250, 2922, 2855, 1643, 1595, 1446, 1223, 1084, 767 cm^–1^; ^1^H NMR (500 MHz, CDCl_3_) δ 0.87 (t, *J* = 7.2 Hz, 3H), 1.25 (bs, 16H), 1.44 (m, 2H), 2.16 (s, 3H), 2.19 (s, 3H), 2.39 (t, *J* = 7.8 Hz, 2H), 5.25 (s, 1H), 6.99 (m, 5H), 7.11 (d, *J* = 7.3 Hz, 1H), 7.16 (dt, *J* = 1.3, 7.5 Hz, 1H), 7.78 (s, 1H); ^13^C NMR (125 MHz, CDCl_3_) δ 14.1 (CH_3_), 19.4 (CH_3_), 19.9 (CH_3_), 22.5 (CH_2_), 22.6 (CH_2_), 28.1 (CH_2_), 29.3 (CH_2_), 29.4 (CH_2_), 29.5 (CH_2_), 29.6 (CH_2_ × 2), 29.7 (CH_2_), 31.9 (CH_2_), 40.2 (CH), 107.1 (C), 115.8 (C), 116.8 (CH), 120.9 (C), 125.2 (CH), 125.5 (CH), 125.6 (C), 127.7 (CH), 129.0 (CH), 130.4 (CH), 133.9 (C), 135.1 (C), 136.8 (C), 139.1 (C), 144.2 (C), 154.1 (C), 178.5 (C), 182.3 (C); EIMS *m*/*z* (%) 485 ([M^+^], 81), 380 (100), 344 (12), 238 (12); HREIMS 485.2926 (calcd for C_32_H_39_O_3_N [M^+^] 485.2930).

#### 2-Hydroxy-9-(3,4-dimethoxyphenyl)-3-undecyl-9,10-dihydroacridine-1,4-dione (**4j**)

The reaction mixture purified by PTLC with 20% n-Hex/AcOEt yielded 13.8 mg (56%) of **4j** as an amorphous violet solid and 6.4 mg of **5a** (25%) as a red solid. Mp 152–154 °C; UV (EtOH) λ_max_ 291, 297, 350 nm; IR (film) ν_max_ 3317, 3239, 2963, 2918, 2851, 2363, 1643, 1573, 1513, 1442, 1222, 1081, 798 cm^–1^; ^1^H NMR (500 MHz, CDCl_3_) δ 0.87 (t, *J* = 7.1 Hz, 3H), 1.25 (bs, 16H), 1.45 (m, 2H), 2.40 (t, *J* = 8.0 Hz, 2H), 3.80 (s, 3H), 3.82 (s, 3H), 5.28 (s, 1H), 6.70 (dd, *J* = 2.0, 8.3 Hz, 1H), 6.82 (d, *J* = 1.9 Hz, 1H), 7.04 (td, *J* = 1.1, 7.5 Hz, 1H), 7.13 (d, *J* = 7.2 Hz, 1H), 7.19 (td, *J* = 1.4, 7.6 Hz, 1H), 7.81 (bs, 1H); ^13^C NMR (150 MHz, CDCl_3_) δ 14.1 (CH_3_), 22.4 (CH_2_), 22.7 (CH_2_), 28.1 (CH_2_), 29.3 (CH_2_), 29.4 (CH_2_), 29.5 (CH_2_), 29.6 (CH_2_ × 2), 29.7 (CH_2_), 31.9 (CH_2_), 40.1 (CH), 55.8 (CH_3_), 55.9 (CH_3_), 107.1 (C), 111.1 (CH), 111.3 (CH), 115.9 (C), 116.8 (CH), 119.9 (CH), 125.4 (C), 125.5 (CH), 127.8 (CH), 130.4 (CH), 133.9 (C), 138.9 (C), 139.4 (C), 147.8 (C), 148.9 (C), 154.0 (C), 178.6 (C), 182.3 (C); EIMS *m*/*z* (%) 517 ([M^+^], 61), 381 (26), 380 (100), 238 (19); HREIMS 517.2839 (calcd for C_32_H_39_NO_5_ [M^+^] 517.2828).

#### 2-Hydroxy-7-methoxy-9-(4-nitrophenyl)-3-undecyl-9,10-dihydroacridine-1,4-dione (**4k**)

The reaction mixture purified by PTLC with 20% n-Hex/AcOEt yielded 24.2 mg (80%) of **4k** as an amorphous violet solid and 3.7 mg of **5b** (14%) as a red solid. Mp 153–155 °C; UV (EtOH) λ_max_ 290, 297, 380 nm; IR (film) ν_max_ 3317, 3239, 2963, 2918, 2851, 2363, 1644, 1573, 1513, 1442, 1222, 1080, 798 cm^–1^; ^1^H NMR (500 MHz, CDCl_3_) δ 0.87 (t, *J* = 7.0 Hz, 3H), 1.24 (bs, 16H), 1.44 (m, 2H), 2.39 (t, *J* = 7.9 Hz, 2H), 3.72 (s, 3H), 5.44 (s, 1H), 6.56 (d, *J* = 2.7 Hz, 1H), 6.80 (dd, *J* = 2.6, 8.7 Hz, 1H), 7.00 (d, *J* = 8.7 Hz, 1H), 7.41 (d, *J* = 8.7 Hz, 2H), 7.93 (bs, 1H), 8.12 (d, *J* = 8.8 Hz, 2H); ^13^C NMR (125 MHz, CDCl_3_) δ 14.1 (CH_3_), 22.5 (CH_2_), 22.7 (CH_2_), 28.0 (CH_2_), 29.3 (CH_2_), 29.4 (CH_2_), 29.5 (CH_2_), 29.6 (CH_2_ × 2), 29.7 (CH_2_), 31.9 (CH_2_), 40.1 (CH), 55.6 (CH_3_), 103.9 (C), 114.4 (CH), 115.4 (CH), 116.1 (C), 118.6 (CH), 123.9 (CH × 2), 125.3 (C), 127.3 (C), 128.8 (CH × 2), 139.1 (C), 146.6 (C), 152.8 (C), 154.3 (C), 157.8 (C), 177.7 (C), 181.7 (C); EIMS *m*/*z* (%) 532 ([M^+^], 86), 463 (18), 410 (100), 307 (64); HREIMS 532.2574 (calcd for C_31_H_36_N_2_O_6_ [M^+^] 532.2573).

#### 2-Hydroxy-5-((4-methoxyphenyl)amino)-3-undecylcyclohexa-2,5-diene-1,4-dione (**5b**)

^1^H NMR (500 MHz, CDCl_3_) δ 0.88 (t, *J* = 6.9 Hz, 3H), 1.26 (bs, 16H), 1.48 (m, 2H), 2.44 (t, *J* = 7.9 Hz, 2H), 3.83 (s, 3H), 5.3 (s, 1H), 6.94 (d, *J* = 8.8 Hz, 2H), 7.16 (d, *J* = 9.0 Hz, 2H), 7.87 (bs, 1H); ^13^C NMR (125 MHz, CDCl_3_) δ 14.1 (CH_3_), 22.7 (CH_2_), 22.8 (CH_2_), 28.1 (CH_2_), 29.4 (CH_2_), 29.5 (CH_2_), 29.6 (CH_2_ × 2), 29.7 (CH_2_), 29.8 (CH_2_), 31.9 (CH_2_), 55.6 (CH_3_), 93.6 (CH), 114.9 (CH × 2), 116.1 (C), 124.8 (CH × 2), 129.5 (C), 146.9 (C), 154.6 (C), 158.1 (C), 179.9 (C), 182.9 (C); EIMS *m*/*z* (%) 399 ([M^+^], 100), 259 (27), 258 (51), 230 (10); HREIMS 399.2401 (calcd for C_24_H_33_NO_4_ [M^+^] 399.2410).

#### 2-Hydroxy-6,8-dimethyl-9-(4-nitrophenyl)-3-undecyl-9,10-dihydroacridine-1,4-dione (**4l**)

The reaction mixture purified by PTLC with 20% n-Hex/AcOEt yielded 33.4 mg (92%) of **4l** as an amorphous violet solid. Mp 260–262 °C; UV (EtOH) λ_max_ 289, 301, 360 nm; IR (film) ν_max_ 3317, 3239, 2963, 2919, 2851, 2363, 1644, 1573, 1513, 1442, 1222, 1080, 798 cm^–1^; ^1^H NMR (500 MHz, CDCl_3_) δ 0.87 (t, *J* = 7.0 Hz, 3H), 1.23 (bs, 16H), 1.42 (m, 2H), 2.06 (s, 3H), 2.30 (s, 3H), 2.36 (t, *J* = 8.0 Hz, 2H), 5.47 (s, 1H), 6.75 (s, 1H), 6.77 (s, 1H), 7.38 (d, *J* = 8.9 Hz, 2H), 7.84 (bs, 1H), 8.09 (d, *J* = 8.7 Hz, 2H); ^13^C NMR (125 MHz, CDCl_3_) δ 14.1 (CH_3_), 19.2 (CH_3_), 20.9 (CH_3_), 22.5 (CH_2_), 22.7 (CH_2_), 28.1 (CH_2_), 29.3 (CH_2_), 29.4 (CH_2_), 29.5 (CH_2_), 29.6 (CH_2_ × 2), 29.7 (CH_2_), 31.9 (CH_2_), 37.7 (CH), 105.9 (C), 115.6 (CH), 116.1 (C), 119.2 (C), 123.8 (CH × 2), 129.1 (CH), 129.3 (CH × 2), 134.5 (C), 137.9 (C), 138.5 (C), 139.1 (C), 146.4 (C), 151.5 (C), 154.1 (C), 178.0 (C), 181.7 (C); EIMS *m*/*z* (%) 530 ([M^+^], 75), 408 (100), 389 (28), 380 (15), 268 (14); HREIMS 530.2769 (calcd. for C_32_H_38_N_2_O_5_ [M^+^] 530.2781).

#### 7-Bromo-2-hydroxy-9-(4-nitrophenyl)-3-undecyl-9,10-dihydroacridine-1,4-dione (**4m**)

The reaction mixture purified by PTLC with 20% n-Hex/AcOEt yielded 23.4 mg (67%) of **4m** as an amorphous violet solid and 8.8 mg of **5c** (29%) as a red solid. Mp 236–237 °C; UV (EtOH) λ_max_ 290, 296, 375 nm; IR (film) ν_max_ 3317, 3239, 2963, 2918, 2851, 2363, 1644, 1573, 1513, 1442, 1222, 1080, 798 cm^–1^; ^1^H NMR (500 MHz, CDCl_3_) 0.87 (t, *J* = 7.2 Hz, 3H), 1.24 (bs, 16H), 1.44 (m, 2H), 2.40 (t, *J* = 7.7 Hz, 2H), 5.41 (s, 1H), 6.93 (d, *J* = 8.9 Hz, 1H), 7.17 (d, *J* = 1.8 Hz, 1H), 7.35 (dd, *J* = 2.0, 8.5 Hz, 1H), 7.40 (d, *J* = 8.6 Hz, 2H), 7.89 (bs, 1H), 8.14 (d, *J* = 8.7 Hz, 2H); ^13^C NMR (125 MHz, CDCl_3_) 14.1 (CH_3_), 22.5 (CH_2_), 22.7 (CH_2_), 28.1 (CH_2_), 29.3 (CH_2_), 29.4 (CH_2_), 29.5 (CH_2_), 29.6 (CH_2_ × 2), 29.7 (CH_2_), 31.9 (CH_2_), 40.5 (CH), 105.7 (C), 116.7 (C), 118.3 (C), 118.7 (CH), 124.1 (CH × 2), 125.6 (C), 128.9 (CH × 2), 131.7 (CH), 133.0 (C), 133.2 (CH), 138.9 (C), 146.9 (C), 152.2 (C), 153.8 (C), 178.7 (C), 181.5 (C); EIMS *m*/*z* (%) 580 ([M^+^], 100), 473 (62), 458 (70), 346 (35), 168 (75); HREIMS 580.1595 (calcd for C_30_H_33_N_2_O_5_^79^Br[M^+^] 580.1573), 582.1529 (calcd for C_30_H_33_N_2_O_5_^81^Br[M^+^] 582.1552).

#### 5-((4-Bromophenyl)amino)-2-hydroxy-3-undecylcyclohexa-2,5-diene-1,4-dione (**5c**)

^1^H NMR (500 MHz, CDCl_3_) δ 0.88 (t, *J* = 7.1 Hz, 3H), 1.26 (bs, 16H), 1.48 (m, 2H), 2.45 (t, *J* = 7.8 Hz, 2H), 5.96 (s, 1H), 7.13 (d, *J* = 8.6 Hz, 2H), 7.54 (d, *J* = 8.6 Hz, 2H), 7.89 (bs, 1H); ^13^C NMR (125 MHz, CDCl_3_) δ 14.1 (CH_3_), 22.7 (CH_2_), 22.8 (CH_2_), 28.1 (CH_2_), 29.4 (CH_2_), 29.5 (CH_2_), 29.6 (CH_2_ × 3), 29.7 (CH_2_), 31.9 (CH_2_), 95.0 (CH), 116.6 (C), 119.3 (C), 124.2 (CH × 2), 132.9 (CH × 2), 136.1 (C), 145.6 (C), 154.2 (C), 180.5 (C), 182.7 (C); EIMS *m*/*z* (%) 448 ([M^+^], 100), 308 (37), 307 (46), 277 (13); HREIMS 447.1428 (calcd for C_23_H_30_NO_3_^79^Br [M^+^] 447.1409), 449.1388 (calcd for C_23_H_30_NO_3_^81^Br [M^+^] 449.1389).

#### 2-Hydroxy-5-((3-nitrophenyl)amino)-3-undecylcyclohexa-2,5-diene-1,4-dione (**5d**)

A 20 mg amount of embelin (0.068 mmol), 14.1 mg of 3-nitroaniline (0.1 mmol), and 3.5 mg of AgOTf (20 mol %) were dissolved in 2 mL of EtOH. The MW tube was sealed, and the reaction mixture was irradiated at 150 °C for 15 min. The reaction product was filtered and washed with n-hex to yield 28.2 mg (100%) of **5d** as an amorphous red solid. ^1^H NMR (500 MHz, CDCl_3_) δ 0.88 (t, *J* = 7.1 Hz, 3H), 1.26 (bs, 16H), 1.48 (m, 2H), 2.47 (t, *J* = 7.8 Hz, 2H), 6.08 (s, 1H), 7.57 (dd, *J* = 1.1, 8.0 Hz, 1H), 7.63 (t, *J* = 8.0 Hz, 1H), 7.78 (s, 1H), 8.01 (s, 1H), 8.09 (ddd, *J* = 1.1, 2.1, 8.0 Hz, 1H), 8.12 (t, *J* = 2.0 Hz, 1H); ^13^C NMR (150 MHz, CDCl_3_) δ 14.1 (CH_3_), 22.7 (CH_2_), 22.8 (CH_2_), 28.1 (CH_2_), 29.4 (CH_2_), 29.5 (CH_2_), 29.6 (CH_2_ × 3), 29.7 (CH_2_), 31.9 (CH_2_), 96.1 (CH), 117.0 (C), 117.1 (CH), 120.5 (CH), 128.0 (CH), 130.7 (CH), 138.5 (C), 144.9 (C), 149.1 (C), 153.8 (C), 180.9 (C), 182.5 (C); EIMS *m*/*z* (%) 414 ([M^+^], 100), 274 (32), 273 (40), 261 (14); HREIMS 414.2170 (calcd for C_23_H_30_N_2_O_5_ [M^+^] 414.2155).

### General Procedure for the Synthesis of Acridine-1,4-dione Derivatives

One equivalent of DDQ was added to the corresponding 9,10-dihydroacridine-1,4-dione derivative dissolved in 3 mL of CH_2_Cl_2_ at room temperature. The reaction mixture was stirred until the disappearance of the starting material. The solution turned from violet to brown-orange. The reaction mixture was washed with saturated NaHCO_3_ and extracted with CH_2_Cl_2_ to obtain the corresponding acridine-1,4-diones.

#### 2-Hydroxy-9-(4-nitrophenyl)-3-undecylacridine-1,4-dione (**6a**)

Yield: 14.4 mg (72%) of compound **6a**, as an orange oil; UV (EtOH) λ_max_ 291, 297, 344 nm; IR (film) ν_max_ 3373, 2989, 2307, 1703, 1155, 1073, 827 cm^–1^; ^1^H NMR (500 MHz, CDCl_3_): 0.87 (t, *J* = 7.2 Hz, 3H), 1.25 (bs, 16H), 1.39 (m, 2H), 2.71 (t, *J* = 7.7 Hz, 2H), 7.41 (d, *J* = 8.9 Hz, 1H), 7.46 (d, *J* = 8.7 Hz, 2H), 7.64 (t, *J* = 7.5 Hz, 2H), 7.95 (d, *J* = 7.7 Hz, 2H), 8.46 (d, *J* = 8.5 Hz, 2H), 8.51 (d, *J* = 8.4 Hz, 1H); ^13^C NMR (125 MHz, CDCl_3_) δ 14.1 (CH_3_), 22.7 (CH_2_), 23.8 (CH_2_), 28.1 (CH_2_), 29.3 (CH_2_), 29.4 (CH_2_), 29.5 (CH_2_), 29.6 (CH_2_), 29.7 (CH_2_ × 2), 31.9 (CH_2_), 114.7 (C), 123.9 (CH × 2), 127.3 (CH), 127.6 (C), 127.9 (C), 129.2 (CH × 2), 130.1 (CH), 132.0 (CH), 133.5 (CH), 143.1 (C), 147.6 (C), 147.9 (C), 149.2 (C), 149.4 (C), 154.3 (C), 180.4 (C), 182.2 (C); EIMS *m*/*z* (%) 500 ([M^+^], 100), 472 (10), 372 (21), 359 (26); HREIMS 500.2337 (calcd for C_30_H_32_N_2_O_5_ [M^+^] 500.2311).

#### 2-Hydroxy-9-phenyl-3-undecylacridine-1,4-dione (**6b**)

Yield: 14.7 mg (81%) of compound **6b**, as an orange oil; UV (EtOH) λ_max_ 290, 294 nm; IR (film) ν_max_ 3239, 2963, 2918, 2851, 2363, 1662, 1572, 1513, 1442, 1222, 1080, 798 cm^–1^; ^1^H NMR (500 MHz, CDCl_3_) δ 0.87 (t, *J* = 7.3 Hz, 3H), 1.25 (bs, 16H), 1.39 (m, 2H), 2.70 (t, *J* = 7.5 Hz, 2H), 7.25 (m, 2H), 7.57 (m, 5H), 7.69 (m, 1H), 6.43 (t, *J* = 8.2 Hz, 1H), 8.47 (d, *J* = 8.5 Hz, 1H); ^13^C NMR (125 MHz, CDCl_3_) δ 14.1 (CH_3_), 22.7 (CH_2_), 23.7 (CH_2_), 28.1 (CH_2_), 29.3 (CH_2_), 29.5 (CH_2_), 29.6 (CH_2_ × 2), 29.7 (CH_2_ × 2), 31.9 (CH_2_), 119.9 (C), 126.7 (C), 127.9 (CH × 2), 128.3 (CH), 128.4 (CH), 128.5 (CH × 2), 128.9 (C), 129.5 (CH), 131.7 (CH), 133.1 (CH), 136.0 (C), 147.8 (C), 149.3 (C), 152.2 (C), 154.4 (C), 180.5 (C), 182.6 (C); EIMS *m*/*z* (%) 455 ([M^+^], 100), 427 (10), 328 (32), 286 (34); HREIMS 455.2445 (calcd for C_30_H_33_NO_3_ [M^+^] 455.2460).

#### 2-Hydroxy-9-(4-chlorophenyl)-3-undecylacridine-1,4-dione (**6c**)

Yield: 9.2 mg (94%) of compound **6c**, as an orange oil; UV (EtOH) λ_max_ 290, 294, 344 nm; IR (film) ν_max_ 3317, 3239, 2963, 2918, 2851, 2363, 1662, 1573, 1513, 1442, 1223, 1081, 798 cm^–1^; ^1^H NMR (500 MHz, CDCl_3_) δ 0.87 (t, *J* = 7.1 Hz, 3H), 1.25 (bs, 16H), 1.38 (m, 2H), 2.70 (t, *J* = 7.4 Hz, 2H), 7.20 (d, *J* = 7.9 Hz, 2H), 7.53 (d, *J* = 8.5 Hz, 1H), 7.56 (d, *J* = 8.1 Hz, 2H), 7.61 (t, *J* = 7.4 Hz, 1H), 7.92 (t, *J* = 7.2 Hz, 1H), 8.48 (d, *J* = 8.5 Hz, 1H); ^13^C NMR (125 MHz, CDCl_3_) δ 14.1 (CH_3_), 22.7 (CH_2_), 23.7 (CH_2_), 28.1 (CH_2_), 29.3 (CH_2_), 29.4 (CH_2_), 29.5 (CH_2_), 29.6 (CH_2_ × 2), 29.7 (CH_2_), 31.9 (CH_2_), 116.9 (C), 119.9 (C), 127.0 (C), 127.9 (CH), 128.9 (CH × 2), 129.4 (CH × 2), 129.7 (CH), 131.8 (CH), 133.2 (CH), 134.4 (C), 134.7 (C), 147.7 (C), 149.3 (C), 150.8 (C), 154.4 (C), 180.5 (C), 182.9 (C); EIMS *m*/*z* (%) 589 ([M^+^], 100), 380 (19), 351 (23), 319 (25); HREIMS 489.2055 (calcd for C_30_H_32_NO_3_^35^Cl [M^+^] 489.2071), 491.2024 (calcd for C_30_H_32_NO_3_^37^Cl [M^+^] 491.2041).

#### 2-Hydroxy-9-(4-bromophenyl)-3-undecylacridine-1,4-dione (**6d**)

Yield: 12.1 mg (61%) of compound **6d**, as an orange oil; UV (EtOH) λ_max_ 291, 299, 342 nm; IR (film) ν_max_ 3373, 2989, 2307, 1703, 1662, 1551, 1372, 1155, 827 cm^–1^; ^1^H NMR (500 MHz, CDCl_3_) δ 0.87 (t, *J* = 7.1 Hz, 3H), 1.25 (bs, 16H), 1.59 (m, 2H), 2.70 (t, *J* = 7.2 Hz, 2H), 7.14 (d, *J* = 7.9 Hz, 2H), 7.53 (d, *J* = 8.0 Hz, 1H), 7.62 (t, *J* = 7.1 Hz, 1H), 7.72 (d, *J* = 7.7 Hz, 2H), 7.92 (t, *J* = 7.7 Hz, 1H), 8.47 (d, *J* = 7.4 Hz, 1H); ^13^C NMR (125 MHz, CDCl_3_) δ 14.1 (CH_3_), 22.7 (CH_2_), 23.7 (CH_2_), 28.1 (CH_2_), 29.3 (CH_2_), 29.4 (CH_2_), 29.5 (CH_2_), 29.6 (CH_2_ × 2), 29.7 (CH_2_), 31.9 (CH_2_), 119.8 (C), 122.8 (C), 127.0 (C), 127.9 (CH), 128.6 (C), 129.6 (CH × 2), 129.7 (CH), 131.8 (CH × 2), 132.9 (CH), 133.2 (CH), 134.9 (C), 147.7 (C), 149.3 (C), 150.8 (C), 154.4 (C), 180.5 (C), 182.5 (C); EIMS *m*/*z* (%) 535 ([M^+^], 100), 533 (96), 407 (26), 394 (32), 365 (29); HREIMS 535.1563 (calcd for C_30_H_32_NO_3_^81^Br [M^+^] 535.1545), 533.1578 (calcd for C_30_H_32_NO_3_^79^Br [M^+^] 533.1566).

#### 2-Hydroxy-9-(4-fluorophenyl)-3-undecylacridine-1,4-dione (**6e**)

Yield: 15.4 mg (78%) of compound **6e**, as an orange oil; UV (EtOH) λ_max_ 290, 296, 344, 390 nm; IR (film) ν_max_ 3056, 2986, 2683, 2303, 1423.8, 1267.3, 894.6 cm^–1^; ^1^H NMR (500 MHz, CDCl_3_) δ 0.87 (t, *J* = 6.9 Hz, 3H), 1.25 (bs, 16H), 1.39 (m, 2H), 2.70 (t, *J* = 7.2 Hz, 2H), 7.23 (m, 2H), 7.29 (m, 2H), 7.54 (d, *J* = 8.4 Hz, 1H), 7.61 (t, *J* = 7.1 Hz, 1H), 7.91 (t, *J* = 7.1 Hz, 1H), 8.47 (d, *J* = 8.4 Hz, 1H); ^13^C NMR (125 MHz, CDCl_3_) δ 14.1 (CH_3_), 22.7 (CH_2_), 23.7 (CH_2_), 28.1 (CH_2_), 29.3 (CH_2_), 29.4 (CH_2_), 29.5 (CH_2_), 29.6 (CH_2_), 29.7 (CH_2_ × 2), 31.9 (CH_2_), 32.6 (CH), 115.8 (CH × 2, *J*_C–F_ = 21.9 Hz), 120.0 (C), 126.9 (C), 127.9 (CH), 128.9 (C), 129.6 (CH), 129.8 (CH × 2, *J*_C–F_ = 8.1 Hz), 131.8 (CH), 133.2 (CH), 147.8 (C), 149.3 (C), 151.2 (C), 154.4 (C), 162.8 (C, *J*_C–F_ = 248.8 Hz), 180.5 (C), 182.5 (C); EIMS *m*/*z* (%) 473 ([M^+^], 100), 346 (23), 335 (27), 304 (28); HREIMS 473.2381 (calcd for C_30_H_32_NO_3_F [M^+^] 473.2366).

#### 2-Hydroxy-9-(3-fluorophenyl)-3-undecylacridine-1,4-dione (**6f**)

Yield: 16.4 mg (91%) of compound **6f**, as an orange oil; UV (EtOH) λ_max_ 290, 294, 344, 395 nm; IR (film) ν_max_ 3056, 2985, 2684, 2304, 1662, 1423, 1267, 894 cm^–1^; ^1^H NMR (500 MHz, CDCl_3_) δ 0.87 (t, *J* = 7.0 Hz, 3H), 1.25 (bs, 16H), 1.39 (m, 2H), 2.70 (t, *J* = 7.6 Hz, 2H), 6.99 (d, *J* = 8.7 Hz, 1H), 7.03 (d, *J* = 7.3 Hz, 1H), 7.29 (m, 1H), 6.40 (s, 1H), 7.55 (m, 2H), 7.62 (t, *J* = 7.3 Hz, 1H), 7.92 (m, 1H), 8.48 (d, *J* = 8.5 Hz, 1H); ^13^C NMR (125 MHz, CDCl_3_) δ 14.1 (CH_3_), 22.7 (CH_2_), 23.7 (CH_2_), 28.1 (CH_2_), 29.3 (CH_2_), 29.4 (CH_2_), 29.5 (CH_2_), 29.6 (CH_2_), 29.7 (CH_2_ × 2), 31.9 (CH_2_), 115.3 (CH, *J*_C–F_ = 22.3 Hz), 115.5 (CH, *J*_C–F_ = 20.9 Hz), 119.8 (C), 123.7 (CH, *J*_C–F_ = 2.9 Hz), 127.0 (C), 127.9 (CH), 128.5 (C), 129.7 (CH), 130.3 (CH, *J*_C–F_ = 8.3 Hz), 131.8 (CH), 133.3 (CH), 138.1 (C, *J*_C–F_ = 7.9 Hz), 147.7 (C), 149.3 (C), 150.3 (C), 154.4 (C), 162.9 (C, *J*_C–F_ = 250.5 Hz), 180.3 (C), 182.5 (C); EIMS *m*/*z* (%) 473 ([M^+^], 100), 345 (39), 335 (46), 304 (20); HREIMS 473.2351 (calcd for C_30_H_32_NO_3_F [M^+^] 473.2366).

#### Methyl 4-(2-hydroxy-1,4-dioxo-3-undecyl-1,4-dihydroacridin-9-yl)benzoate (**6g**)

Yield: 14.0 mg (78%) of compound **6g**, as an orange oil; UV (EtOH) λ_max_ 292, 297, 344 nm; IR (film) ν_max_ 3056, 2985, 2303, 1662, 1424, 894 cm^–1^; ^1^H NMR (500 MHz, CDCl_3_) δ 0.87 (t, *J* = 7.1 Hz, 3H), 1.25 (bs, 16H), 1.38 (m, 2H), 2.70 (t, *J* = 7.6 Hz, 2H), 4.00 (s, 3H), 7.35 (d, *J* = 7.6 Hz, 2H), 7.47 (d, *J* = 8.3 Hz, 1H), 7.60 (t, *J* = 7.3 Hz, 1H), 7.92 (d, *J* = 7.3 Hz, 1H), 8.27 (d, *J* = 7.8 Hz, 2H), 8.49 (d, *J* = 8.1 Hz, 1H); ^13^C NMR (150 MHz, CDCl_3_) δ 14.1 (CH_3_), 22.7 (CH_2_), 23.7 (CH_2_), 28.1 (CH_2_), 29.3 (CH_2_), 29.5 (CH_2_), 29.6 (CH_2_), 29.6 (CH_2_), 29.7 (CH_2_ × 2), 31.9 (CH_2_), 52.4 (CH_3_), 119.7 (C), 122.8 (C), 127.1 (C), 127.8 (CH), 128.1 (CH × 2), 129.7 (CH), 129.8 (CH × 2), 130.3 (C), 131.8 (CH), 133.3 (CH), 140.9 (C), 147.7 (C), 149.3 (C), 150.9 (C), 154.4 (C), 166.6 (C), 180.4 (C), 182.4 (C); EIMS *m*/*z* (%) 513 ([M^+^], 100), 386 (20), 369 (40), 344 (16), 228 (18); HREIMS 513.2520 (calcd for C_32_H_35_NO_5_ [M^+^] 513.2515).

#### 2-Hydroxy-9-(thiophen-3-yl)-3-undecylacridine-1,4-dione (**6h**)

Yield: 14.6 mg (73%) of compound **6h**, as an orange oil; UV (EtOH) λ_max_ 291, 296, 345, 390; IR (film) ν_max_ 3056, 2985, 2304, 1666, 1550, 1423, 1267, 894 cm^–1^; ^1^H NMR (500 MHz, CDCl_3_) δ 0.87 (t, *J* = 7.0 Hz, 3H), 1.25 (bs, 16H), 1.39 (m, 2H), 2.70 (t, *J* = 7.7 Hz, 2H), 7.07 (d, *J* = 4.6 Hz, 1H), 7.25 (dd, *J* = 1.1, 2.9 Hz, 1H), 7.62 (m, 2H), 7.70 (d, *J* = 8.3 Hz, 1H), 7.91 (t, *J* = 7.2 Hz, 1H), 8.46 (d, *J* = 8.5 Hz, 1H); ^13^C NMR (125 MHz, CDCl_3_) δ 14.1 (CH_3_), 22.7 (CH_2_), 23.7 (CH_2_), 28.1 (CH_2_), 29.3 (CH_2_), 29.4 (CH_2_), 29.5 (CH_2_), 29.6 (CH_2_), 29.7 (CH_2_ × 2), 31.9 (CH_2_), 120.5 (C), 123.7 (CH), 126.1 (CH), 126.7 (C), 128.1 (CH), 128.4 (CH), 129.3 (C), 129.6 (CH), 131.7 (CH), 133.4 (CH), 135.1 (C), 147.8 (C), 147.9 (C), 149.3 (C), 154.5 (C), 180.4 (C), 182.6 (C); EIMS *m*/*z* (%) 461 ([M^+^], 100), 334 (26), 323 (25), 292 (35); HREIMS 461.2015 (calcd for C_28_H_31_NO_3_S [M^+^] 461.2025).

#### 2-Hydroxy-9-(3,4-dimethylphenyl)-3-undecylacridine-1,4-dione (**6i**)

Yield: 17.6 mg (88%) of compound **6i**, as an orange oil; UV (EtOH) λ_max_ 288, 325, 340 nm; IR (film) ν_max_ 3056, 2985, 2929, 2855, 2303, 1662, 1551, 1424, 1267, 895 cm^–1^; ^1^H NMR (500 MHz, CDCl_3_) δ 0.87 (t, *J* = 7.2 Hz, 3H), 1.25 (bs, 16H), 1.39 (m, 2H), 2.36 (s, 3H), 2.43 (s, 3H), 2.70 (t, *J* = 7.7 Hz, 2H), 6.98 (d, *J* = 7.1 Hz, 1H), 7.01 (s, 1H), 7.35 (d, *J* = 7.3 Hz, 1H), 7.58 (m, 2H), 7.89 (t, *J* = 7.9 Hz, 1H), 8.46 (d, *J* = 8.1 Hz, 1H); ^13^C NMR (125 MHz, CDCl_3_) δ 14.1 (CH_3_), 19.8 (CH_3_), 19.9 (CH_3_), 22.7 (CH_2_), 23.7 (CH_2_), 28.1 (CH_2_), 29.3 (CH_2_), 29.4 (CH_2_), 29.5 (CH_2_), 29.6 (CH_2_), 29.7 (CH_2_ × 2), 31.9 (CH_2_), 119.9 (C), 125.3 (CH), 126.6 (C), 128.5 (CH), 128.9 (CH), 129.2 (C), 129.3 (CH), 129.7 (CH), 131.6 (CH), 132.9 (CH), 133.4 (C), 136.7 (C), 136.9 (C), 147.8 (C), 149.2 (C), 152.8 (C), 154.5 (C), 180.6 (C), 182.7 (C); EIMS *m*/*z* (%) 483 ([M^+^], 100), 356 (27), 343 (22), 314 (30); HREIMS 483.2757 (calcd for C_32_H_37_NO_3_ [M^+^] 483.2773).

#### 2-Hydroxy-9-(3,4-dimethoxyphenyl)-3-undecylacridine-1,4-dione (**6j**)

Yield: 9.6 mg (81%) of compound **6j**, as an orange oil; UV (EtOH) λ_max_ 291, 297, 342 nm; IR (film) ν_max_ 3056, 2986, 2303, 1662, 1551, 1423, 1267, 895 cm^–1^; ^1^H NMR (500 MHz, CDCl_3_) δ 0.87 (t, *J* = 7.3 Hz, 3H), 1.25 (bs, 16H), 1.40 (m, 2H), 2.70 (t, *J* = 7.7 Hz, 2H), 3.86 (s, 3H), 4.02 (s, 3H), 6.75 (s, 1H), 6.80 (d, *J* = 8.3 Hz, 1H), 7.08 (d, *J* = 8.2 Hz, 1H), 7.62 (m, 2H), 7.90 (t, *J* = 6.8 Hz, 1H), 8.47 (d, *J* = 8.6 Hz, 1H); ^13^C NMR (125 MHz, CDCl_3_) δ 14.1 (CH_3_), 22.7 (CH_2_), 23.7 (CH_2_), 28.1 (CH_2_), 29.3 (CH_2_), 29.4 (CH_2_), 29.5 (CH_2_), 29.6 (CH_2_), 29.7 (CH_2_ × 2), 31.9 (CH_2_), 55.9 (CH_3_), 56.0 (CH_3_), 110.9 (CH), 111.4 (CH), 120.0 (C), 120.4 (CH), 126.6 (C), 128.2 (C), 128.4 (CH), 129.3 (C), 129.5 (CH), 131.7 (CH), 133.0 (CH), 147.9 (C), 149.1 (C), 149.2 (C), 149.3 (C), 152.2 (C) 154.5 (C), 180.5 (C), 182.7 (C); EIMS *m*/*z* (%) 515 ([M^+^], 100), 397 (35), 369 (37), 229 (36); HREIMS 515.2686 (calcd for C_32_H_37_NO_5_ [M^+^] 515.2672).

#### 2-Hydroxy-7-methoxy-9-(4-nitrophenyl)-3-undecylacridine-1,4-dione (**6k**)

Yield: 16.7 mg (83%) of compound **6k**, as an orange oil; UV (EtOH) λ_max_ 290, 295 nm; IR (film) ν_max_ 3056, 2926, 2855, 2307, 1662, 1520, 1267, 857 cm^–1^; ^1^H NMR (500 MHz, CDCl_3_) δ 0.86 (t, *J* = 7.1 Hz, 3H), 1.24 (bs, 16H), 1.38 (m, 2H), 2.68 (t, *J* = 7.7 Hz, 2H), 3.72 (s, 3H), 6.54 (s, 1H), 7.45 (d, *J* = 8.2 Hz, 2H), 7.57 (dd, *J* = 2.6, 9.2 Hz, 1H), 8.38 (d, *J* = 7.1 Hz, 1H), 8.46 (d, *J* = 8.5 Hz, 2H); ^13^C NMR (125 MHz, CDCl_3_) δ 14.1 (CH_3_), 22.7 (CH_2_), 23.7 (CH_2_), 28.1 (CH_2_), 29.3 (CH_2_), 29.4 (CH_2_), 29.5 (CH_2_), 29.6 (CH_2_), 29.7 (CH_2_ × 2), 31.9 (CH_2_), 55.7 (CH_3_), 104.8 (CH), 119.9 (C), 124.0 (CH × 2), 126.1 (CH), 127.8 (C), 128.8 (C), 129.1 (CH × 2), 129.5 (C), 133.5 (CH), 143.6 (C), 145.3 (C), 147.9 (C), 153.9 (C), 160.4 (C), 160.9 (C), 180.7 (C), 182.4 (C); EIMS *m*/*z* (%) 530 ([M^+^], 100), 399 (50), 390 (38), 258 (40), 221 (42); HREIMS 530.2426 (calcd for C_31_H_34_N_2_O_6_ [M^+^] 530.2417).

#### 2-Hydroxy-6,8-dimethyl-9-(4-nitrophenyl)-3-undecylacridine-1,4-dione (**6l**)

Yield: 17.5 mg (71%) of compound **6l**, as an orange oil; UV (EtOH) λ_max_ 289, 293, 350 nm; IR (film) ν_max_ 3056, 2986, 2863, 2304, 1662, 1551, 1424, 1267, 895 cm^–1^; ^1^H NMR (500 MHz, CDCl_3_) δ 0.87 (t, *J* = 7.1 Hz, 3H), 1.24 (bs, 16H), 1.37 (m, 2H), 1.88 (s, 3H), 2.54 (s, 3H), 2.64 (t, *J* = 7.5 Hz, 2H), 7.26 (s, 1H), 7.52 (d, *J* = 8.1 Hz, 2H), 8.14 (s, 1H), 8.39 (d, *J* = 8.2 Hz, 2H); ^13^C NMR (125 MHz, CDCl_3_) δ 14.1 (CH_3_), 21.7 (CH_3_), 22.7 (CH_2_), 23.6 (CH_2_), 24.9 (CH_3_), 28.0 (CH_2_), 29.3 (CH_2_), 29.4 (CH_2_), 29.5 (CH_2_), 29.6 (CH_2_), 29.7 (CH_2_ × 2), 31.9 (CH_2_), 119.7 (C), 123.5 (CH × 2), 124.5 (C), 126.4 (C), 129.1 (C), 129.2 (CH × 2), 130.6 (CH), 136.6 (CH), 137.6 (C), 144.5 (C), 146.8 (C), 147.9 (C), 149.5 (C), 151.0 (C), 154.4 (C), 180.5 (C), 182.1 (C); EIMS *m*/*z* (%) 528 ([M^+^], 100), 483 (6), 388 (21), 359 (16); HRIMS 528.2648 (calcd for C_32_H_36_N_2_O_5_ [M^+^] 528.2624).

#### 7-Bromo-2-hydroxy-9-(4-nitrophenyl)-3-undecylacridine-1,4-dione (**6m**)

Yield: 5.4 mg (72%) of compound **6m**, as an orange oil; UV (EtOH) λ_max_ 291, 297, 350 nm; IR (film) ν_max_ 3054, 2989, 2926, 2855, 2307, 1662, 1520, 1423, 1267, 895 cm^–1^; ^1^H NMR (500 MHz, CDCl_3_) 0.87 (t, *J* = 7.1 Hz, 3H), 1.25 (bs, 16H), 1.39 (m, 2H), 2.71 (t, *J* = 7.7 Hz, 2H), 7.44 (d, *J* = 8.6 Hz, 2H), 7.51 (d, *J* = 1.9 Hz, 1H), 8.01 (dd, *J* = 2.1, 8.9 Hz, 1H), 8.37 (d, *J* = 8.9 Hz, 1H), 8.48 (d, *J* = 8.5 Hz, 2H); ^13^C NMR (125 MHz, CDCl_3_) 14.1 (CH_3_), 22.7 (CH_2_), 23.8 (CH_2_), 28.1 (CH_2_), 29.4 (CH_2_), 29.4 (CH_2_), 29.5 (CH_2_), 29.6 (CH_2_), 29.7 (CH_2_ × 2), 31.9 (CH_2_), 116.7 (C), 120.3 (C), 124.1 (CH × 2), 125.1 (C), 127.9 (C), 129.1 (CH × 2), 129.3 (CH), 133.4 (CH), 137.1 (CH), 142.2 (C), 147.7 (C), 147.9 (C), 148.1 (C), 148.2 (C), 154.3 (C), 180.2 (C), 181.9 (C); EIMS *m*/*z* (%) 580 ([M^+^], 100), 578 (90), 528 (12), 439 (41); HREIMS 580.1388 (calcd for C_30_H_31_NO_5_^81^Br [M^+^] 580.1396), 578.1396 (calcd for C_30_H_31_NO_5_^79^Br [M^+^] 578.1416).

### Biological Assays

#### Cell Culture

J774A.1 murine macrophage cells, H9c2 embryonic rat heart-derived cells, and MCF-7 breast cancer cells were purchased from American Type Cell Culture (ATCC, Manassas, VA, USA). Cells were cultured in Dulbecco’s modified Eagle medium with 10% fetal bovine serum and 1% penicillin/streptomycin (Lonza) and were maintained at 37 °C in a humidified incubator containing 5% CO_2_.

#### Cell Viability Assay

Cell viability was assessed by conducting an MTT [3-(4,5-dimethylthiazol-2-yl)-2,5-diphenyltetrazolium bromide dye] assay. Briefly, J 774A.1, H9c2, and MCF7 cells were seeded in 96-well plates for 24 h. Then, cells were treated with DOX in the absence or presence of compounds. MTT (Sigma) reagent was added to the medium for 1 h at 37 °C. Then, the formazan was dissolved in dimethyl sulfoxide (DMSO, 100 μL). Absorbance was measured at 540 nm with a microplate reader (BMG Labtech).

#### Assessment of Cardioprotective Activity

Compounds were tested in a DOX-induced H9c2 cardiomyocytes model. H9c2 cells treated with DOX (1 μM) served as a positive control. To determine the potential protective effects, cells were co- treated with compounds at 20 and 1 μM of DOX (Sigma) for 24 h. After that, the MTT cell viability assay was performed.

#### Measurement of Reactive Oxygen Species

Intracellular ROS levels in H9c2 cells were evaluated by monitoring the oxidation of 2′,7′-dichlorfluorescein-diacetate (DCFH-DA) (Sigma) to fluorescent dichlorofluorescein. Cells were treated with DOX (1 μM) in the absence or presence of compounds or NAC as ROS inhibitor (1 mM) (Sigma). Then, cells were incubated with 10 μM DCFH-DA at 37 °C in the dark for 30 min. ROS level was fluorimetrically evaluated at 485/550 nm during 24 h.

#### Western Blot

Cell lysates were prepared as described previously^63^ and later subjected to sulfate-polyacrylamide (SDS-PAGE) electrophoresis. The gels were transferred onto a Hybond-PVDF membrane and, after blocking, were incubated with anti-pAkt, anti-Akt, anti-pERK1/2, anti-ERK1/2, anti-Bax, and anti-Bcl-2. β-Actin (Sigma) were used as a loading control. After further incubating with horseradish peroxidase (HRP)-conjugated secondary antibodies for 2 h, the specific proteins bands were developed by an ECL detection system (Amersham).

#### Statistical Analysis

Statistical analyses were carried out using GraphPad Prism (version 9). Data are presented as means ± standard deviation (SD) from at least three experiments, and a one-way ANOVA was performed. *p* < 0.05 was considered statistically significant.

## Supporting Information Available

Spectra of compounds (PDF)
