# Supplementary material for: Synthesis of Quinoline and Dihydroquinoline Embelin Derivatives as Cardioprotective Agents
Source: J Nat Prod. 2023 Feb 7;86(2):317–29. doi: 10.1021/acs.jnatprod.2c00924 (PMC9972480; doi:10.1021/acs.jnatprod.2c00924)

Supporting Information

Synthesis of quinoline and dihydroquinoline embelin derivatives as cardioprotective agents

Pedro Martín-Acosta,[a,†] Irene Cuadrado,[b,†] Laura González-Cofrade,[b] Roberto Pestano,[a] Sonsoles Hortelano,[c,*] Beatriz de las Heras,[b,*] Ana Estévez-Braun[a,*]

[a]*Instituto Universitario de Bio-Orgánica Antonio González, Departamento de Química Orgánica, Universidad de La Laguna, Avda. Astrofísico Francisco Sánchez Nº 2, 38206, La Laguna, Tenerife, Spain*

[b]*Departamento de Farmacología, Farmacognosia y Botánica, Facultad de Farmacia, Universidad Complutense de Madrid (UCM), Plaza Ramón y Cajal s/n-28040, Madrid, Spain*

[c]*Unidad de Terapias Farmacológicas, Área de Genética Humana, Instituto de Investigación de Enfermedades Raras (IIER), Instituto de Salud Carlos III, Carretera de Majadahonda-Pozuelo Km 2-28220, Madrid, Spain*

[†] *These authors have contributed equally to this work*

Contents **Page**

Figure S1. ^{1}H-NMR (DMSO-d_6, 500 MHz) of compound **4a** **S4**
Figure S2. ^{13}C-NMR ($CDCl_3$, 150 MHz) of compound **4a** **S4**
Figure S3. IR spectrum (film) of compound **4a** **S5**
Figure S4. UV spectrum (EtOH) of compound **4a** **S5**
Figure S5. ^{1}H-NMR ($CDCl_3$, 500 MHz) of compound **5a** **S6**
Figure S6. ^{13}C-NMR ($CDCl_3$, 150 MHz) of compound **5a** **S6**
Figure S7. IR spectrum (film) of compound **5a** **S7**
Figure S8. ^{1}H-NMR ($CDCl_3$, 500 MHz) of compound **4b** **S8**
Figure S9. ^{13}C-NMR ($CDCl_3$, 150 MHz) of compound **4b** **S8**
Figure S10. IR spectrum (film) of compound **4b** **S9**
Figure S11. UV spectrum (EtOH) of compound **4b** **S9**
Figure S12. ^{1}H-NMR ($CDCl_3$, 500 MHz) of compound **4c** **S10**

Figure S13. ^{13}C-NMR ($CDCl_3$, 125 MHz) of compound **4c** **S10**
Figure S14. IR spectrum (film) of compound **4c** **S11**
Figure S15. UV spectrum (EtOH) of compound **4c** **S11**
Figure S16. ^{1}H-NMR ($CDCl_3$, 500 MHz) of compound **4d** **S12**
Figure S17. ^{13}C-NMR ($CDCl_3$, 125 MHz) of compound **4d** **S12**
Figure S18. IR spectrum (film) of compound **4d** **S13**
Figure S19. UV spectrum (EtOH) of compound **4d** **S13**
Figure S20. ^{1}H-NMR ($CDCl_3$, 500 MHz) of compound **4e** **S14**
Figure S21. ^{13}C-NMR ($CDCl_3$, 125 MHz) of compound **4e** **S14**
Figure S22. IR spectrum (film) of compound **4e** **S15**
Figure S23. UV spectrum (EtOH) of compound **4e** **S15**
Figure S24. ^{1}H-NMR ($CDCl_3$, 500 MHz) of compound **4f** **S16**
Figure S25. ^{13}C-NMR ($CDCl_3$, 125 MHz) of compound **4f** **S16**
Figure S26. IR spectrum (film) of compound **4f** **S17**
Figure S27. UV spectrum (EtOH) of compound **4f** **S17**
Figure S28. ^{1}H-NMR ($CDCl_3$, 500 MHz) of compound **4g** **S18**
Figure S29. ^{13}C-NMR ($CDCl_3$, 125 MHz) of compound **4g** **S18**
Figure S30. IR spectrum (film) of compound **4g** **S19**
Figure S31. UV spectrum (EtOH) of compound **4g** **S19**
Figure S32. ^{1}H-NMR ($CDCl_3$, 500 MHz) of compound **4h** **S20**
Figure S33. ^{13}C-NMR ($CDCl_3$, 125 MHz) of compound **4h** **S20**
Figure S34. IR spectrum (film) of compound **4h** **S21**
Figure S35. UV spectrum (EtOH) of compound **4h** **S21**
Figure S36. ^{1}H-NMR ($CDCl_3$, 500 MHz) of compound **4i** **S22**
Figure S37. ^{13}C-NMR ($CDCl_3$, 125 MHz) of compound **4i** **S22**
Figure S38. IR spectrum (film) of compound **4i** **S23**
Figure S39. UV spectrum (EtOH) of compound **4i** **S23**
Figure S40. ^{1}H-NMR ($CDCl_3$, 500 MHz) of compound **4j** **S24**
Figure S41. ^{13}C-NMR ($CDCl_3$, 150 MHz) of compound **4j** **S24**
Figure S42. IR spectrum (film) of compound **4j** **S25**
Figure S43. UV spectrum (EtOH) compound **4j** **S25**
Figure S44. ^{1}H-NMR ($CDCl_3$, 500 MHz) of compound **4k** **S26**
Figure S46. ^{13}C-NMR ($CDCl_3$, 125 MHz) of compound **4k** **S26**
Figure S47. IR spectrum (film) of compound **4k** **S27**
Figure S48. UV spectrum (EtOH) of compound **4k** **S27**
Figure S49. ^{1}H-NMR ($CDCl_3$, 500 MHz) of compound **5b** **S28**
Figure S50. ^{13}C-NMR (125 MHz, $CDCl_3$) of compound **5b** **S28**
Figure S51.^{1}H-NMR ($CDCl_3$, 500 MHz) of compound **4l** **S29**
Figure S52.^{13}C-NMR ($CDCl_3$, 125 MHz) of compound **4l** **S29**
Figure S53. IR spectrum (film) of compound **4l** **S30**
Figure S54. UV spectrum (EtOH) of compound **4l** **S30**
Figure S55. ^{1}H-NMR ($CDCl_3$, 500 MHz) of compound **4m** **S31**
Figure S56. ^{13}C-NMR ($CDCl_3$, 125 MHz) of compound **4m** **S31**
Figure S57. IR spectrum (film) of compound **4m** **S32**
Figure S58. UV spectrum (EtOH) of compound **4m** **S32**
Figure S59. ^{1}H-NMR ($CDCl_3$, 500 MHz) of compound **5c** **S33**
Figure S60. ^{13}C-NMR ($CDCl_3$, 125 MHz) of compound **5c** **S33**
Figure S61. ^{1}H-NMR ($CDCl_3$, 500 MHz) of compound **5d** **S34**
Figure S62. ^{13}C-NMR ($CDCl_3$, 150 MHz) of compound **5d** **S34**
Figure S63. ^{1}H-NMR ($CDCl_3$, 500 MHz) of compound **6a** **S35**
Figure S64. ^{13}C-NMR ($CDCl_3$, 150 MHz) of compound **6a** **S35**
Figure S65. IR spectrum (film) of compound **6a** **S36**

Figure S66. UV spectrum (EtOH) of compound **6a** S36
Figure S67. ^{1}H-NMR ($CDCl_3$, 500 MHz) of compound **6b** S37
Figure S68. ^{13}C-NMR ($CDCl_3$, 150 MHz) of compound **6b** S37
Figure S69. IR spectrum (film) of compound **6b** S38
Figure S70. UV spectrum (EtOH) of compound **6b** S38
Figure S71. ^{1}H-NMR ($CDCl_3$, 500 MHz) of compound **6c** S39
Figure S72. ^{13}C-NMR ($CDCl_3$, 150 MHz) of compound **6c** S39
Figure S73. IR spectrum (film) of compound **6c** S40
Figure S74. UV spectrum (EtOH) of compound **6c** S40
Figure S75. ^{1}H-NMR ($CDCl_3$, 500 MHz) of compound **6d** S41
Figure S76. ^{13}C-NMR ($CDCl_3$, 150 MHz) of compound **6d** S41
Figure S77. IR spectrum (film) of compound **6d** S42
Figure S78. UV spectrum (EtOH) of compound **6d** S42
Figure S79. ^{1}H-NMR ($CDCl_3$, 500 MHz) of compound **6e** S43
Figure S80. ^{13}C-NMR ($CDCl_3$, 150 MHz) of compound **6e** S43
Figure S81. IR spectrum (film) of compound **6e** S44
Figure S82. UV spectrum (EtOH) of compound **6e** S44
Figure S83. ^{1}H-NMR ($CDCl_3$, 500 MHz) of compound **6f** S45
Figure S84. ^{13}C-NMR ($CDCl_3$, 125 MHz) of compound **6f** S45
Figure S85. IR spectrum (film) of compound **6f** S46
Figure S86. UV spectrum (EtOH) of compound **6f** S46
Figure S87. ^{1}H-NMR ($CDCl_3$, 500 MHz) of compound **6g** S47
Figure S88. ^{13}C-NMR ($CDCl_3$, 150 MHz) of compound **6g** S47
Figure S89. IR spectrum (film) of compound **6g** S48
Figure S90. UV spectrum (EtOH) of compound **6g** S48
Figure S91. ^{1}H-NMR ($CDCl_3$, 500 MHz) of compound **6h** S49
Figure S92. ^{13}C-NMR ($CDCl_3$, 125 MHz) of compound **6h** S49
Figure S93. IR spectrum (film) of compound **6h** S50
Figure S94. UV spectrum (EtOH) of compound **6h** S50
Figure S95. ^{1}H-NMR ($CDCl_3$, 500 MHz) of compound **6i** S51
Figure S96. ^{13}C-NMR ($CDCl_3$, 125 MHz) of compound **6i** S51
Figure S97. IR spectrum (film) of compound **6i** S52
Figure S98. UV spectrum (EtOH) of compound **6i** S52
Figure S99. ^{1}H-NMR ($CDCl_3$, 500 MHz) of compound **6j** S53
Figure S100. ^{13}C-NMR ($CDCl_3$, 125 MHz) of compound **6j** S53
Figure S101. IR spectrum (film) of compound **6j** S54
Figure S102. UV spectrum (EtOH) of compound **6j** S54
Figure S103. ^{1}H-NMR ($CDCl_3$, 500 MHz) of compound **6k** S55
Figure S104. ^{13}C-NMR ($CDCl_3$, 125 MHz) of compound **6k** S55
Figure S105. IR spectrum (film) of compound **6k** S56
Figure S106. UV spectrum (EtOH) of compound **6k** S56
Figure S107. ^{1}H-NMR ($CDCl_3$, 500 MHz) of compound **6l** S57
Figure S108. ^{13}C-NMR ($CDCl_3$, 125 MHz) of compound **6l** S57
Figure S109. IR spectrum (film) of compound **6l** S58
Figure S110. UV spectrum (EtOH) of compound **6l** S58
Figure S111. ^{1}H-NMR ($CDCl_3$, 500 MHz) of compound **6m** S59
Figure S112. ^{13}C-NMR ($CDCl_3$, 125 MHz) of compound **6m** S59
Figure S113. IR spectrum (film) of compound **6m** S60
Figure S114. UV spectrum (EtOH) of compound **6m** S60

Figure S1. ^{1}H-NMR (DMSO-d_6, 500 MHz) spectrum of compound **4a**

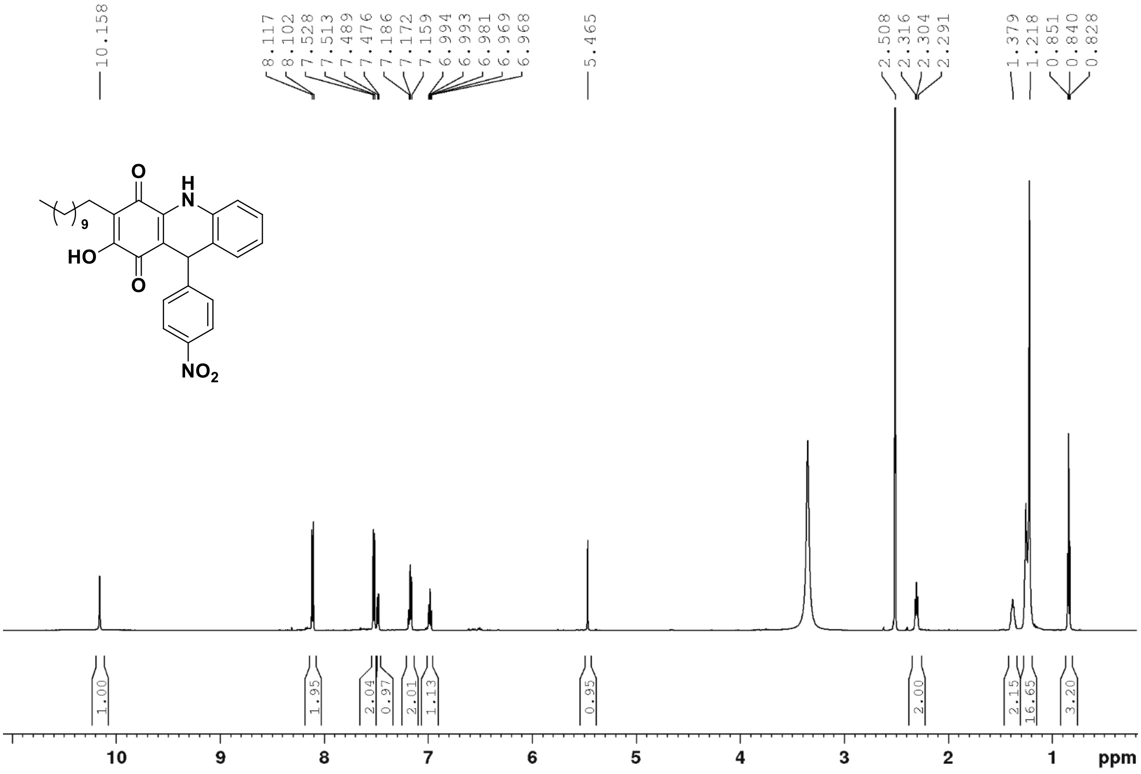

Figure S2. ^{13}C-NMR ($CDCl_3$, 150 MHz) spectrum of compound **4a**

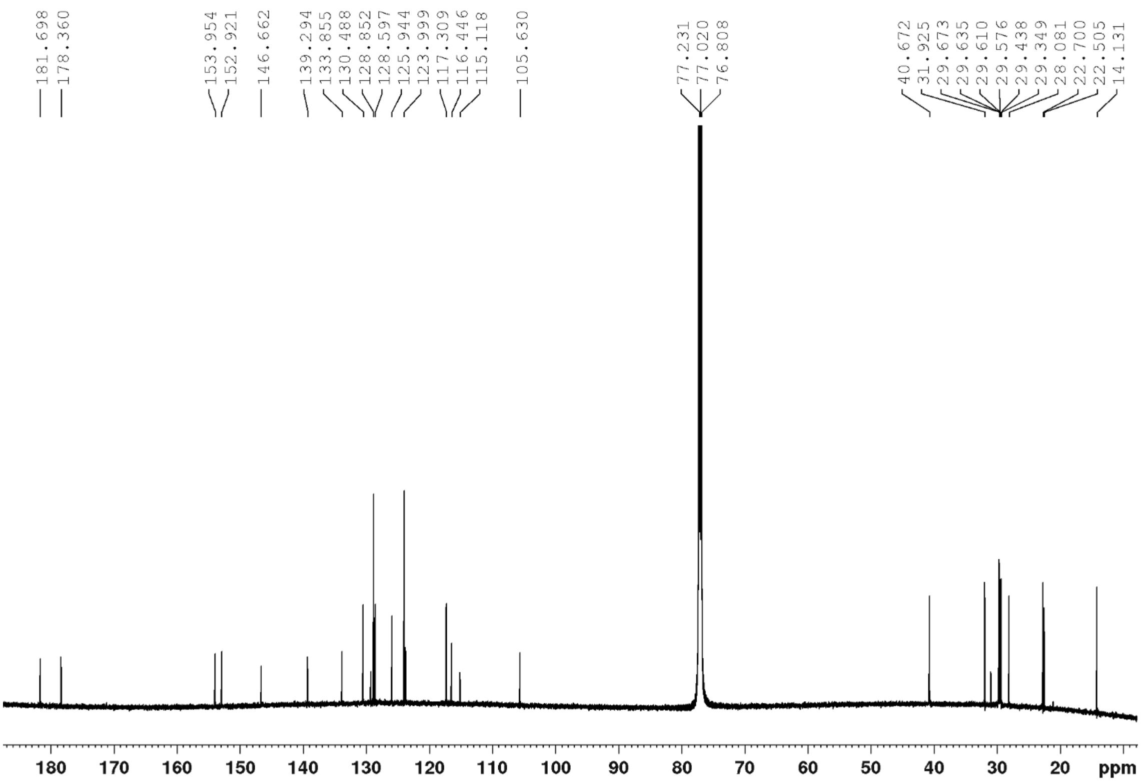

Figure S3. IR spectrum (film) of compound **4a**

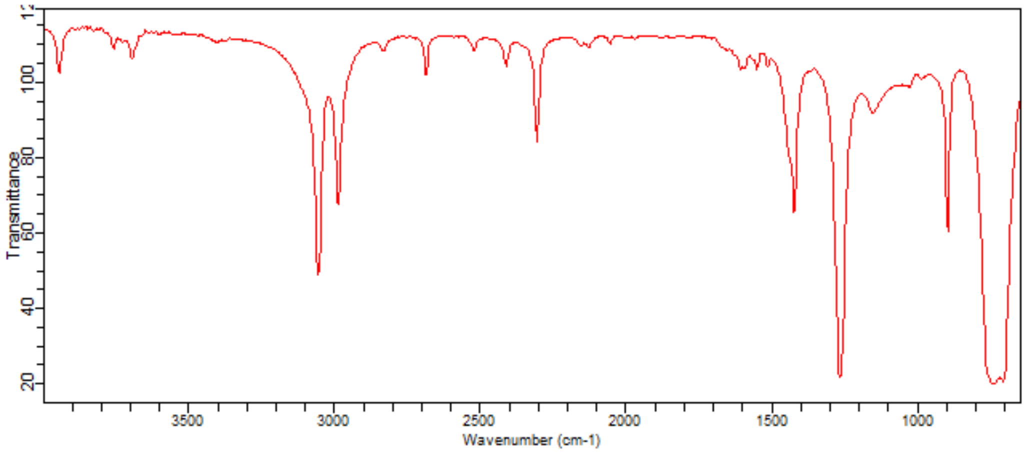

Figure S4. UV spectrum (EtOH) of compound **4a**

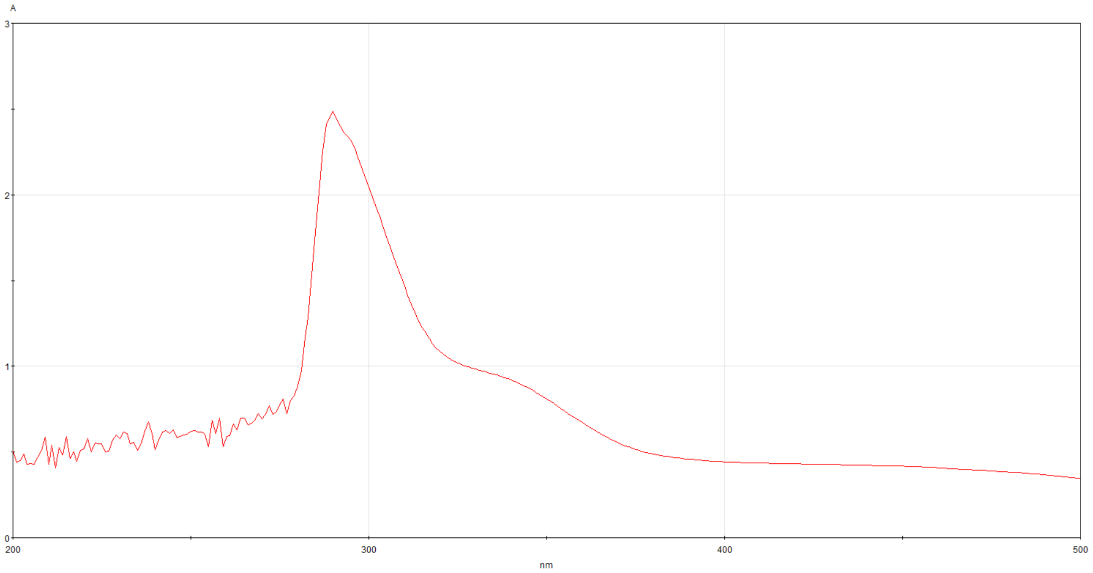

Figure S5. ^{1}H-NMR ($CDCl_3$, 500 MHz) spectrum of compound **5a**

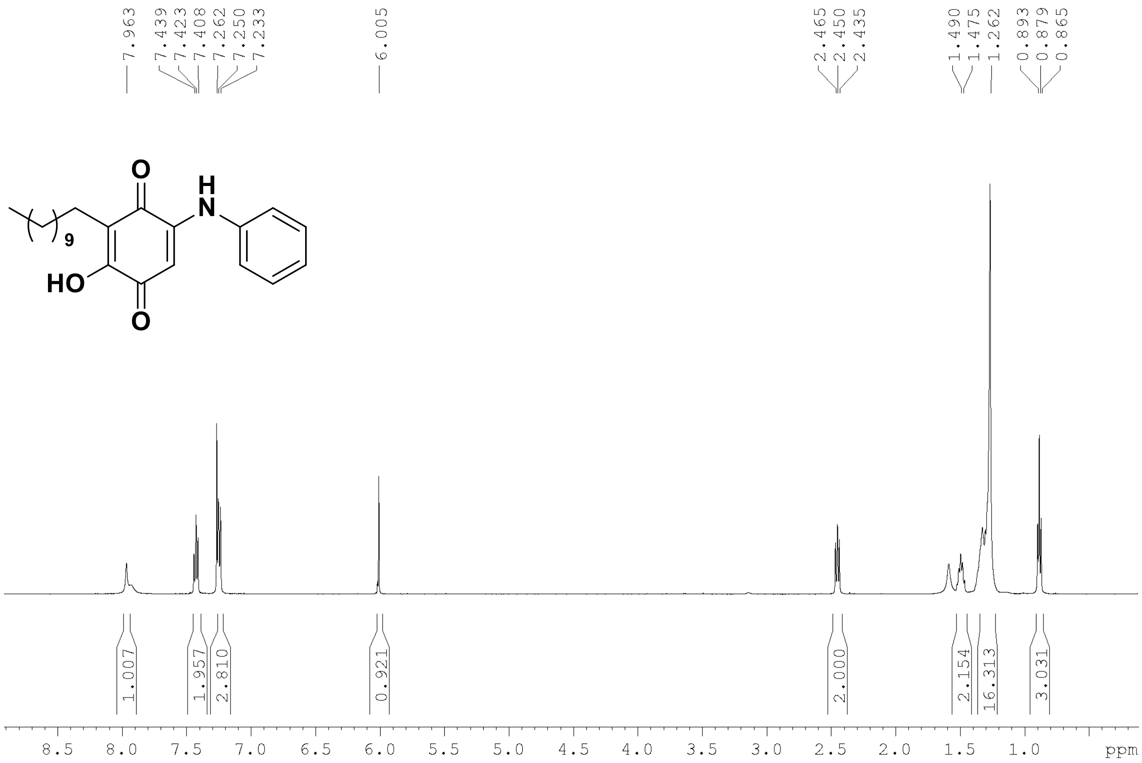

Figure S6. ^{13}C-NMR ($CDCl_3$, 150 MHz) spectrum of compound **5a**

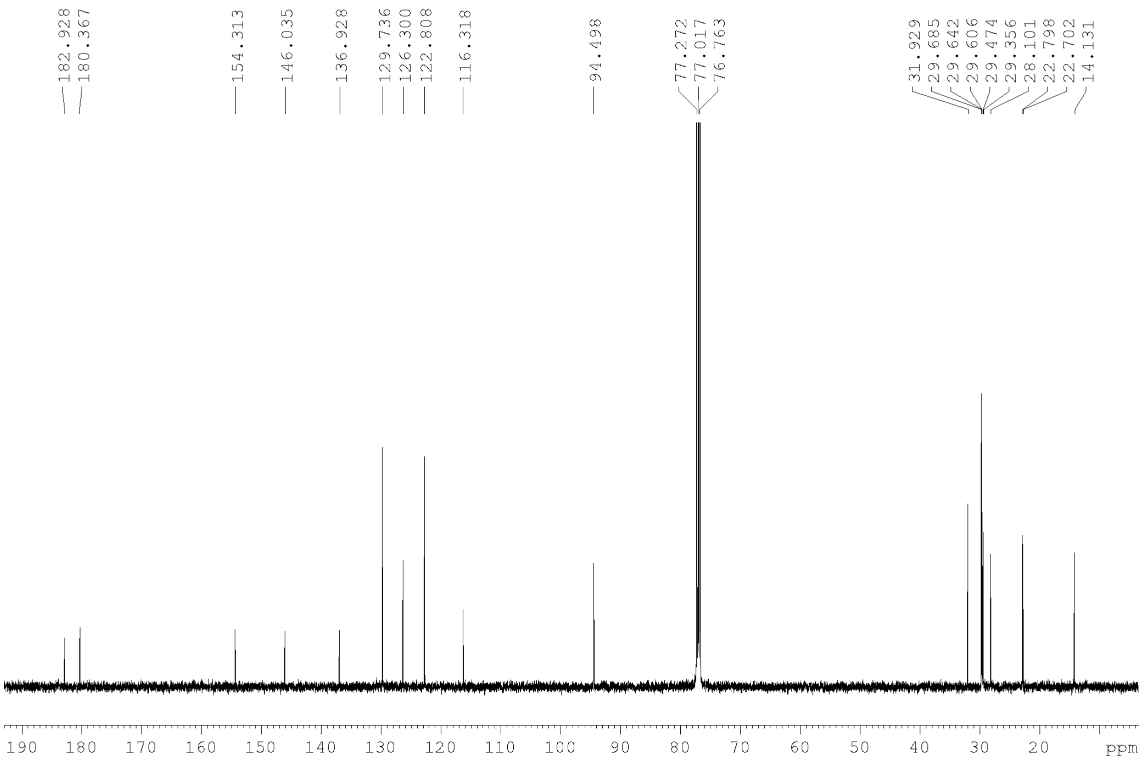

Figure S7. IR spectrum (film) of compound **5a**

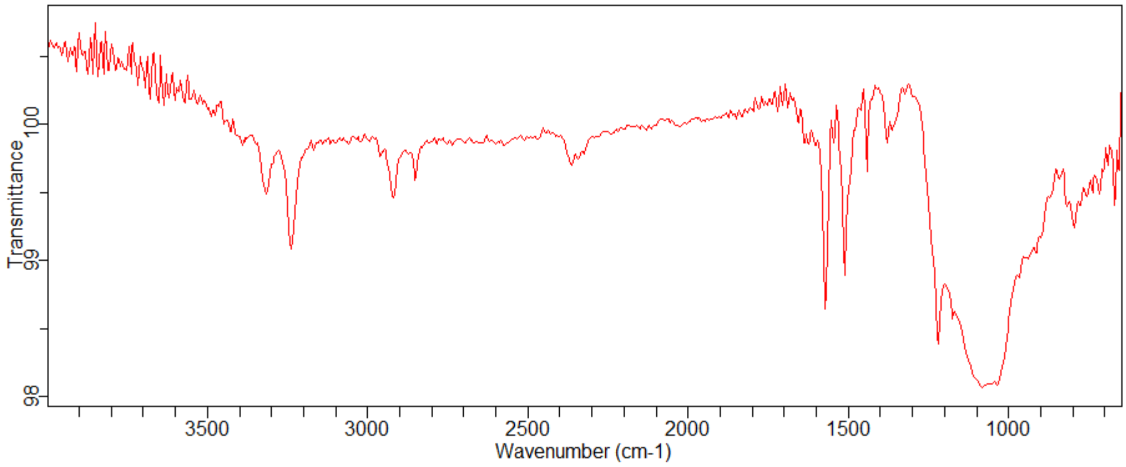

Figure S8. ^{1}H-NMR ($CDCl_3$, 500 MHz) spectrum of compound **4b**

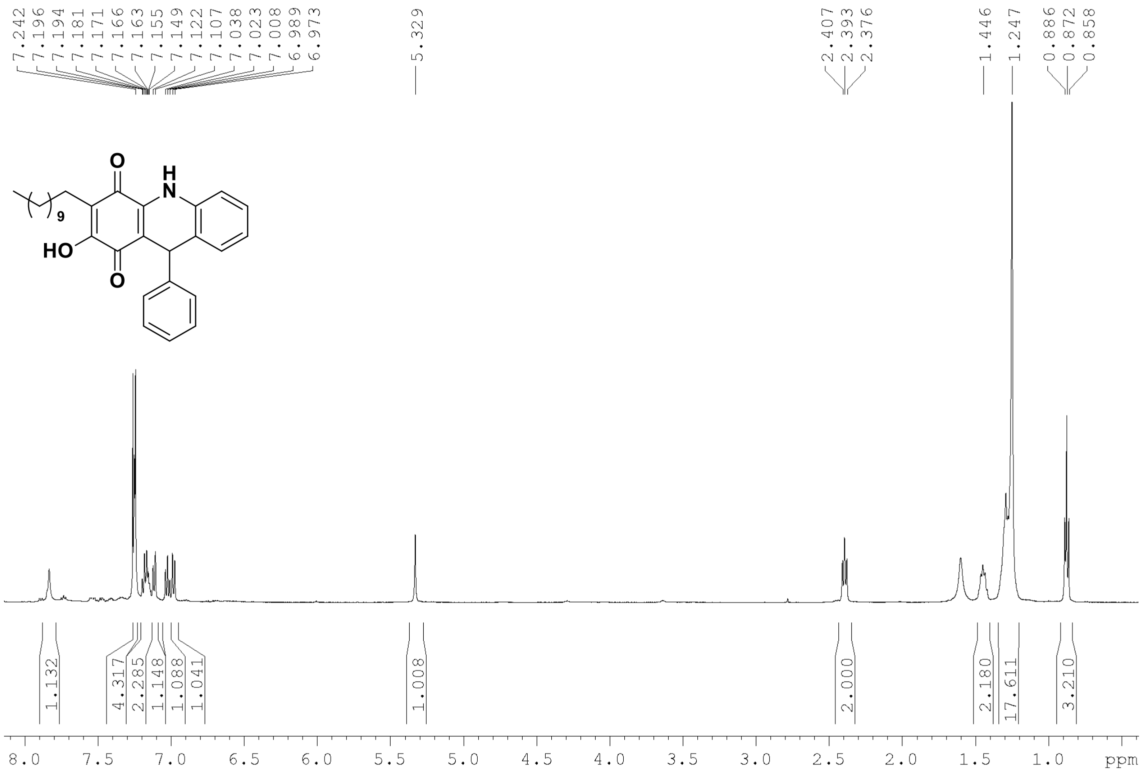

Figure S9. ^{13}C-NMR ($CDCl_3$, 150 MHz) spectrum of compound **4b**

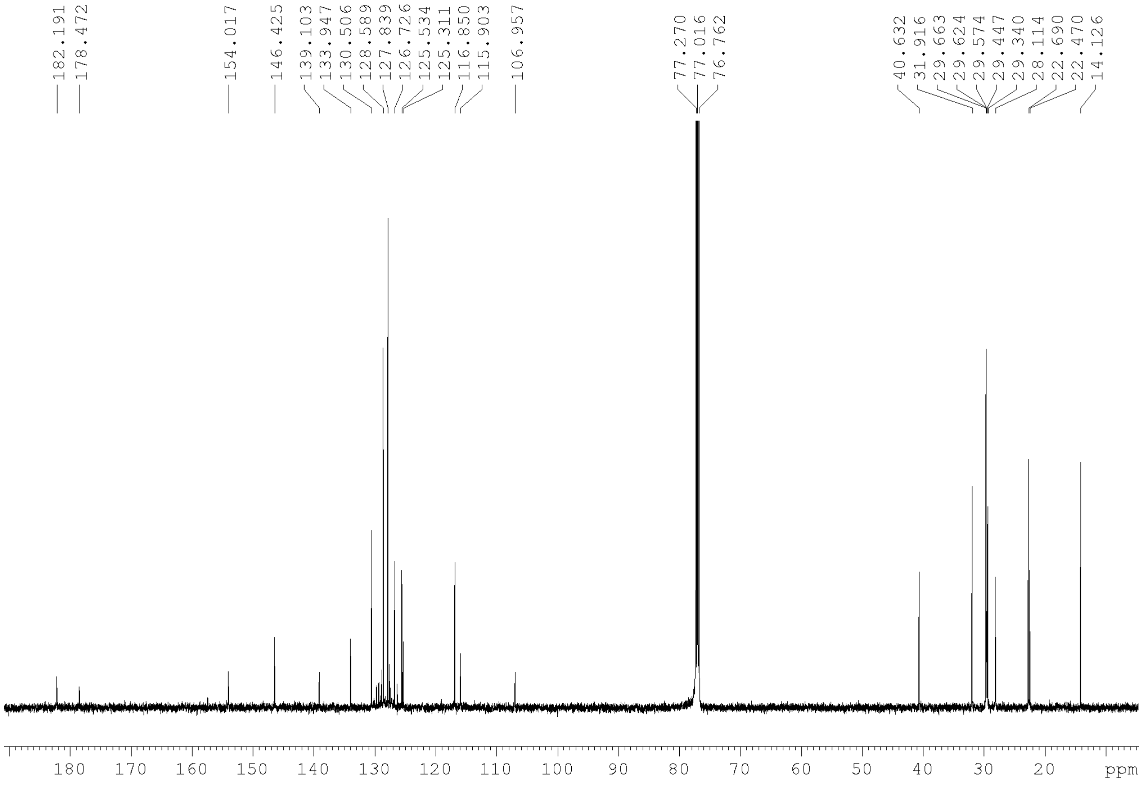

Figure S10. IR spectrum (film) of compound **4b**

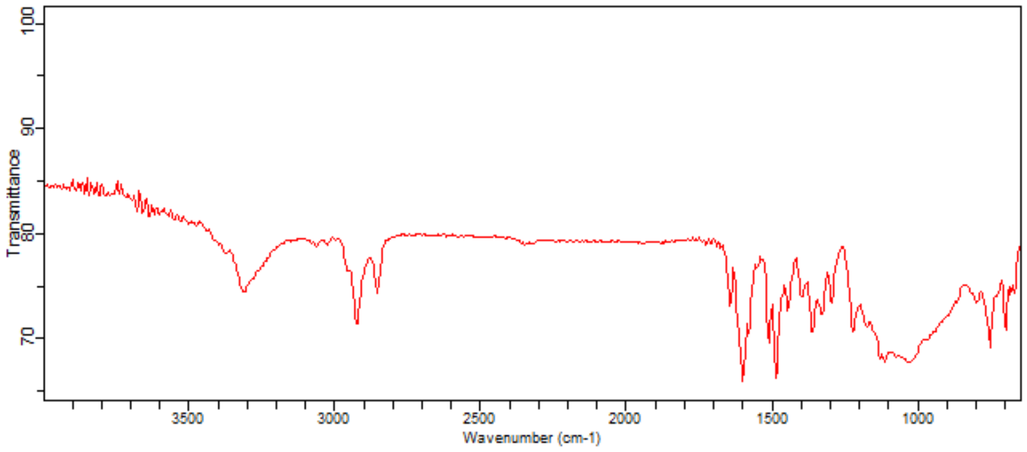

Figure S11. UV spectrum (EtOH) of compound **4b**

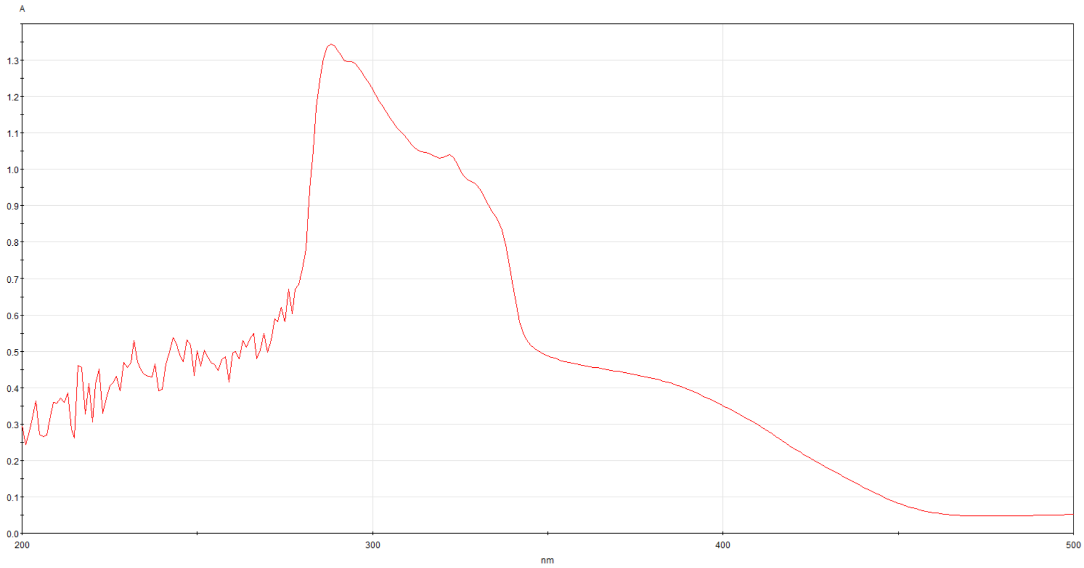

Figure S12. ^{1}H-NMR ($CDCl_3$, 500 MHz) spectrum of compound **4c**

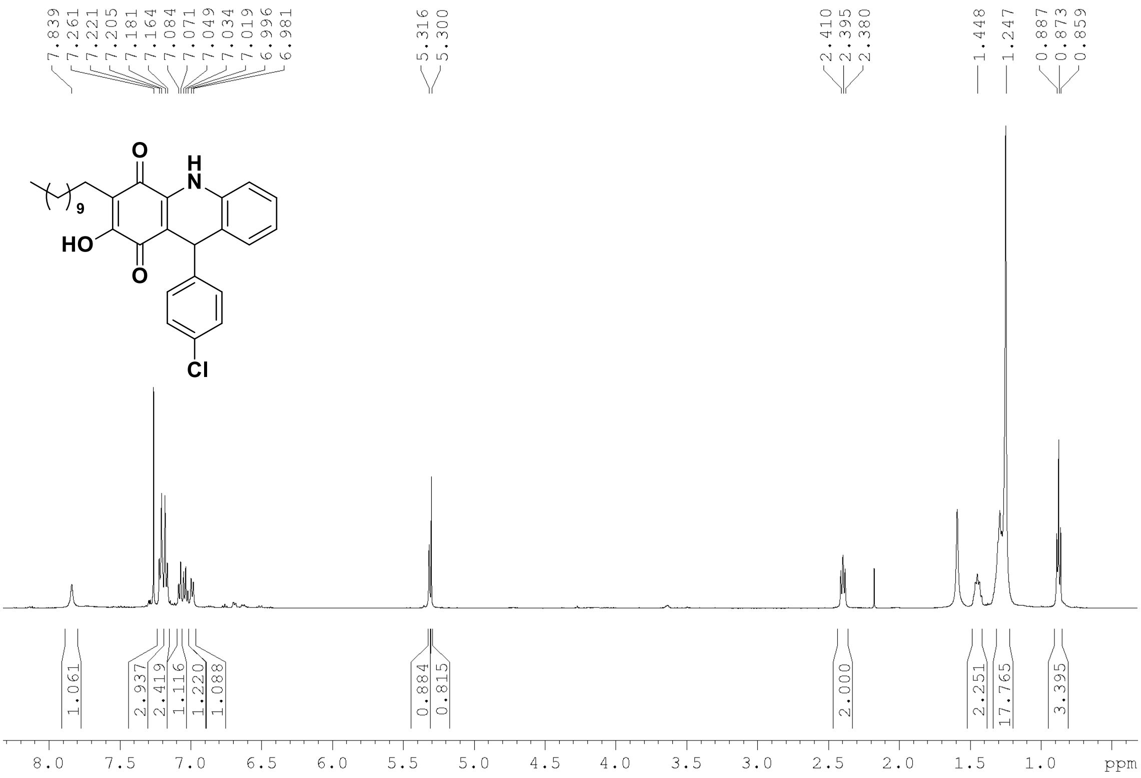

Figure S13. ^{13}C-NMR ($CDCl_3$, 125 MHz) spectrum of compound **4c**

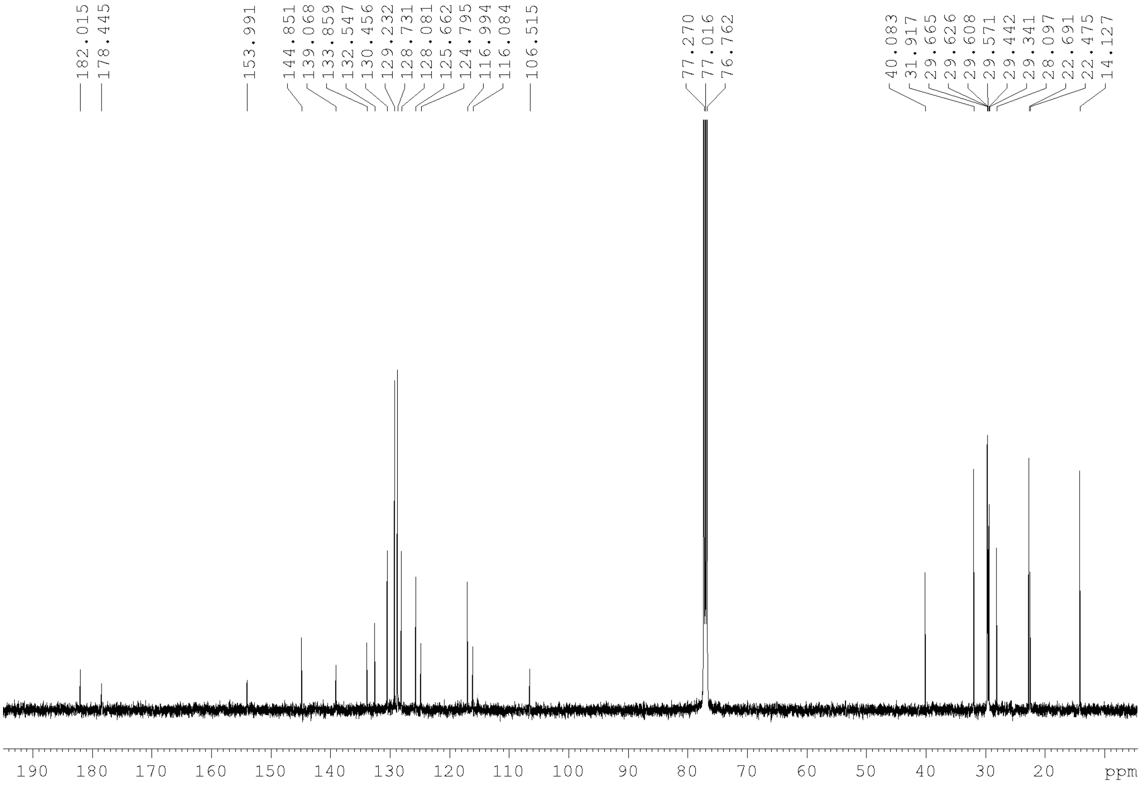

Figure S14. IR spectrum (film) spectrum of compound **4c**

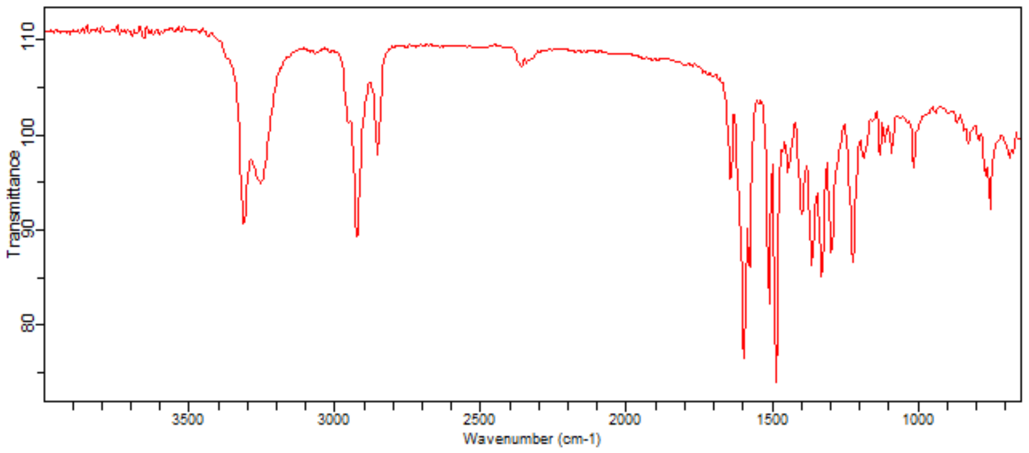

Figure S15. UV spectrum (EtOH) of compound **4c**

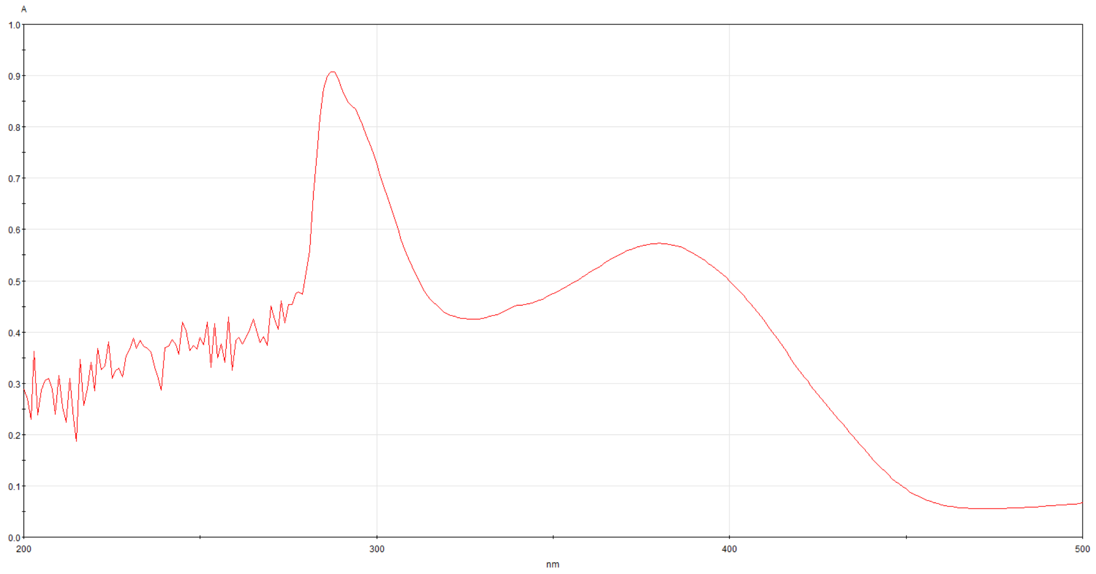

Figure S16. ^{1}H-NMR (DMSO-d_6, 500 MHz) spectrum of compound **4d**

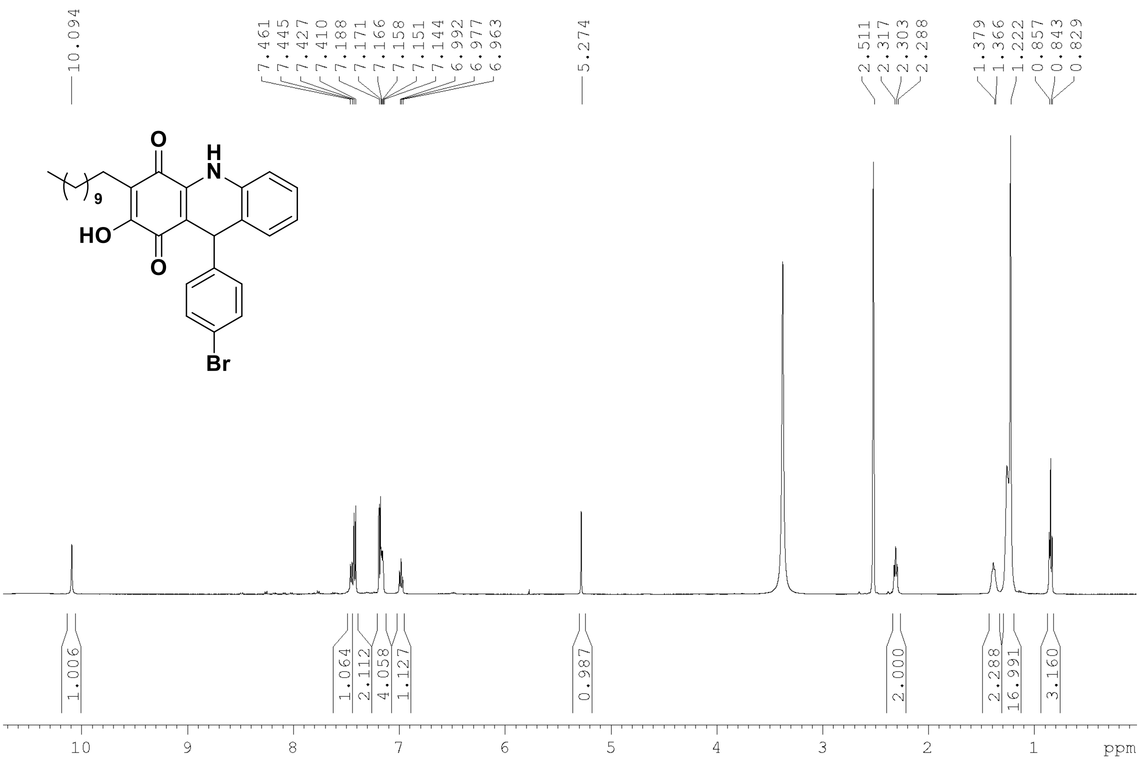

Figure S17. ^{13}C-NMR (DMSO-d_6, 125 MHz) spectrum of compound **4d**

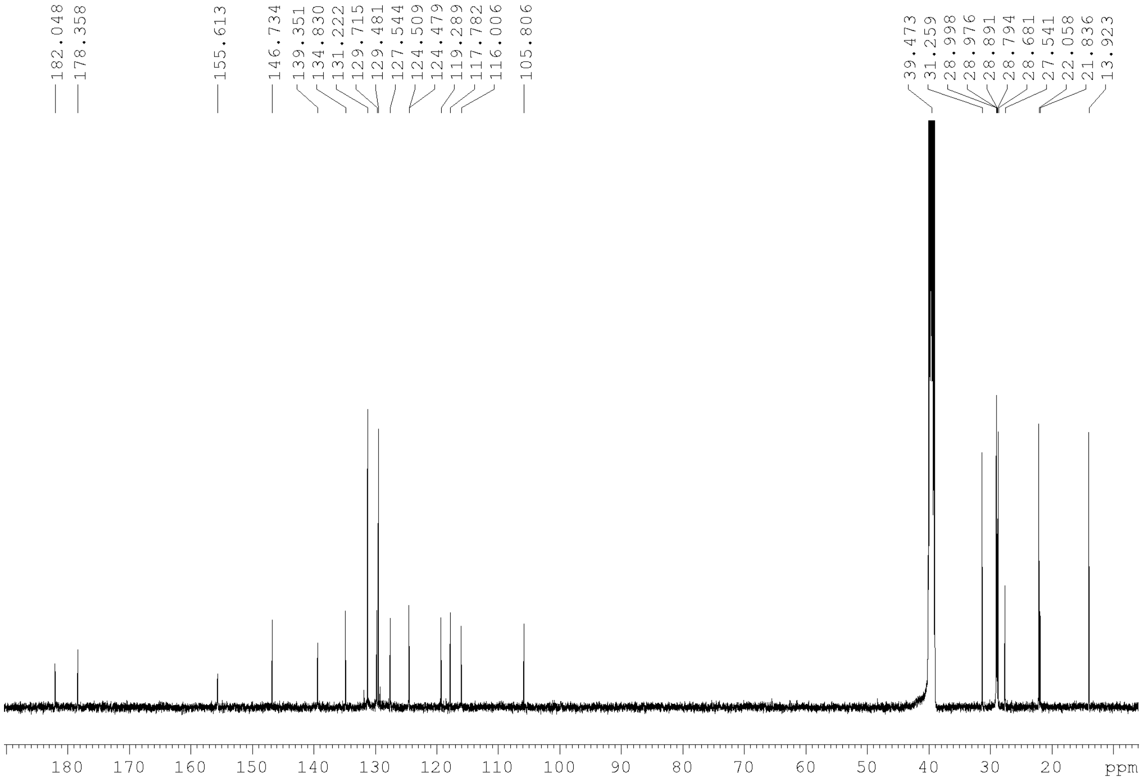

Figure S18. IR spectrum (film) of compound **4d**

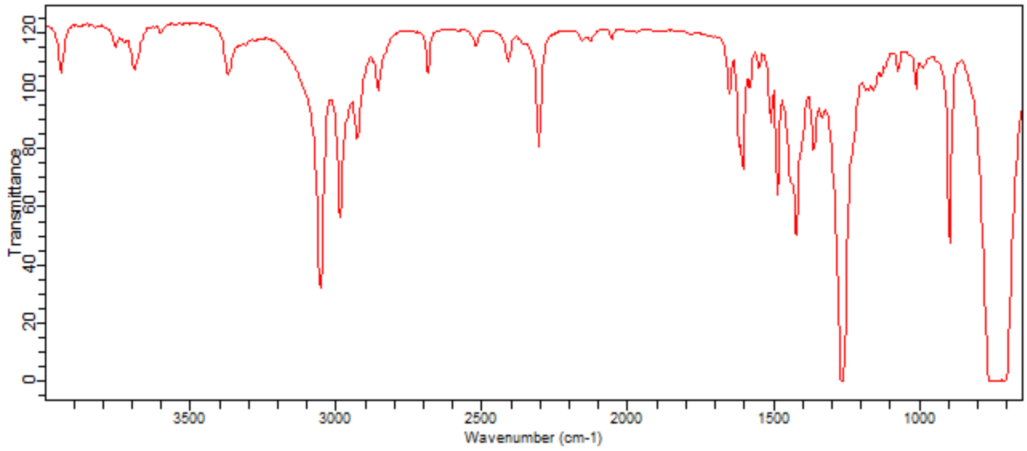

Figure S19. UV spectrum (EtOH) of compound **4d**

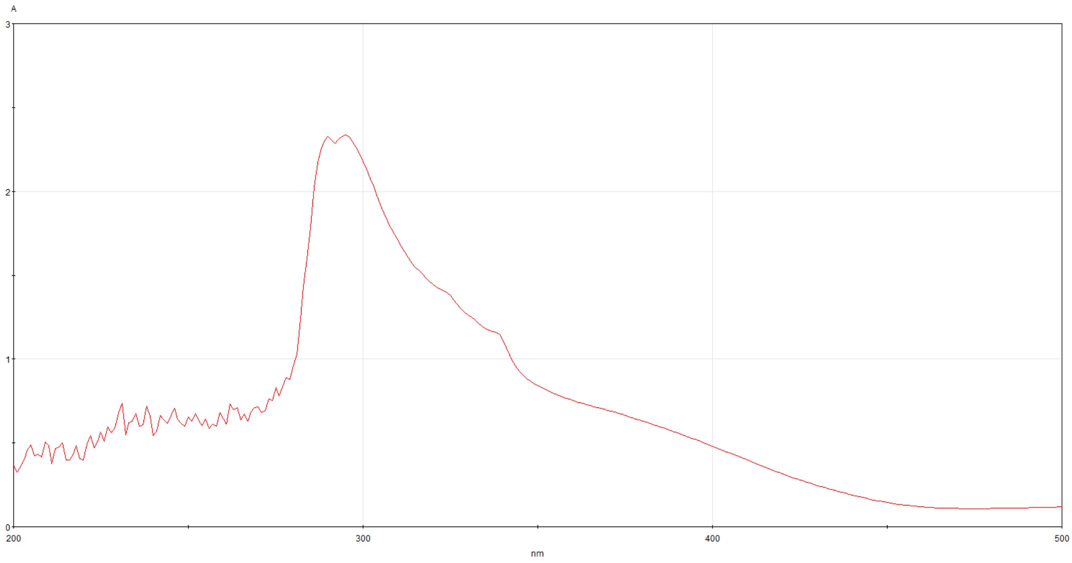

Figure S20. ^{1}H-NMR ($CDCl_3$, 500 MHz) spectrum of compound **4e**

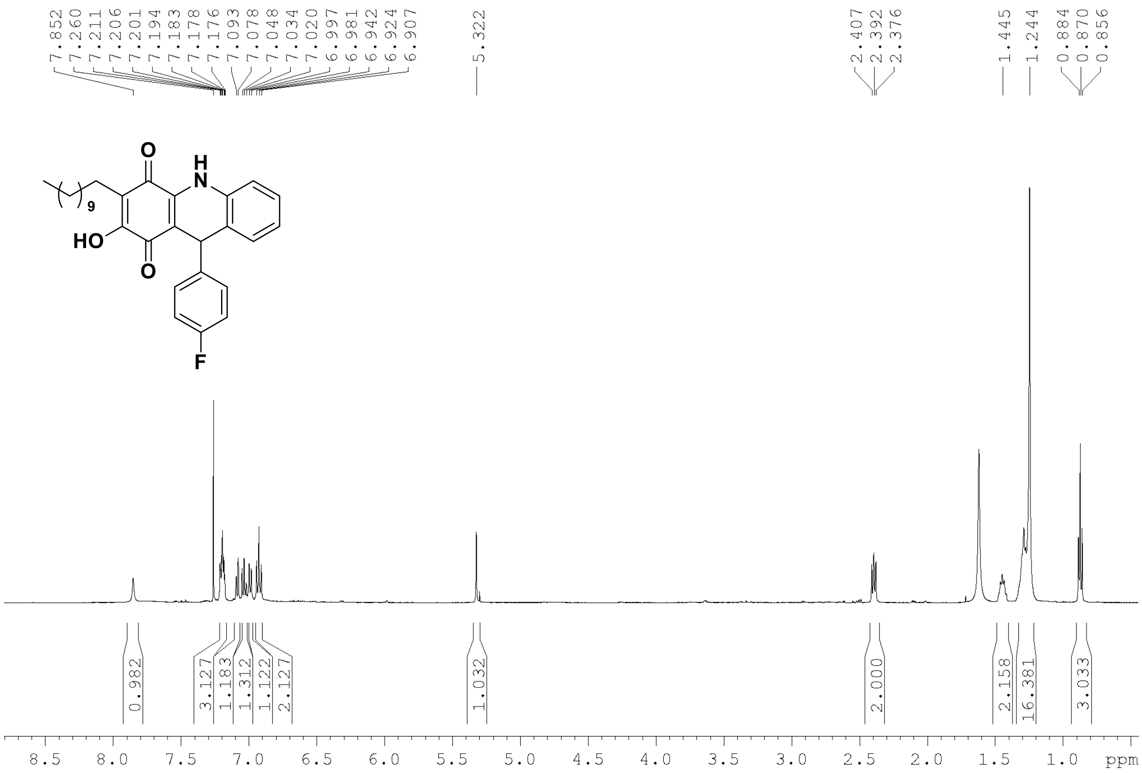

Figure S21. ^{13}C-NMR ($CDCl_3$, 125 MHz) spectrum of compound **4e**

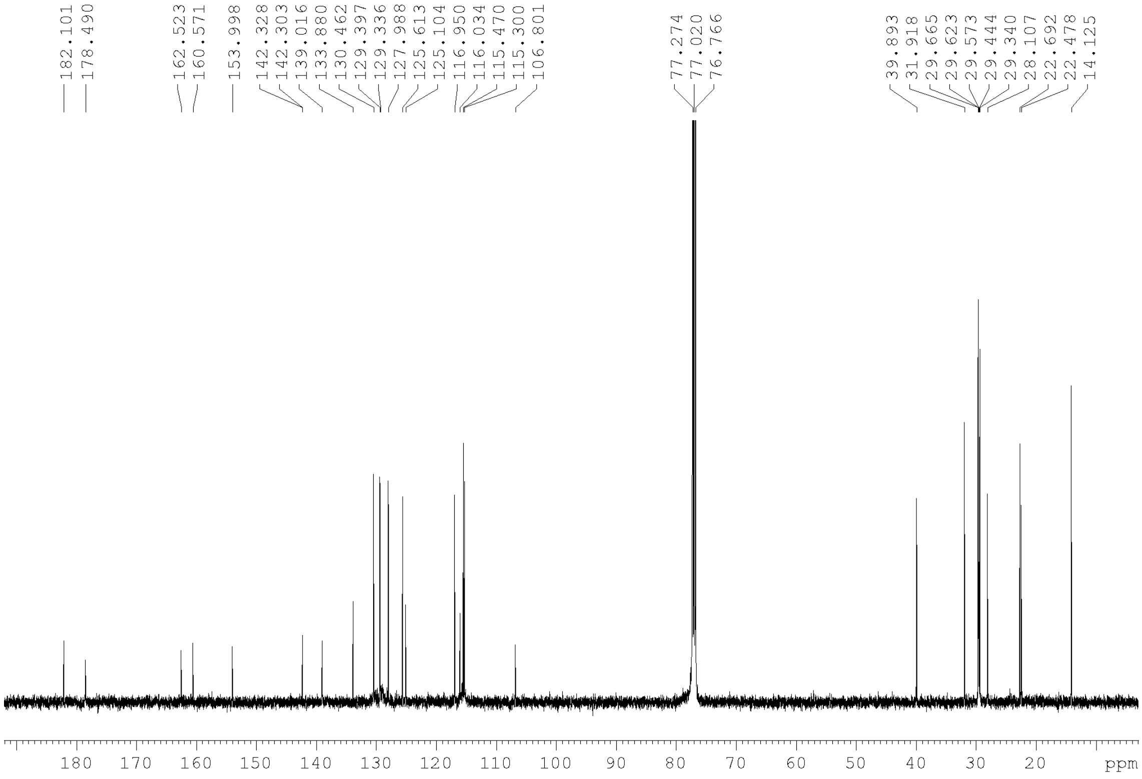

Figure S22. IR spectrum (film) of compound **4e**

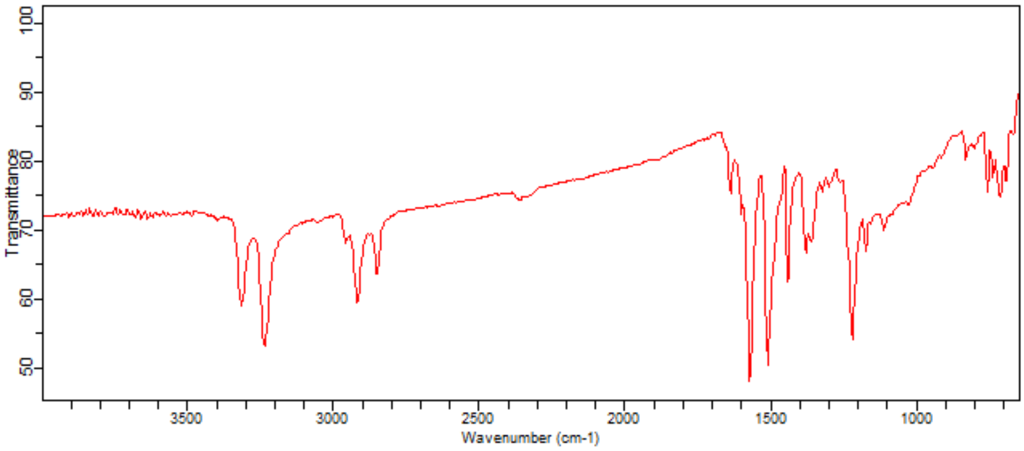

Figure S23. UV spectrum (EtOH) of compound **4e**

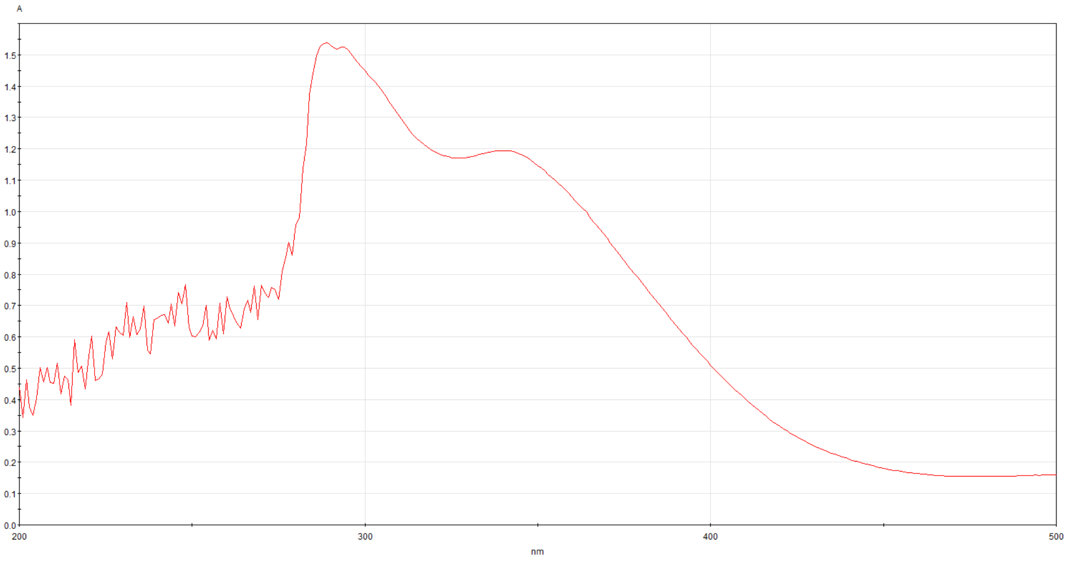

Figure S24. ^{1}H-NMR ($CDCl_3$, 500 MHz) spectrum of compound **4f**

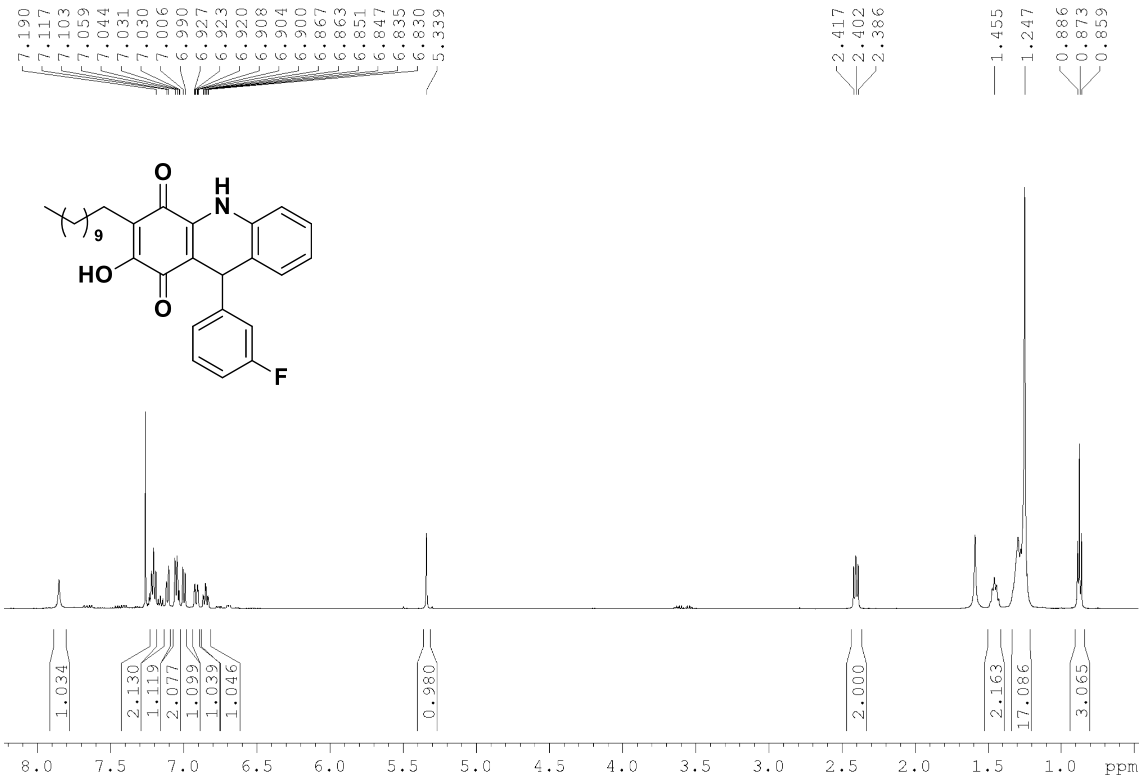

Figure S25. ^{13}C-NMR ($CDCl_3$, 125 MHz) spectrum of compound **4f**

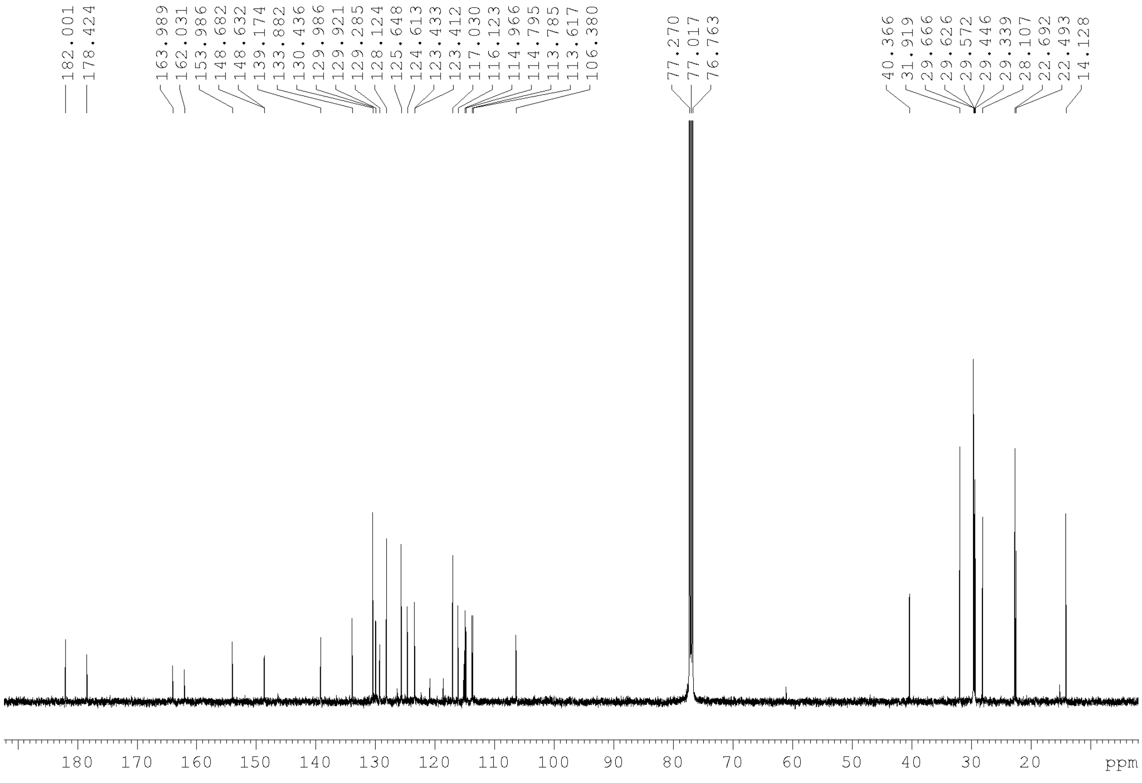

Figure S26. IR spectrum (film) of compound **4f**

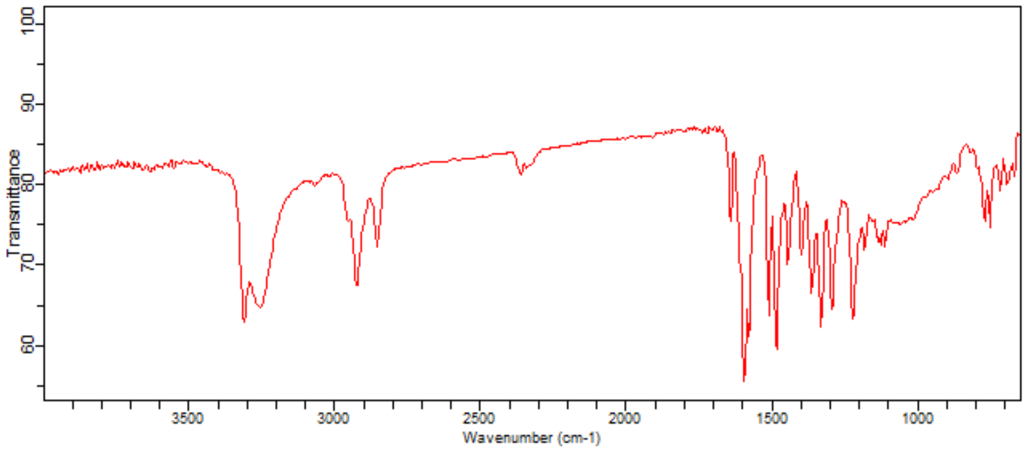

Figure S27. UV spectrum (EtOH) of compound **4f**

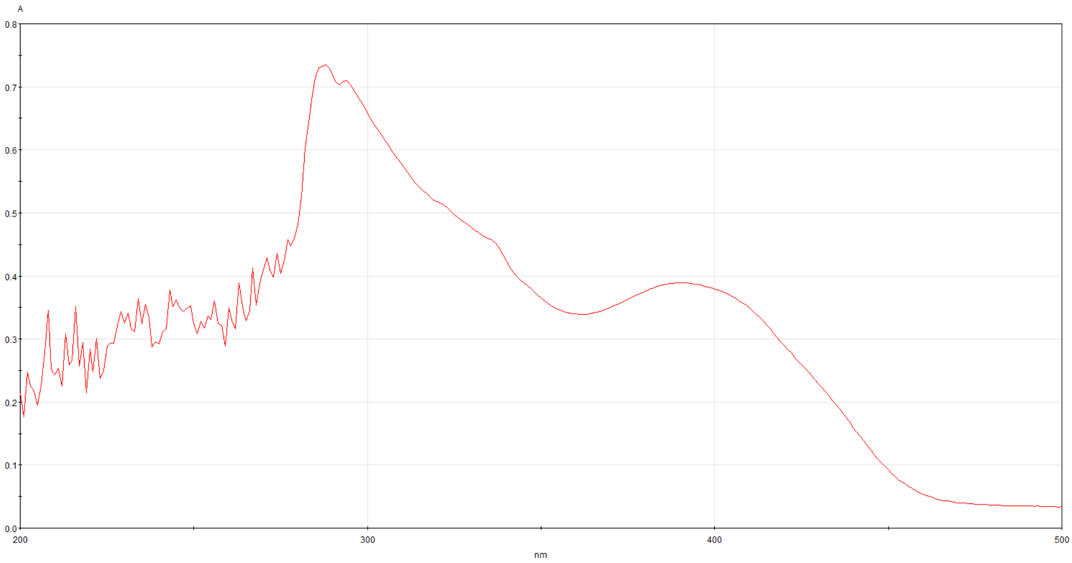

Figure S28. ^{1}H-NMR ($CDCl_3$, 500 MHz) spectrum of compound **4g**

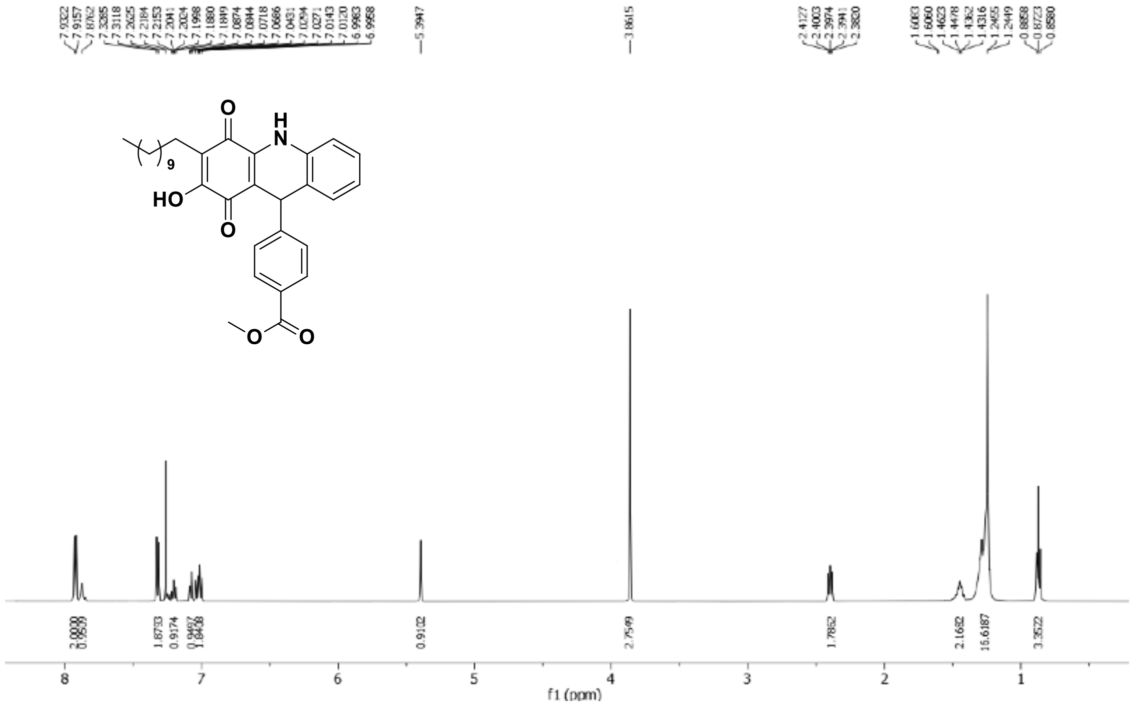

Figure S29. ^{13}C-NMR ($CDCl_3$, 125 MHz) spectrum of compound **4g**

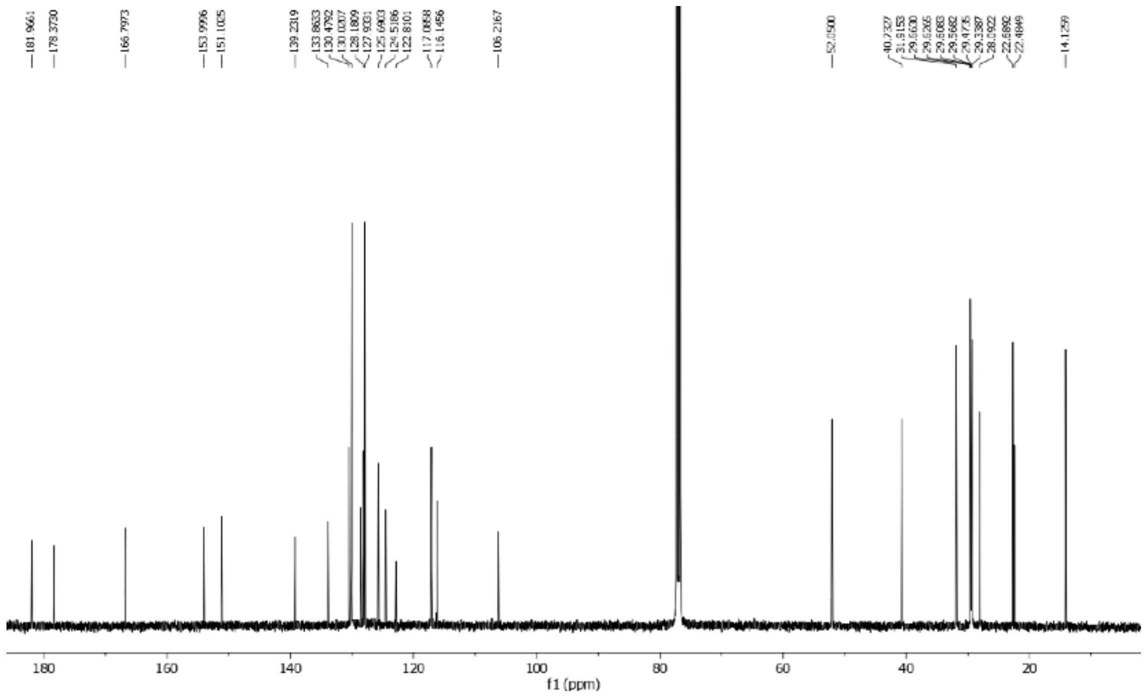

Figure S30. IR spectrum (film) of compound **4g**

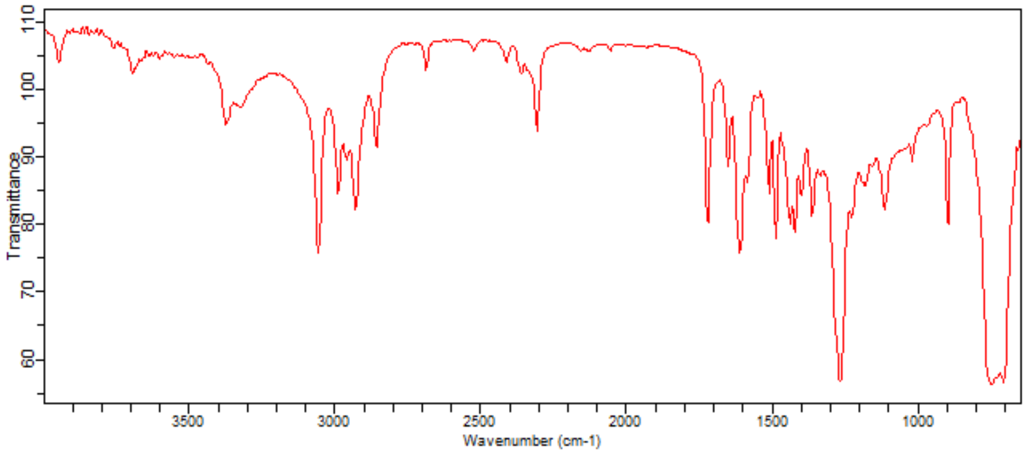

Figure S31. UV spectrum (EtOH) of compound **4g**

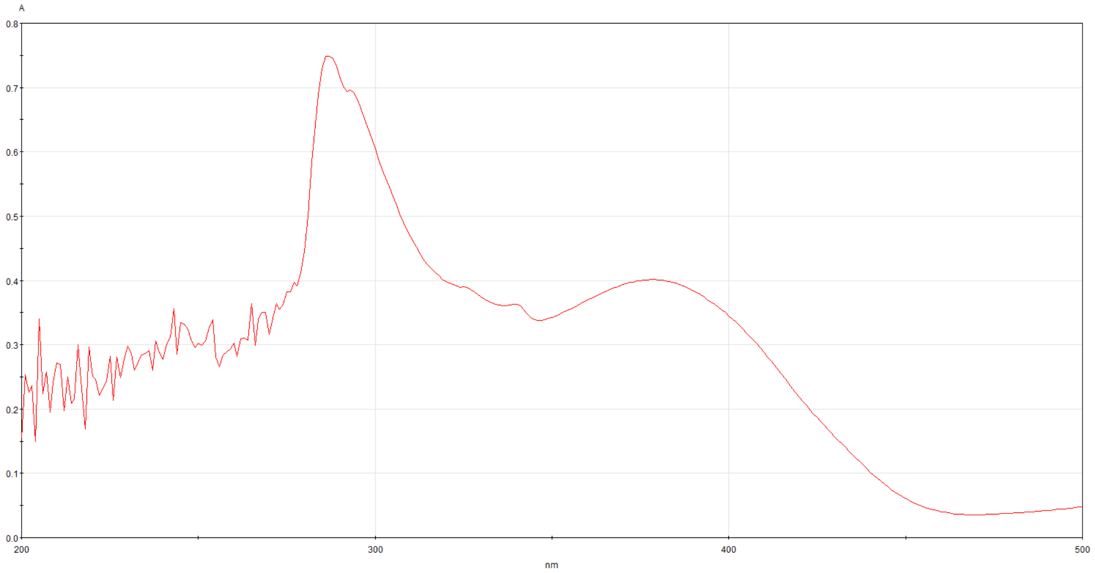

Figure S32. ^{1}H-NMR ($CDCl_3$, 500 MHz) spectrum of compound **4h**

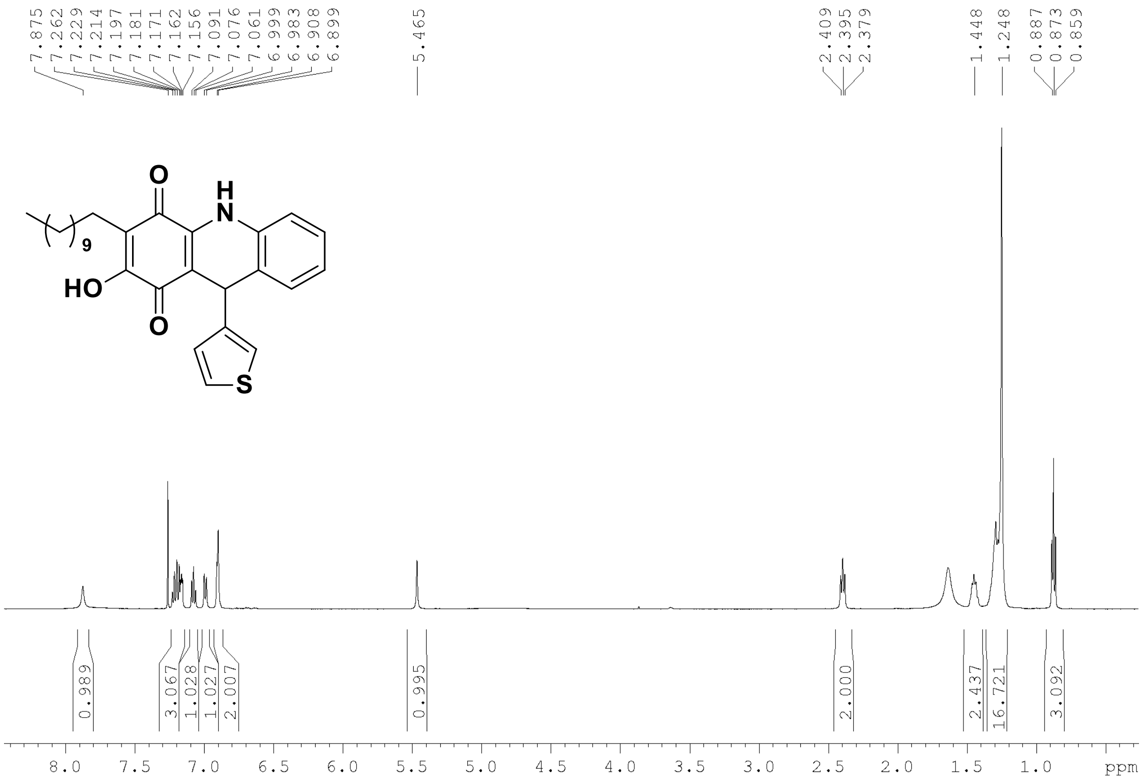

Figure S33. ^{13}C-NMR ($CDCl_3$, 125 MHz) spectrum of compound **4h**

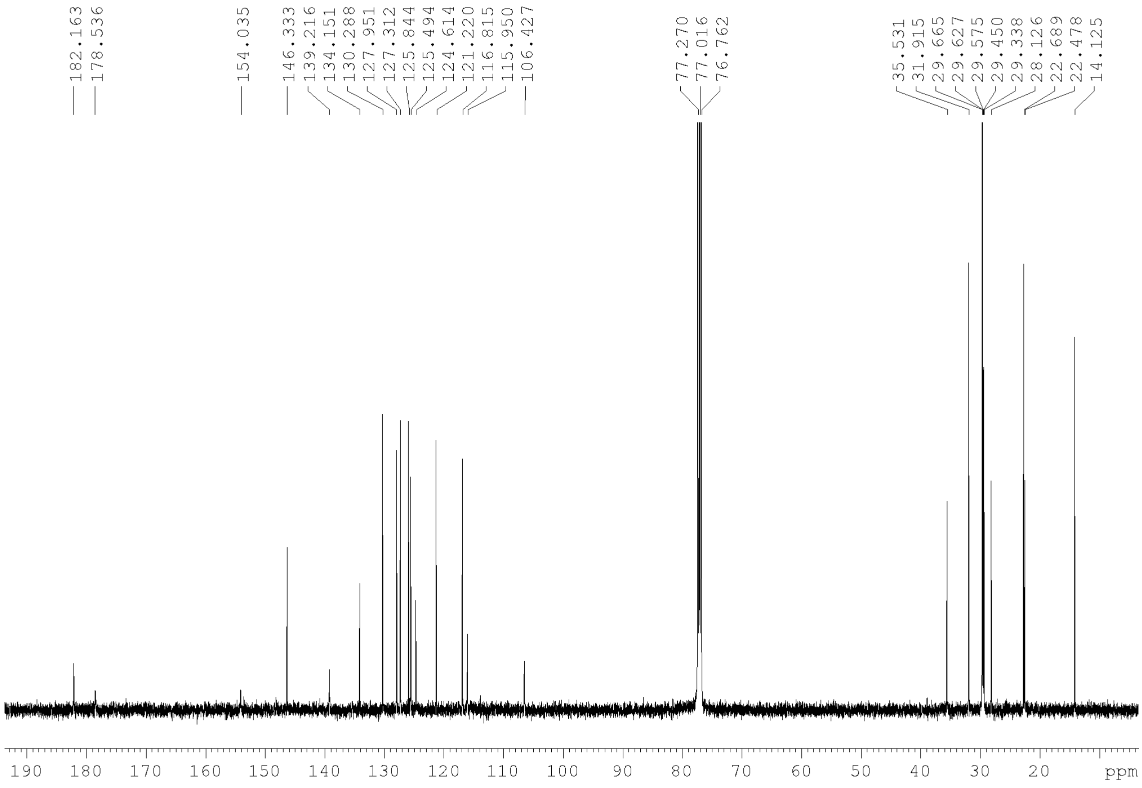

Figure S34. IR spectrum (film) of compound **4h**

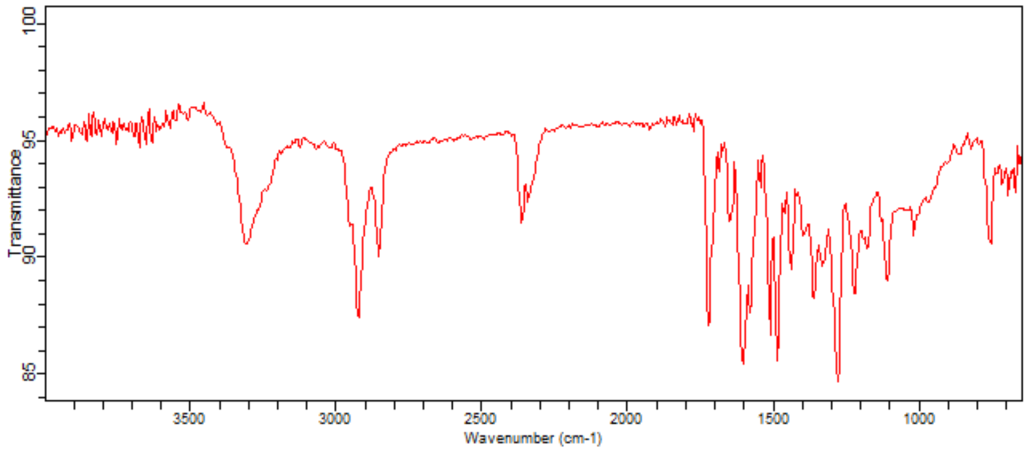

Figure S35. UV spectrum (EtOH) of compound **4h**

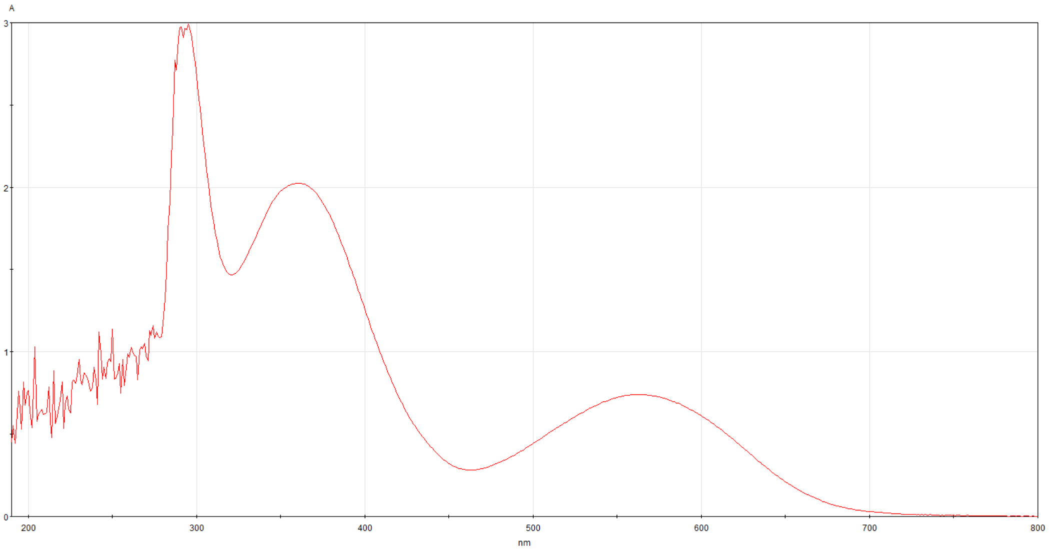

Figure S36. ^{1}H-NMR ($CDCl_3$, 500 MHz) spectrum of compound **4i**

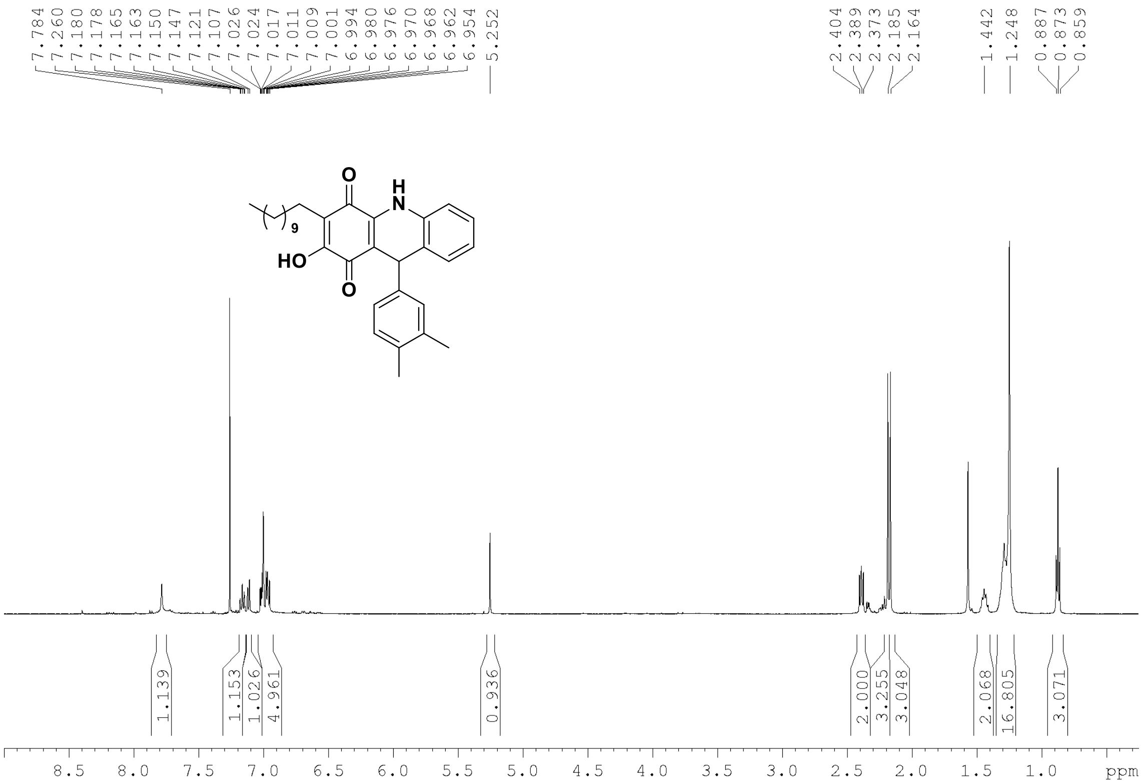

Figure S37. ^{13}C-NMR ($CDCl_3$, 125 MHz) spectrum of compound **4i**

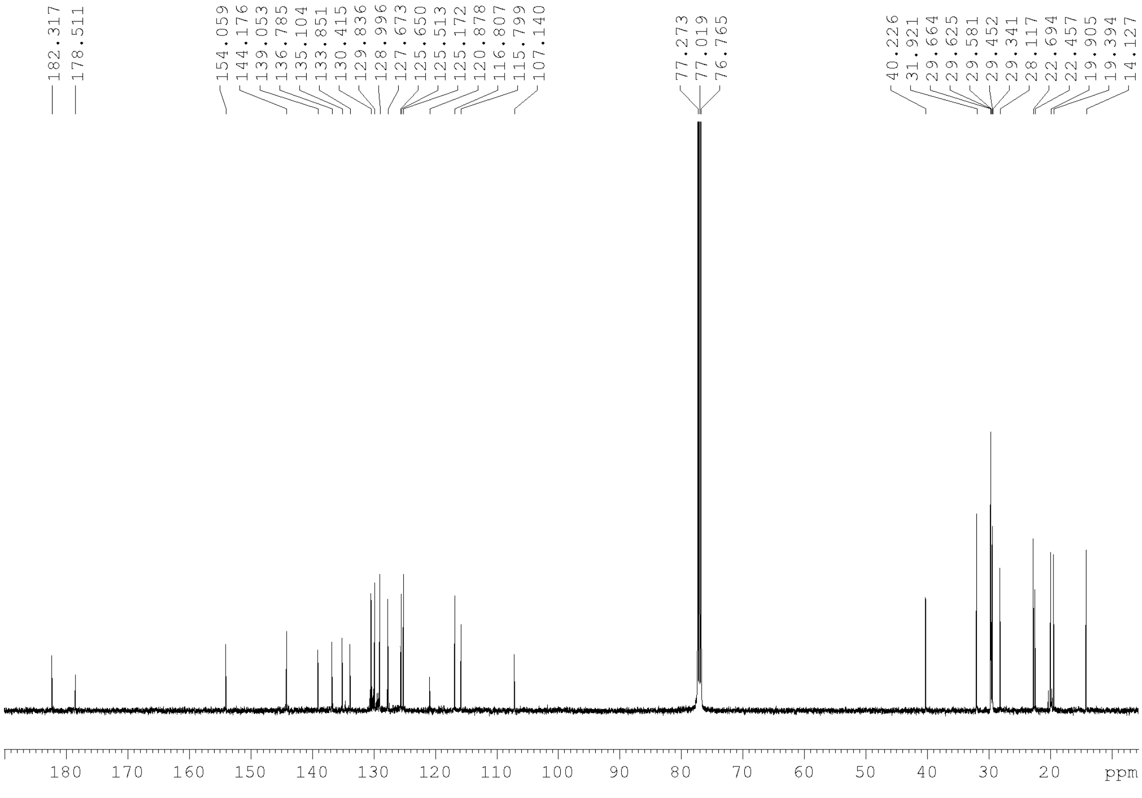

Figure S38. IR spectrum (film) of compound **4i**

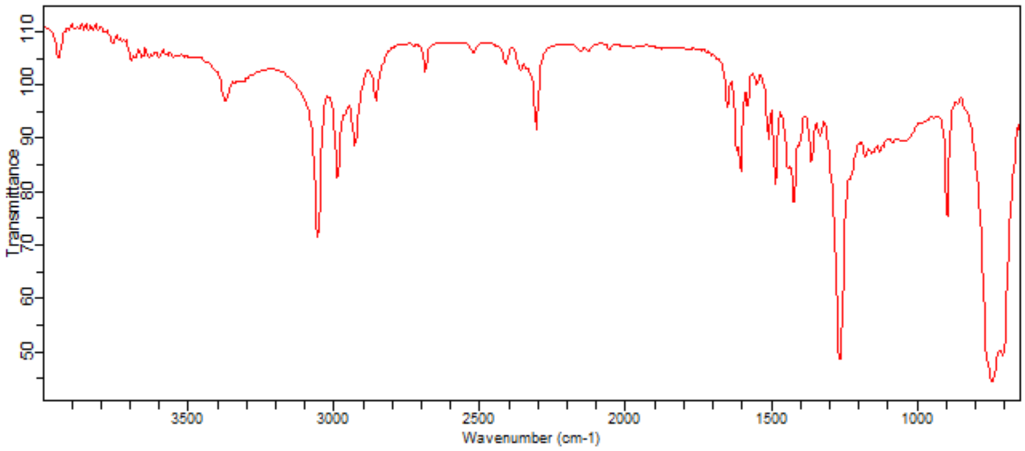

Figure S39. UV spectrum (EtOH) of compound **4i**

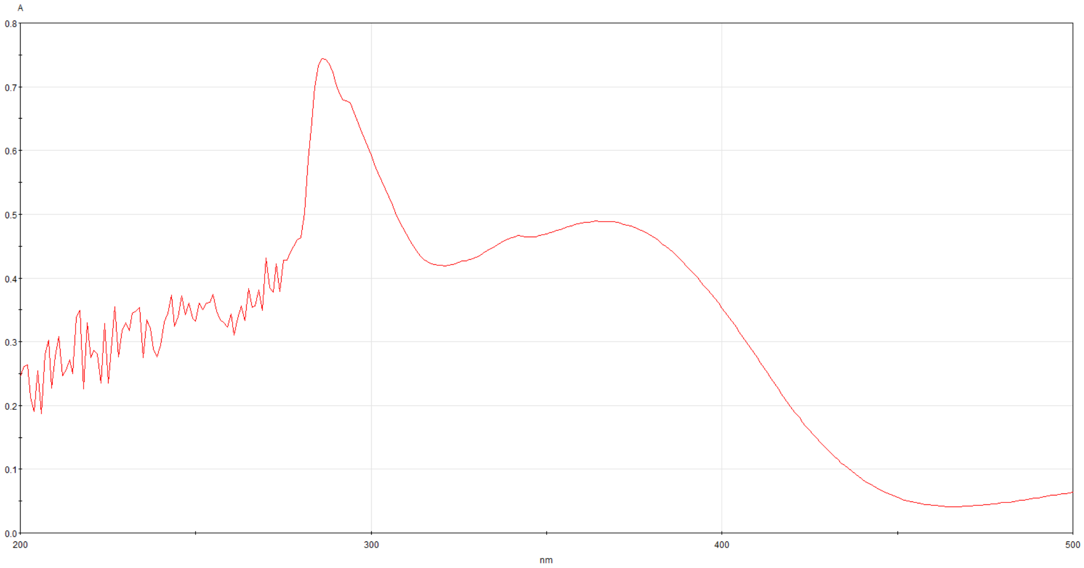

Figure S40. ^{1}H-NMR ($CDCl_3$, 500 MHz) spectrum of compound **4j**

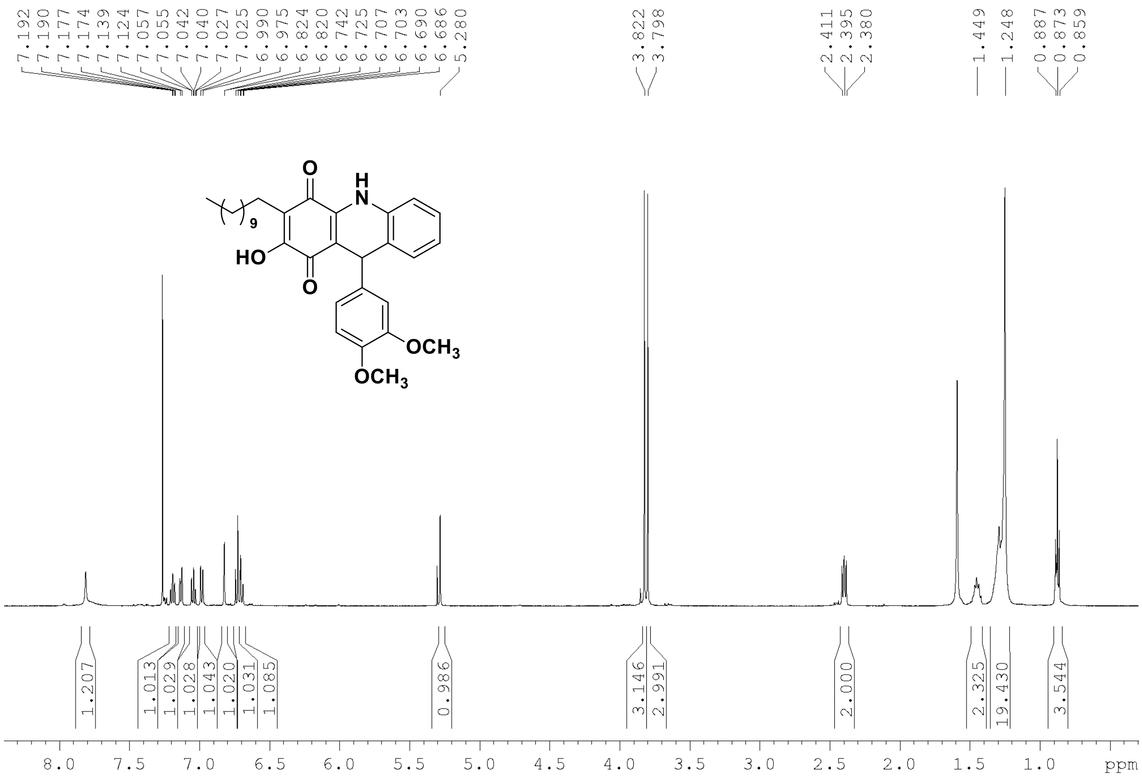

Figure S41. ^{13}C-NMR ($CDCl_3$, 150 MHz) spectrum of compound **4j**

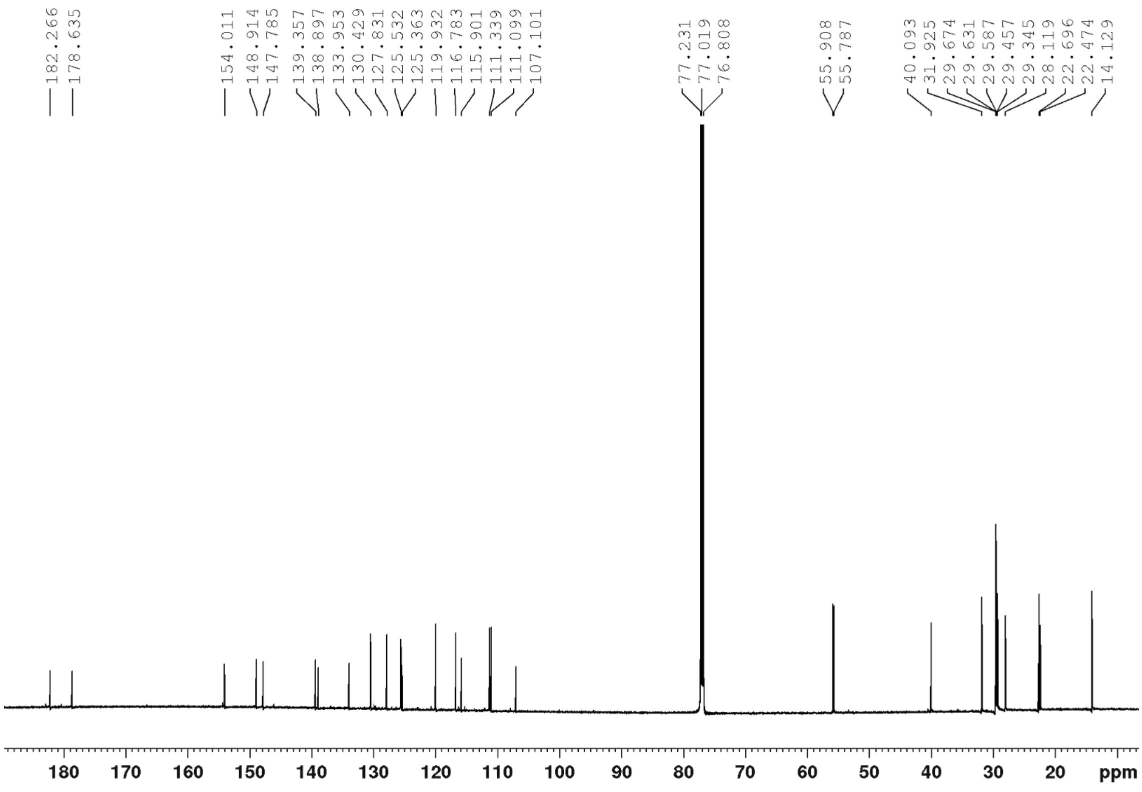

Figure S42. IR spectrum (film) of compound **4j**

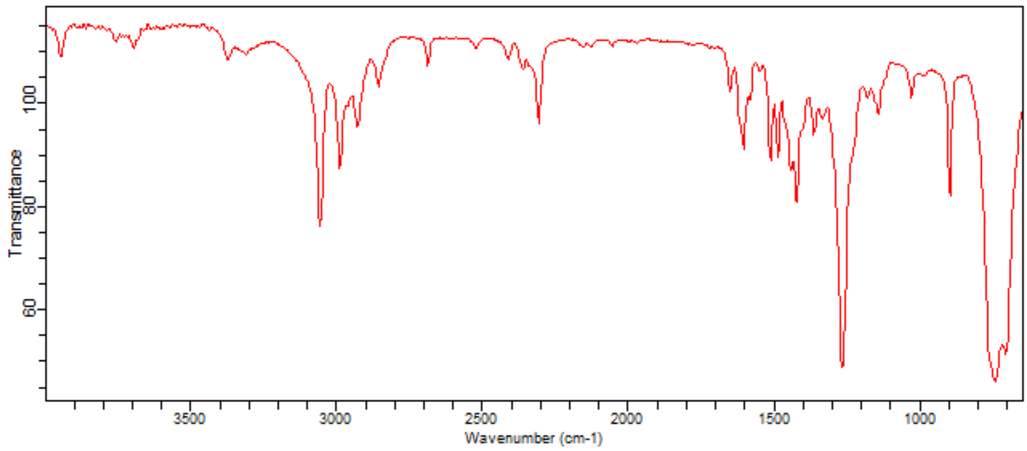

Figure S43. UV spectrum (EtOH) of compound **4j**

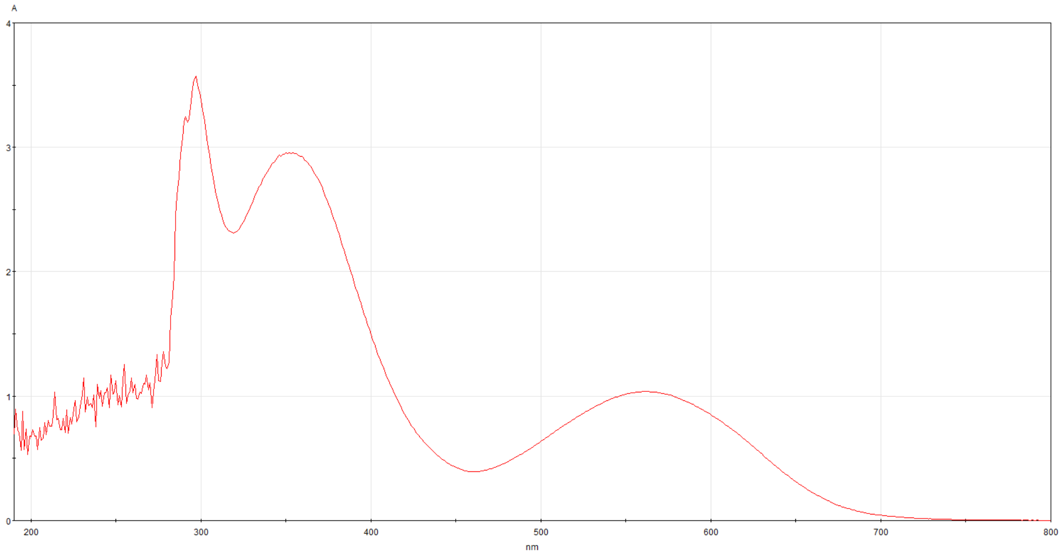

Figure S44. ^{1}H-NMR ($CDCl_3$, 500 MHz) spectrum of compound **4k**

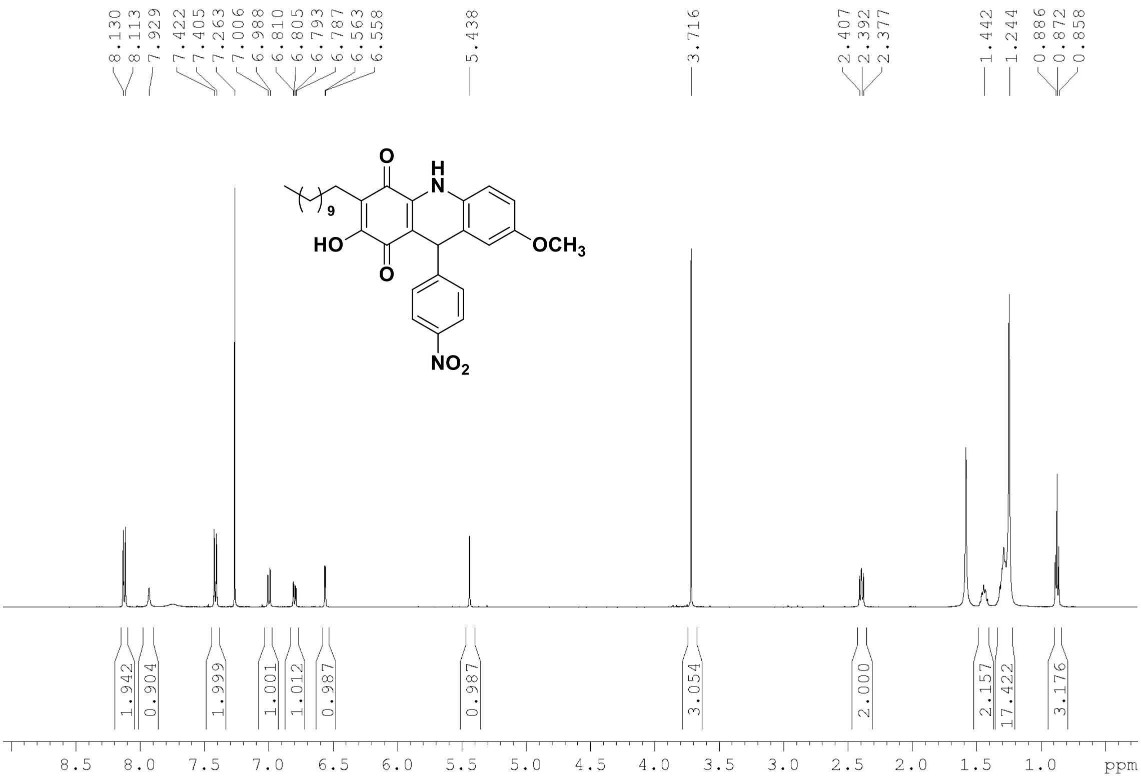

Figure S46. ^{13}C-NMR ($CDCl_3$, 125 MHz) spectrum of compound **4k**

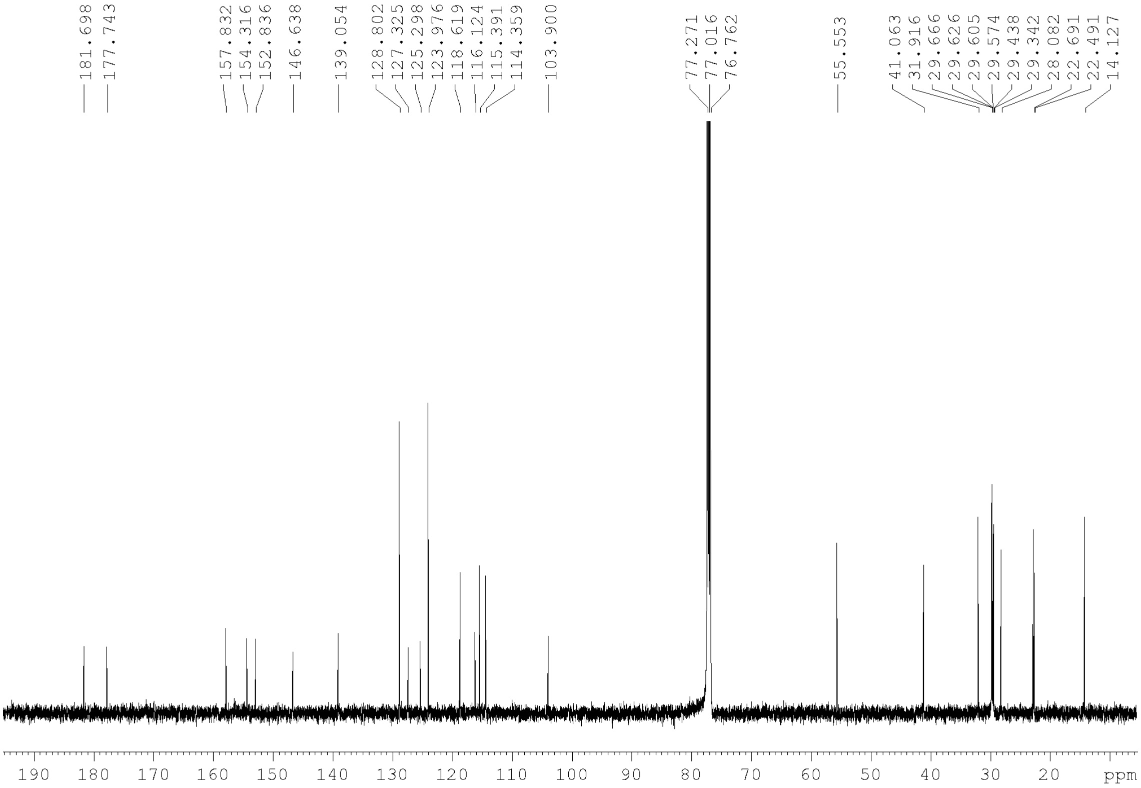

Figure S47. IR spectrum (film) of compound **4k**

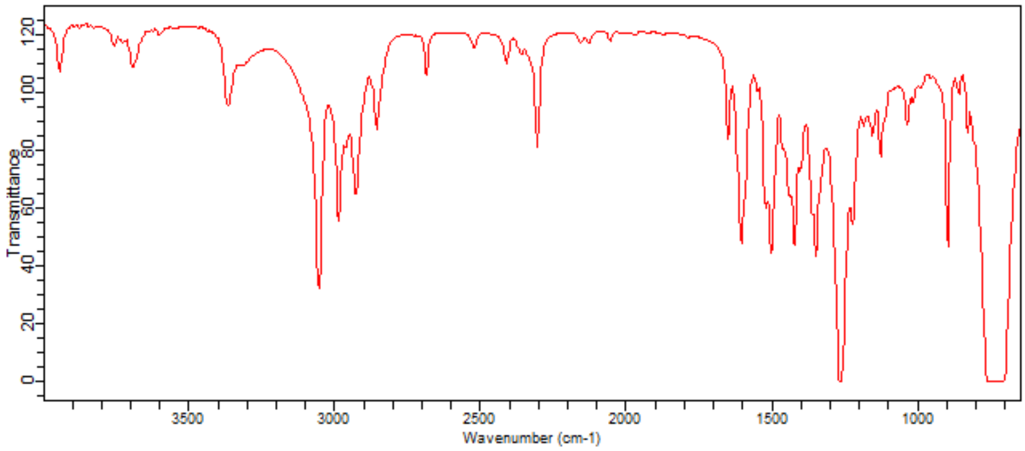

Figure S48. UV spectrum (EtOH) of compound **4k**

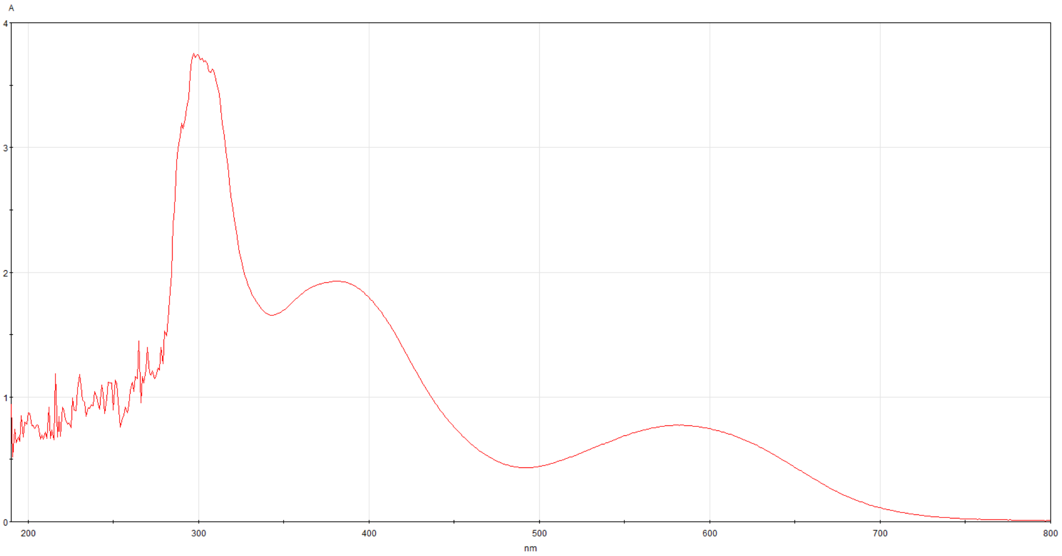

Figure S49. ^{1}H-NMR ($CDCl_3$, 500 MHz) spectrum of compound **5b**

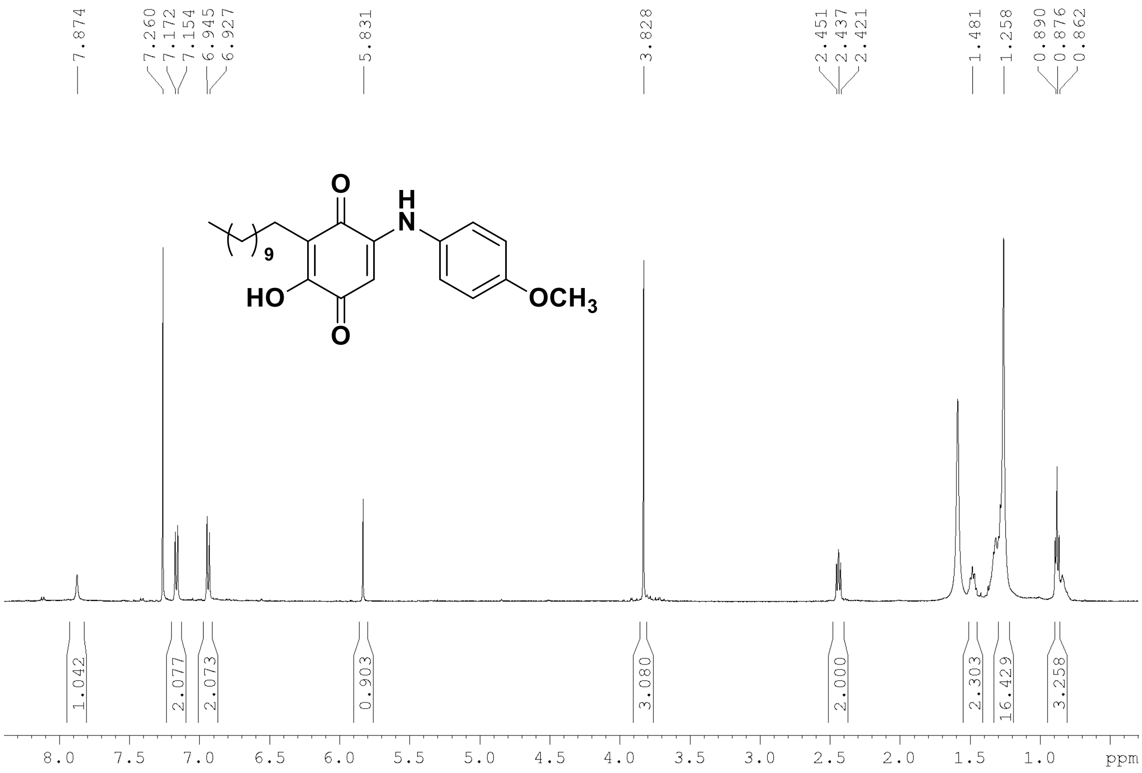

Figure S50. ^{13}C-NMR (125 MHz, $CDCl_3$) spectrum of compound **5b**

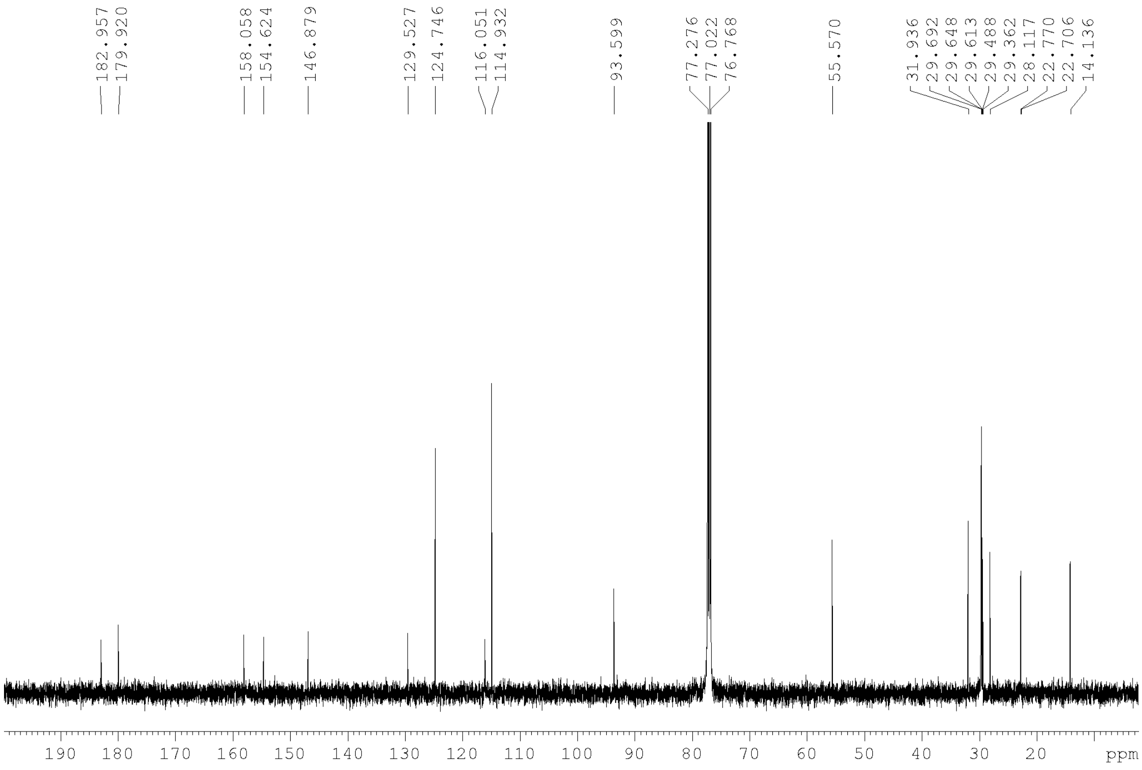

Figure S51.^{1}H-NMR ($CDCl_3$, 500 MHz) spectrum of compound **4l**

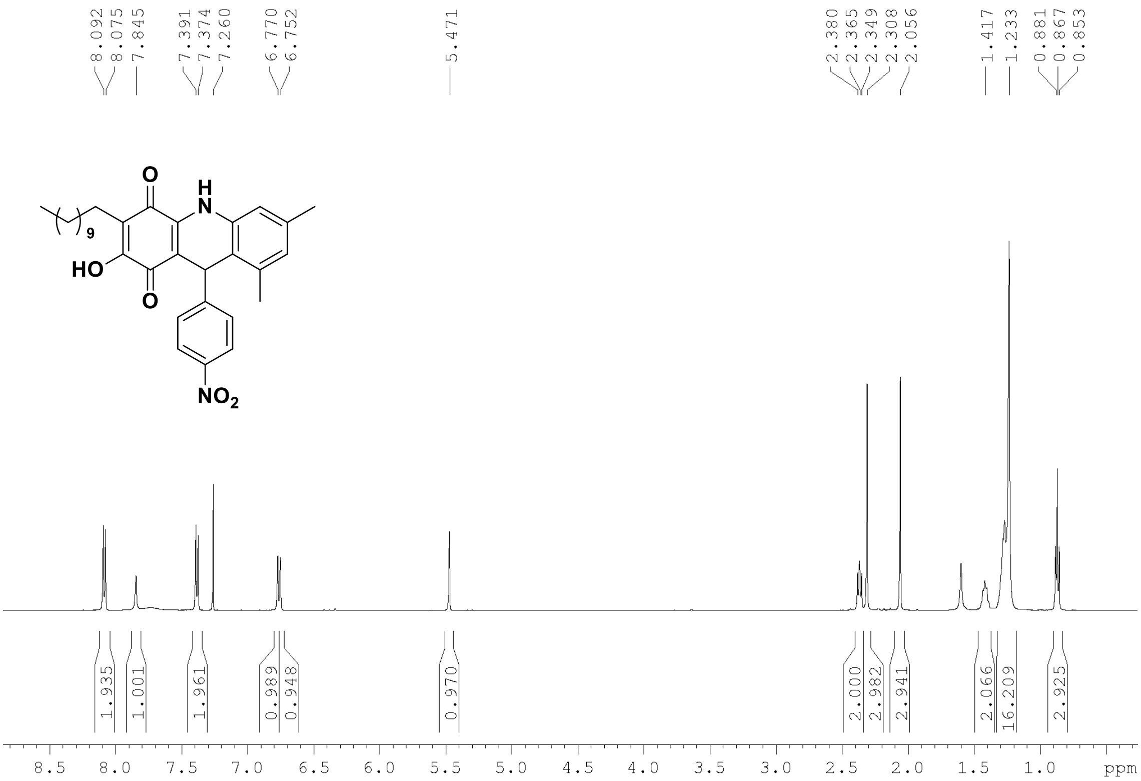

Figure S52.^{13}C-NMR ($CDCl_3$, 125 MHz) spectrum of compound **4l**

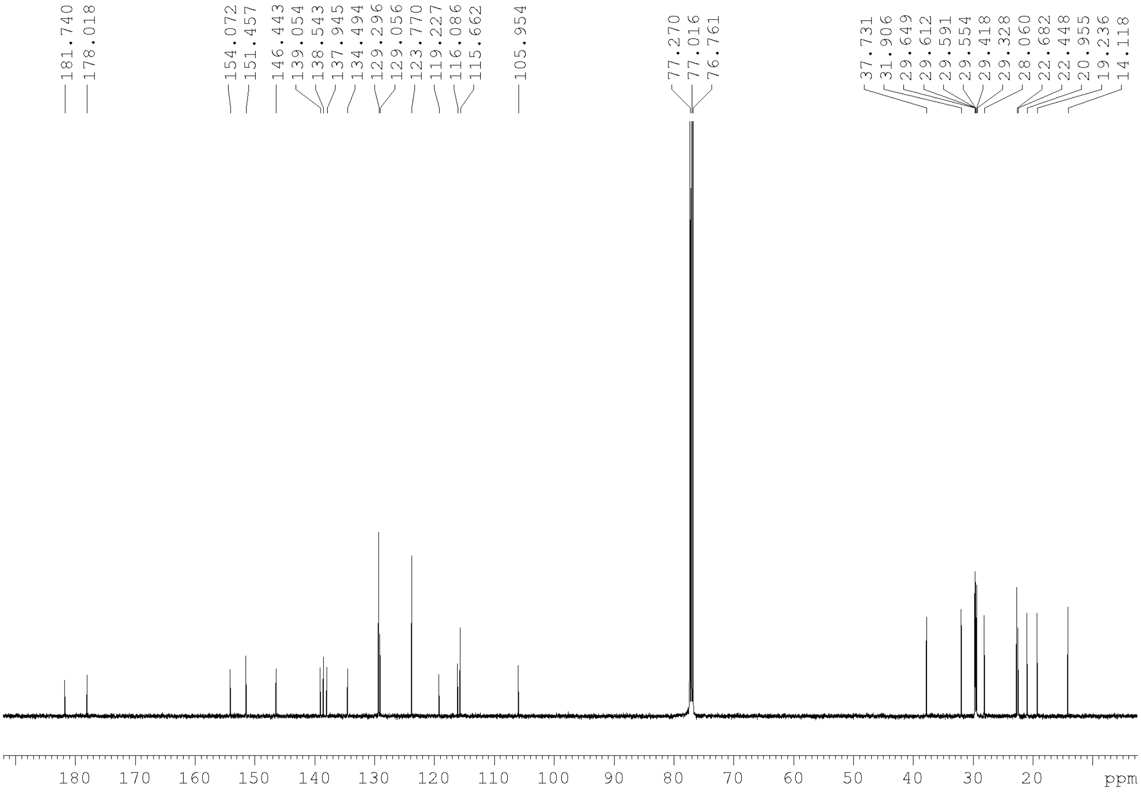

Figure S53. IR spectrum (film) of compound **4l**

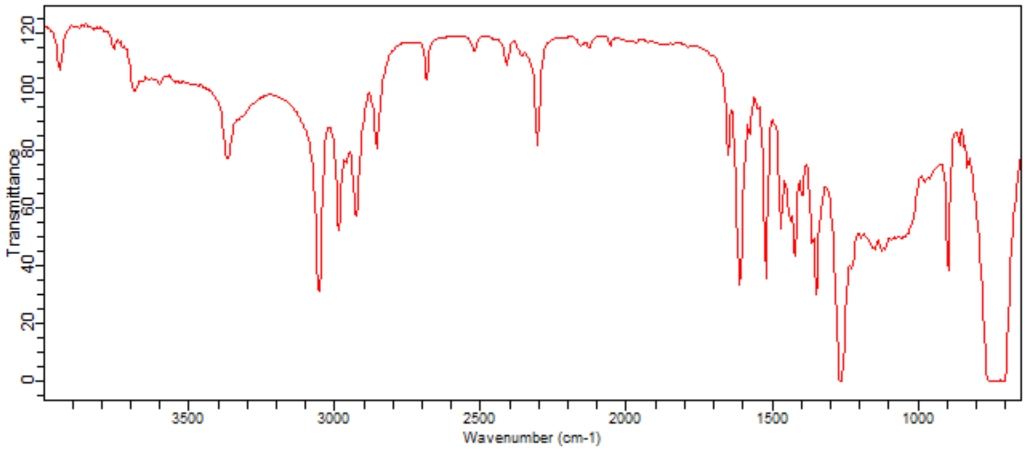

Figure S54. UV spectrum (EtOH) of compound **4l**

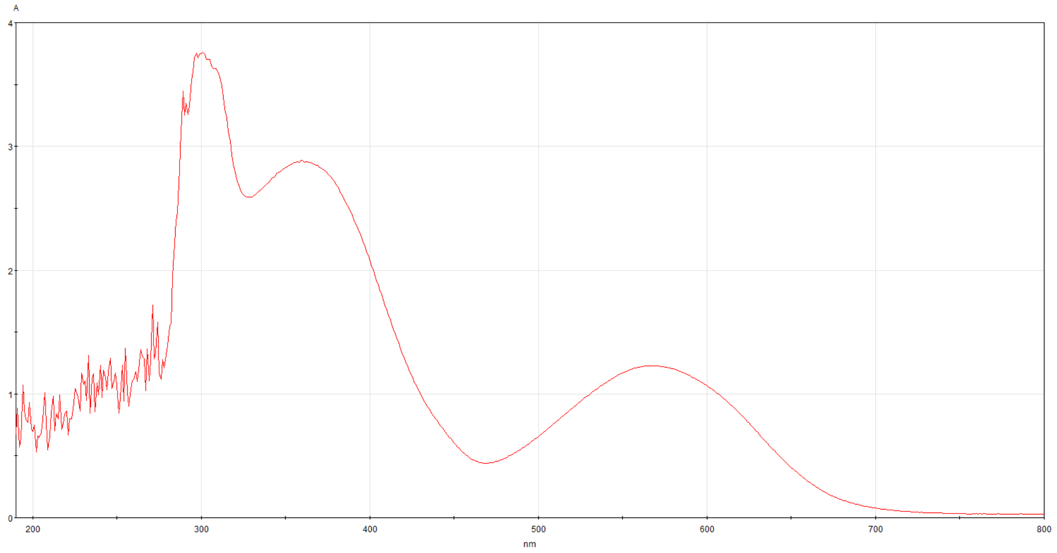

Figure S55. ^{1}H-NMR ($CDCl_3$, 500 MHz) spectrum of compound **4m**

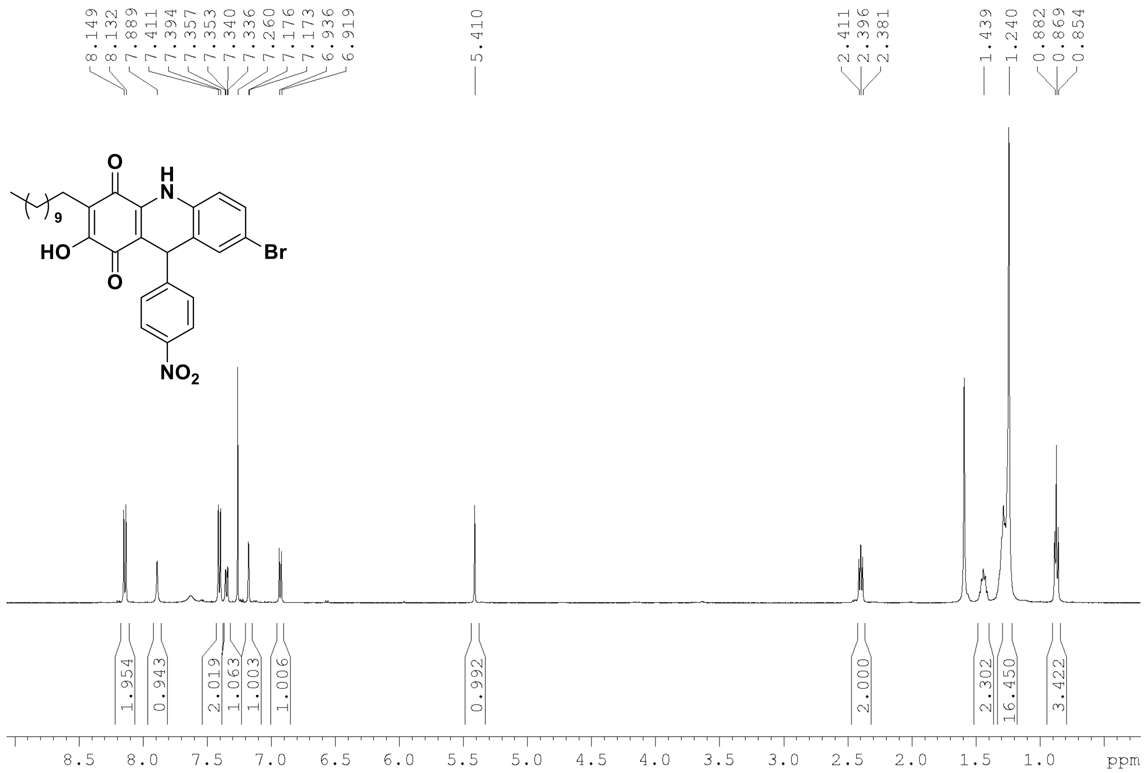

Figure S56. ^{13}C-NMR ($CDCl_3$, 125 MHz) spectrum of compound **4m**

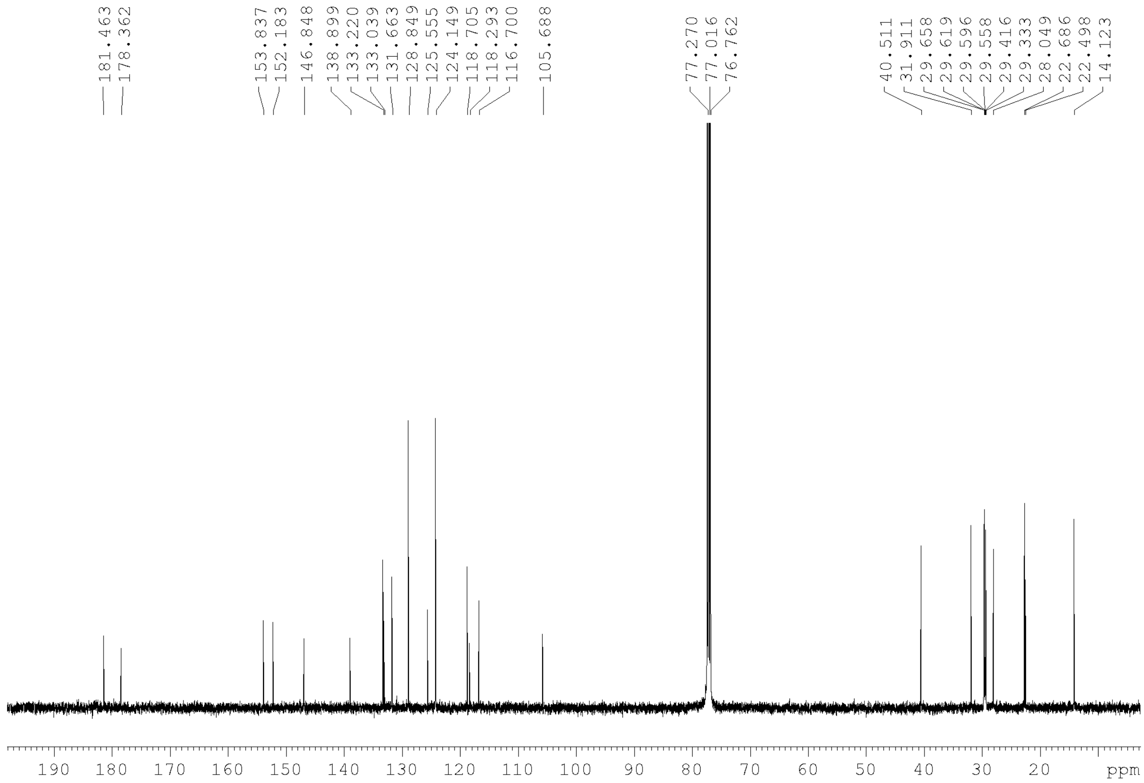

Figure S57. IR spectrum (film) of compound **4m**

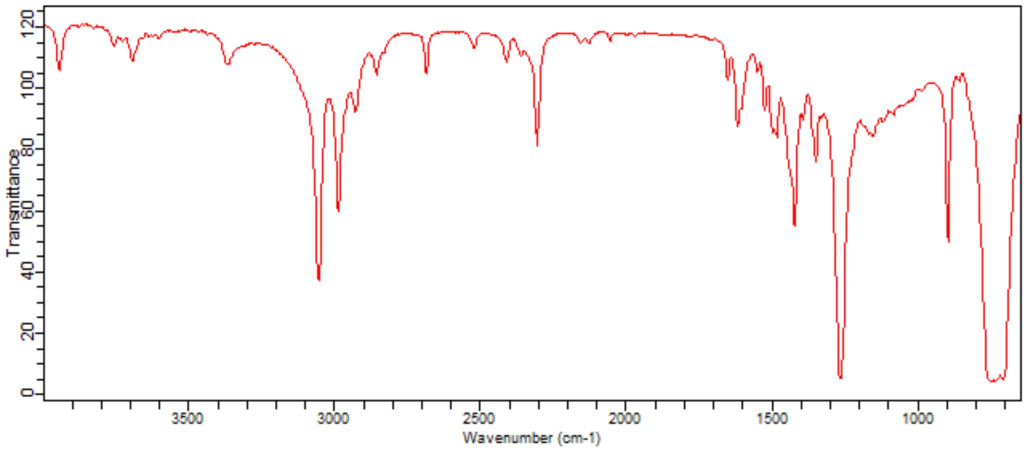

Figure S58. UV spectrum (EtOH) of compound **4m**

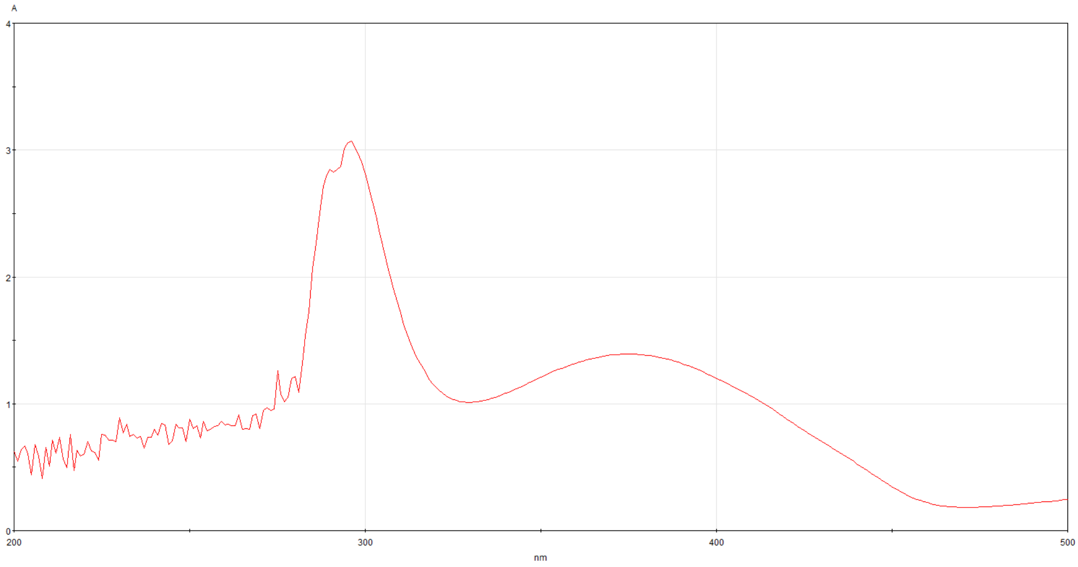

Figure S59. ^{1}H-NMR ($CDCl_3$, 500 MHz) spectrum of compound **5c**

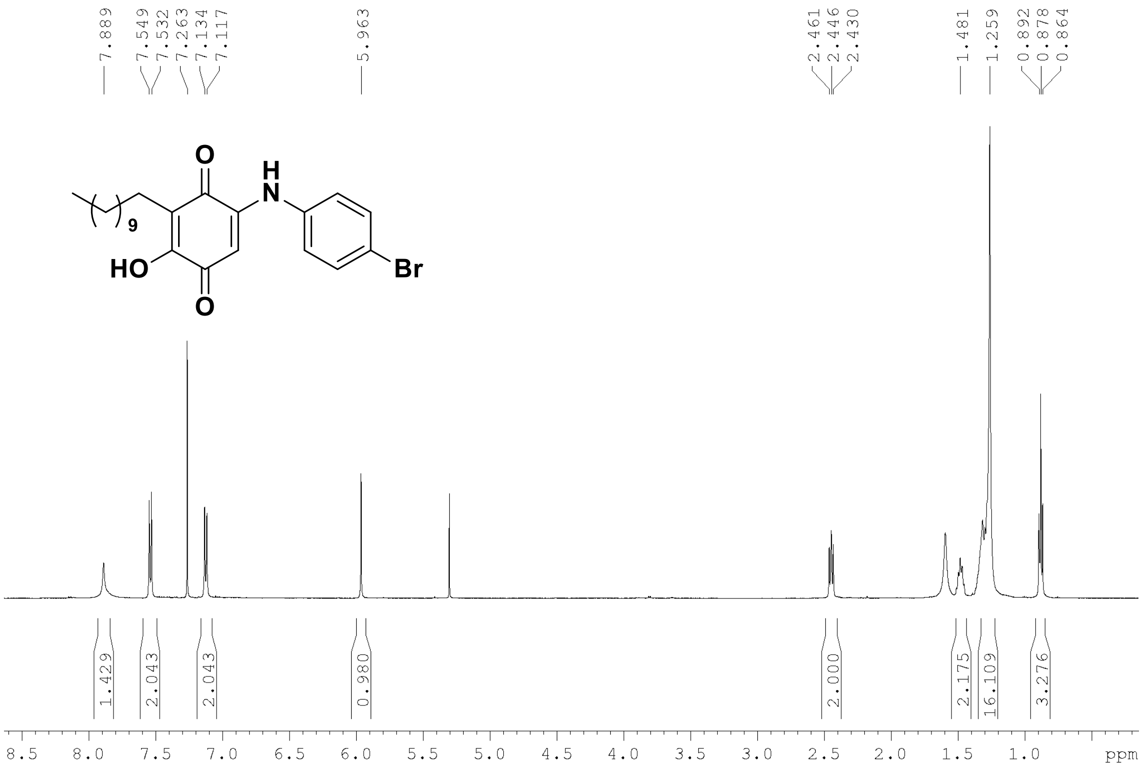

Figure S60. ^{13}C-NMR ($CDCl_3$, 125 MHz) spectrum of compound **5c**

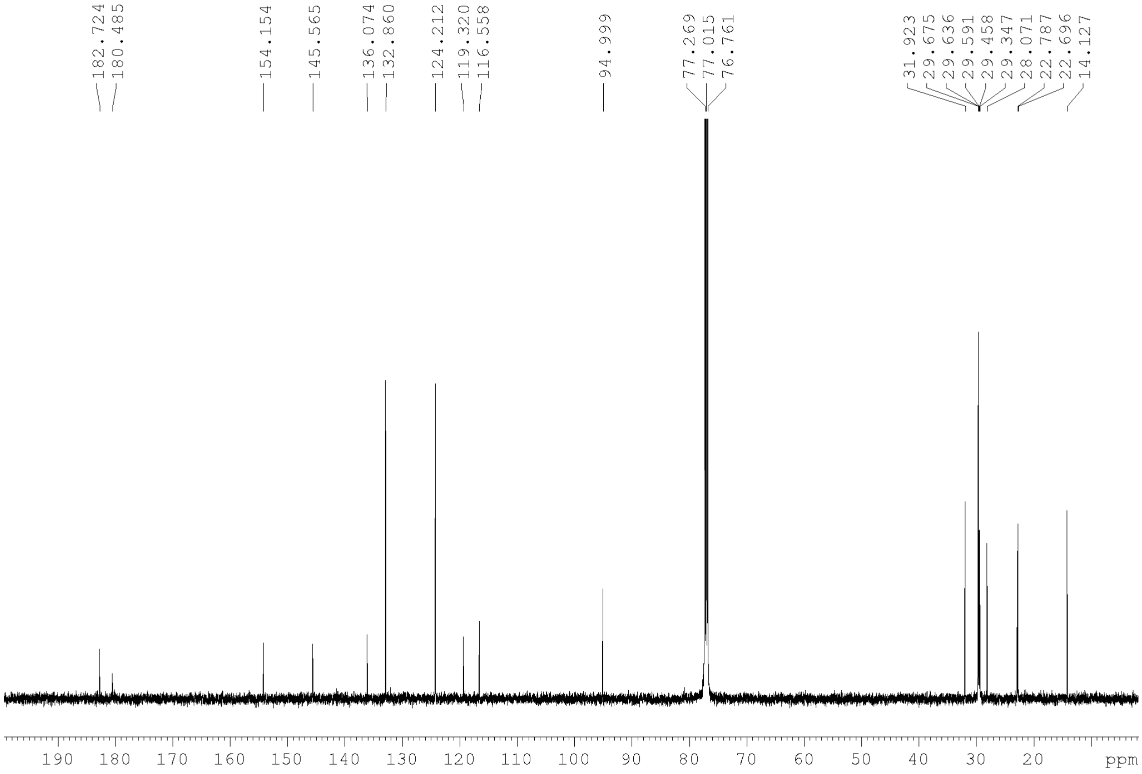

Figure S61. ^{1}H-NMR ($CDCl_3$, 500 MHz) spectrum of compound **5d**

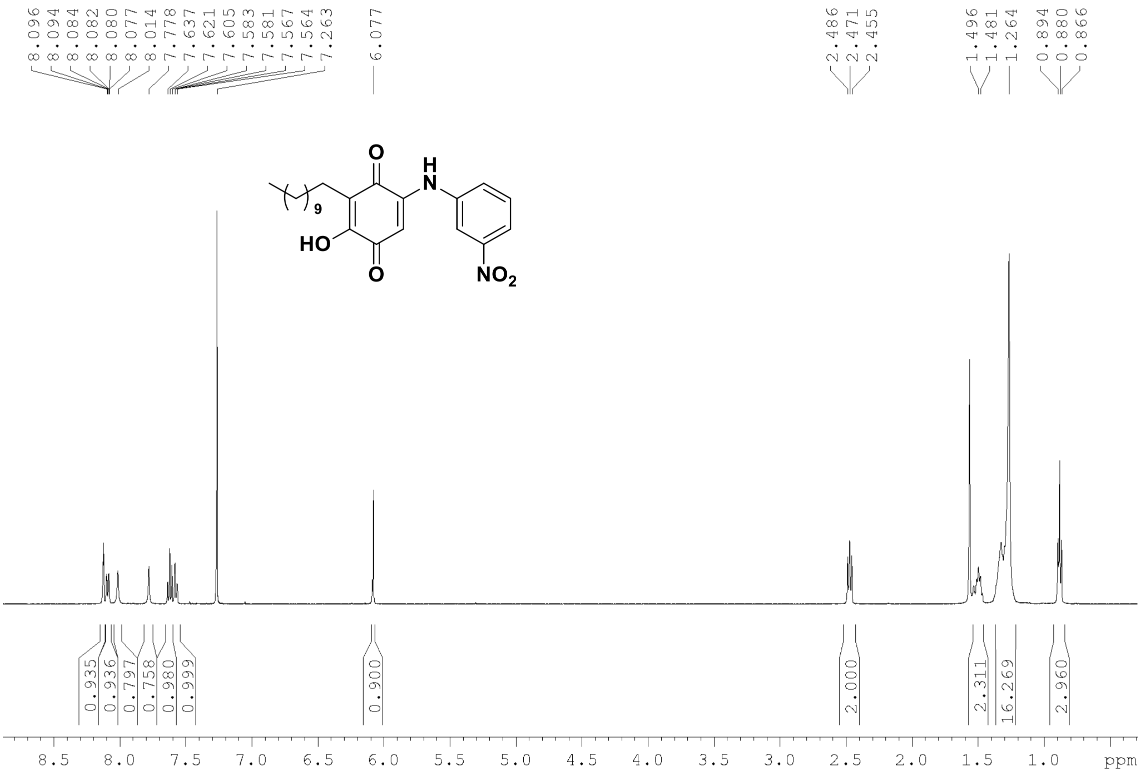

Figure S62. ^{13}C-NMR ($CDCl_3$, 150 MHz) spectrum of compound **5d**

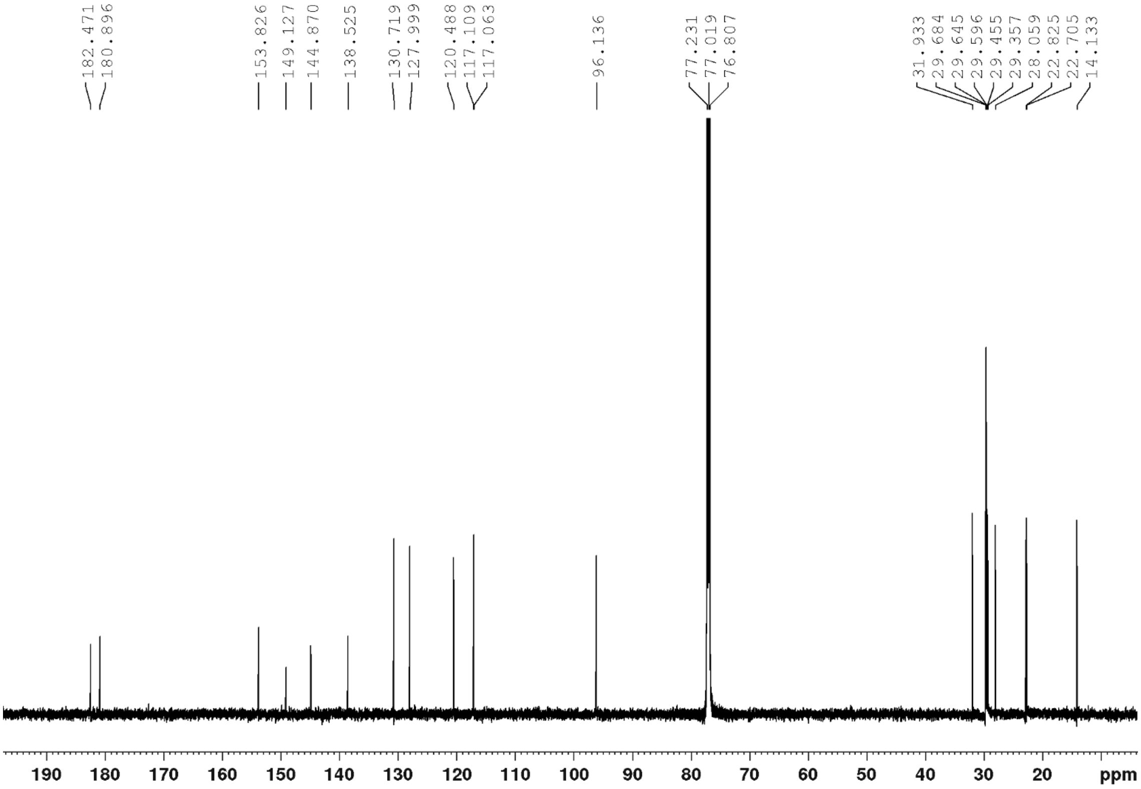

Figure S63. ^{1}H-NMR ($CDCl_3$, 500 MHz) spectrum of compound **6a**

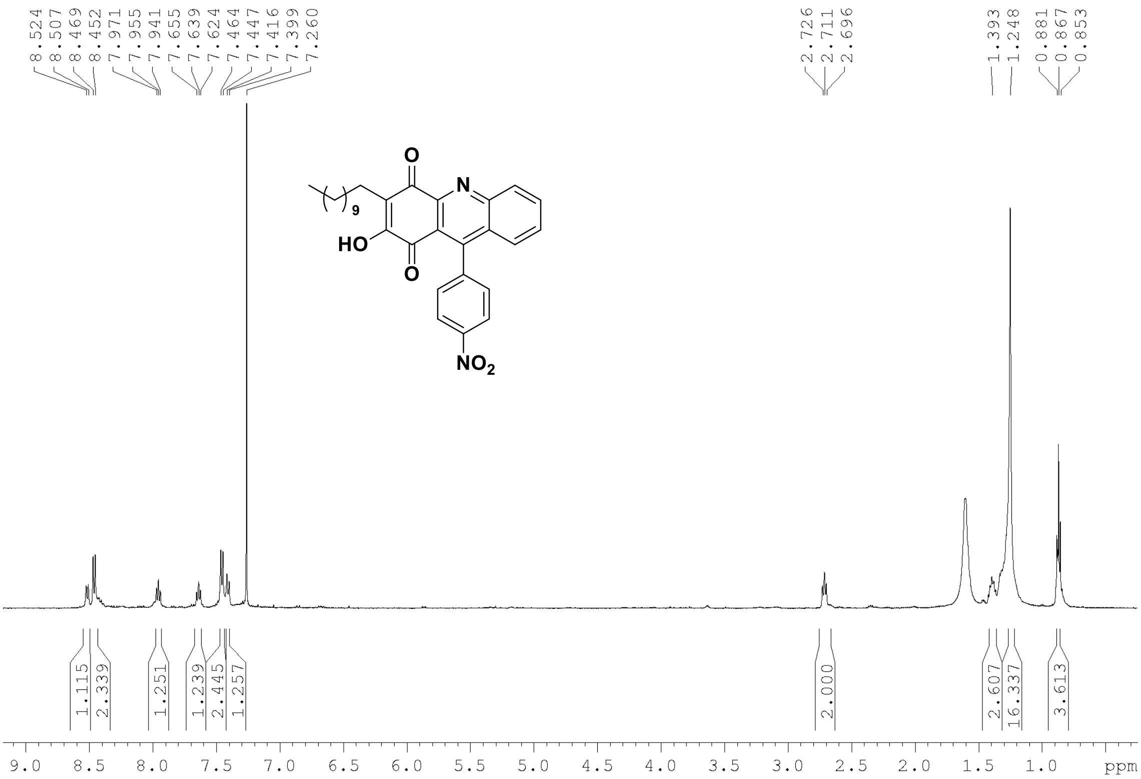

Figure S64. ^{13}C-NMR ($CDCl_3$, 150 MHz) spectrum of compound **6a**

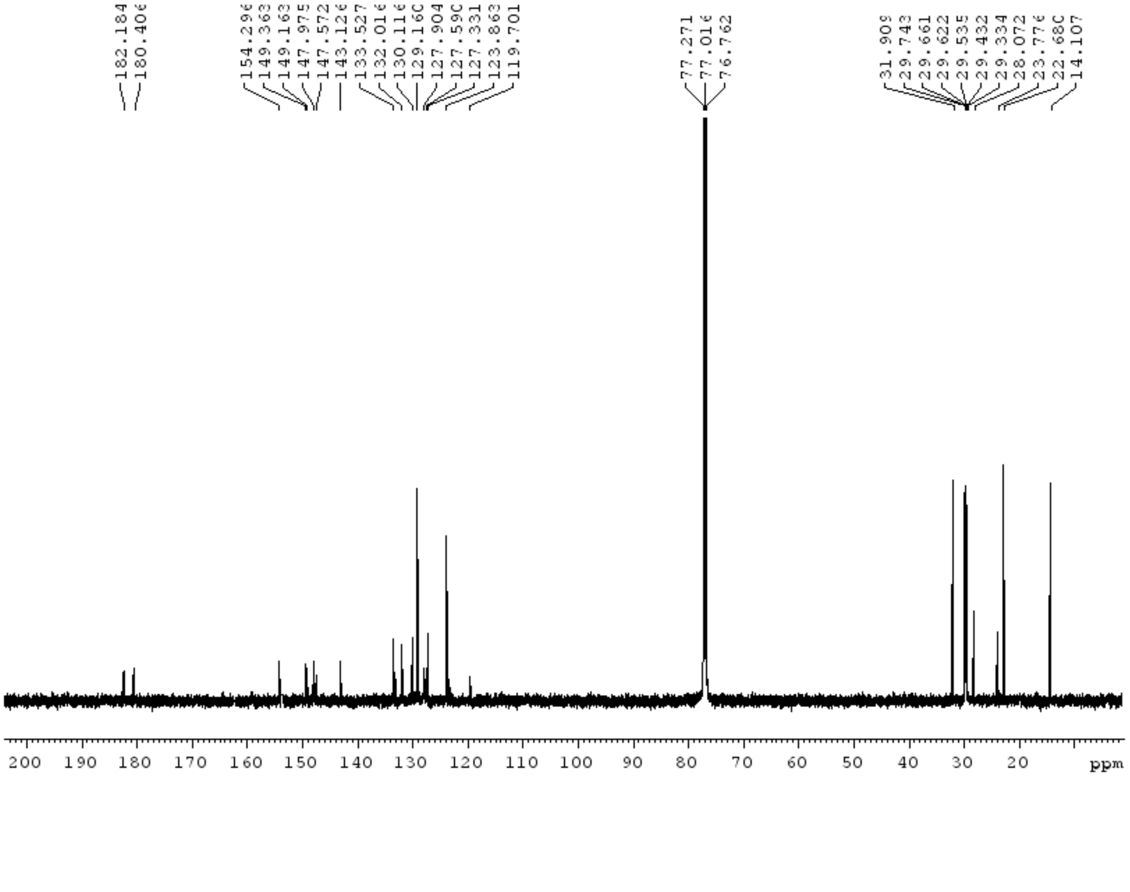

Figure S65. IR spectrum (film) of compound **6a**

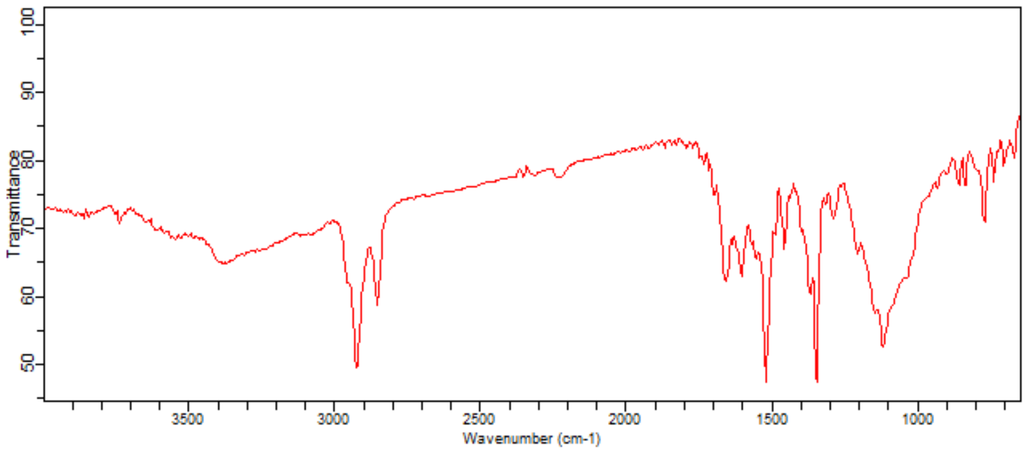

Figure S66. UV spectrum (EtOH) of compound **6a**

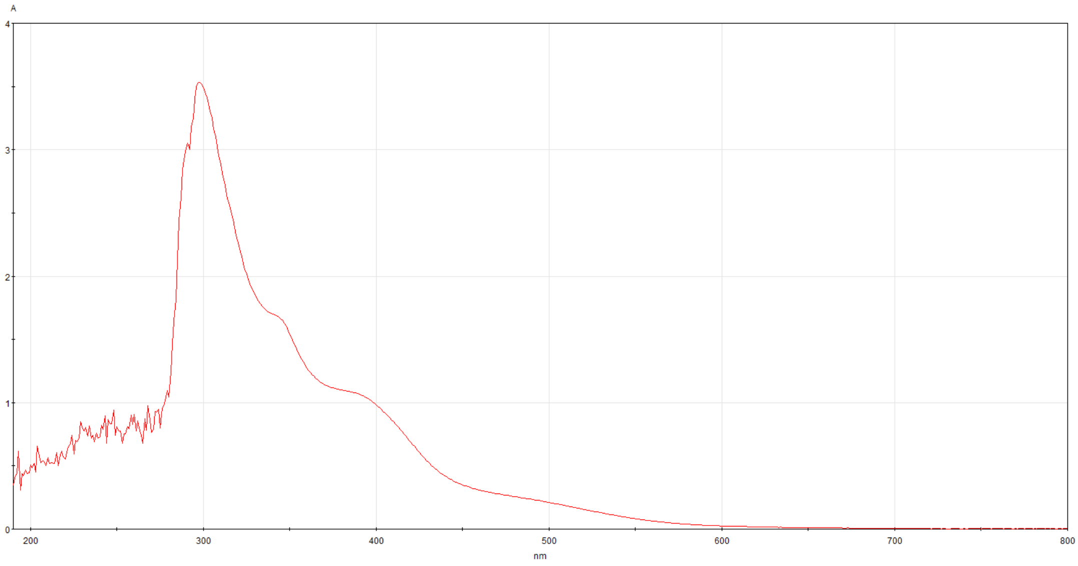

Figure S67. ^{1}H-NMR ($CDCl_3$, 500 MHz) spectrum of compound **6b**

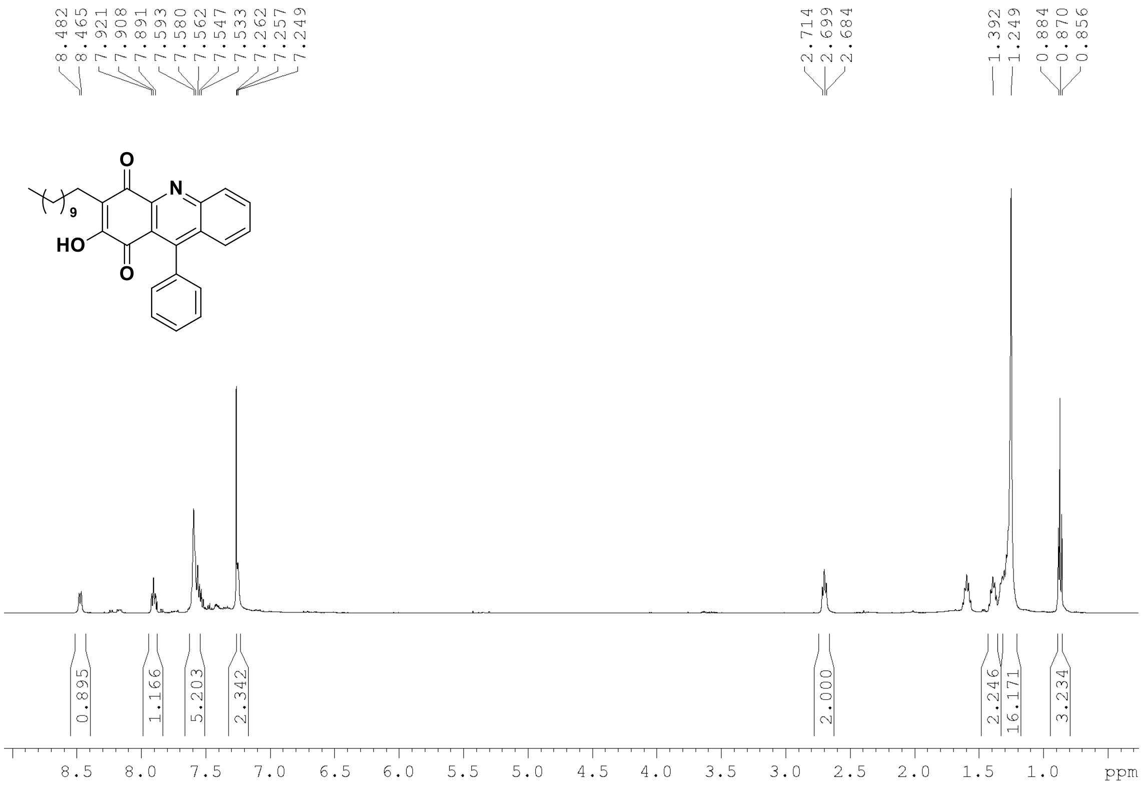

Figure S68. ^{13}C-NMR ($CDCl_3$, 150 MHz) spectrum of compound **6b**

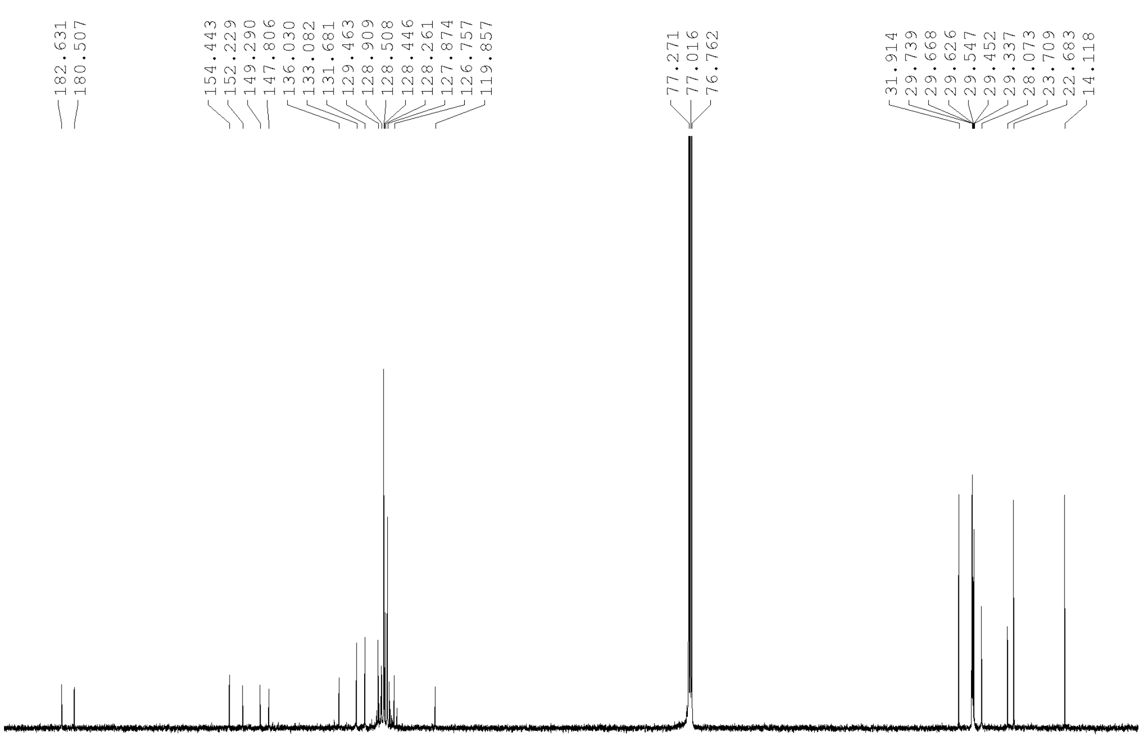

Figure S69. IR spectrum (film) of compound **6b**

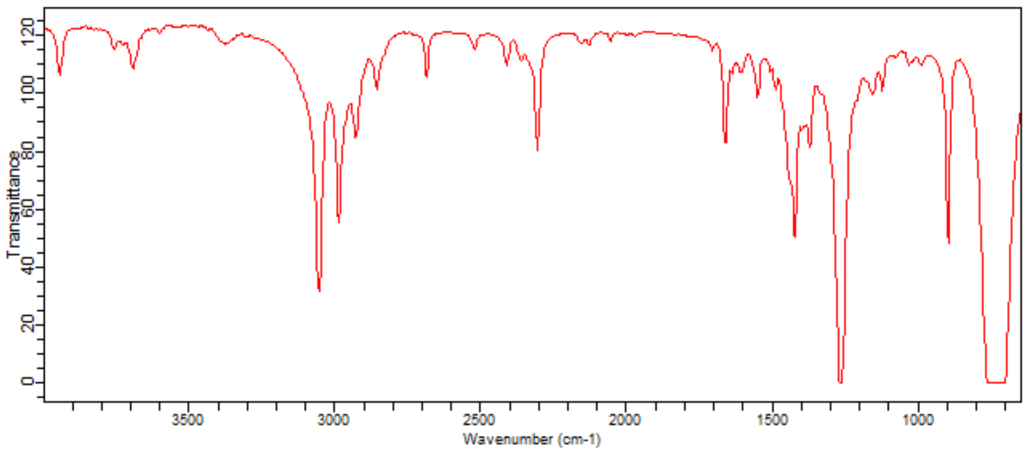

Figure S70. IR spectrum (EtOH) of compound **6b**

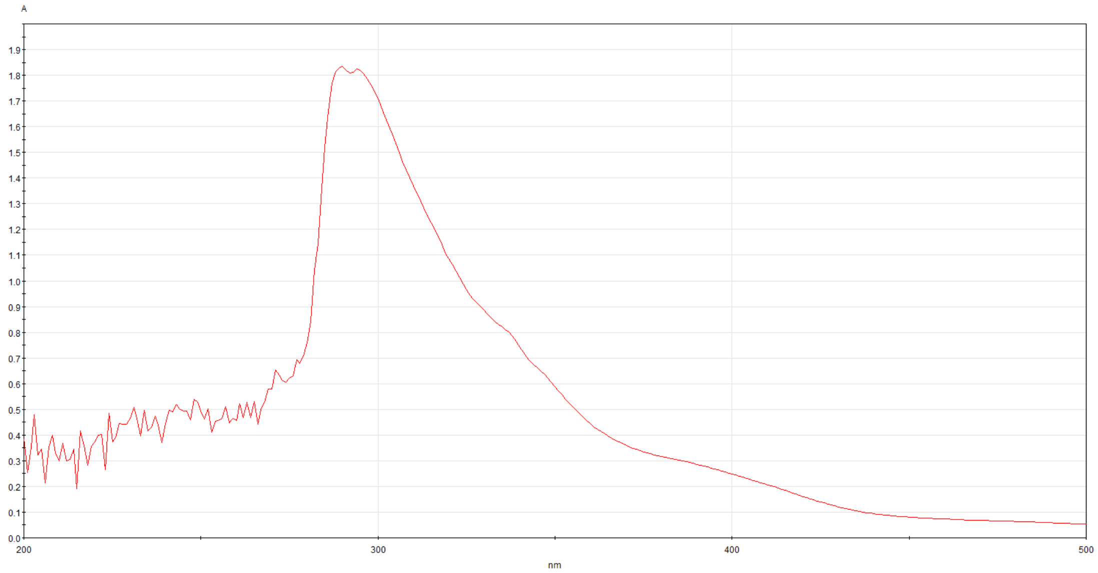

Figure S71. ^{1}H-NMR ($CDCl_3$, 500 MHz) spectrum of compound **6c**

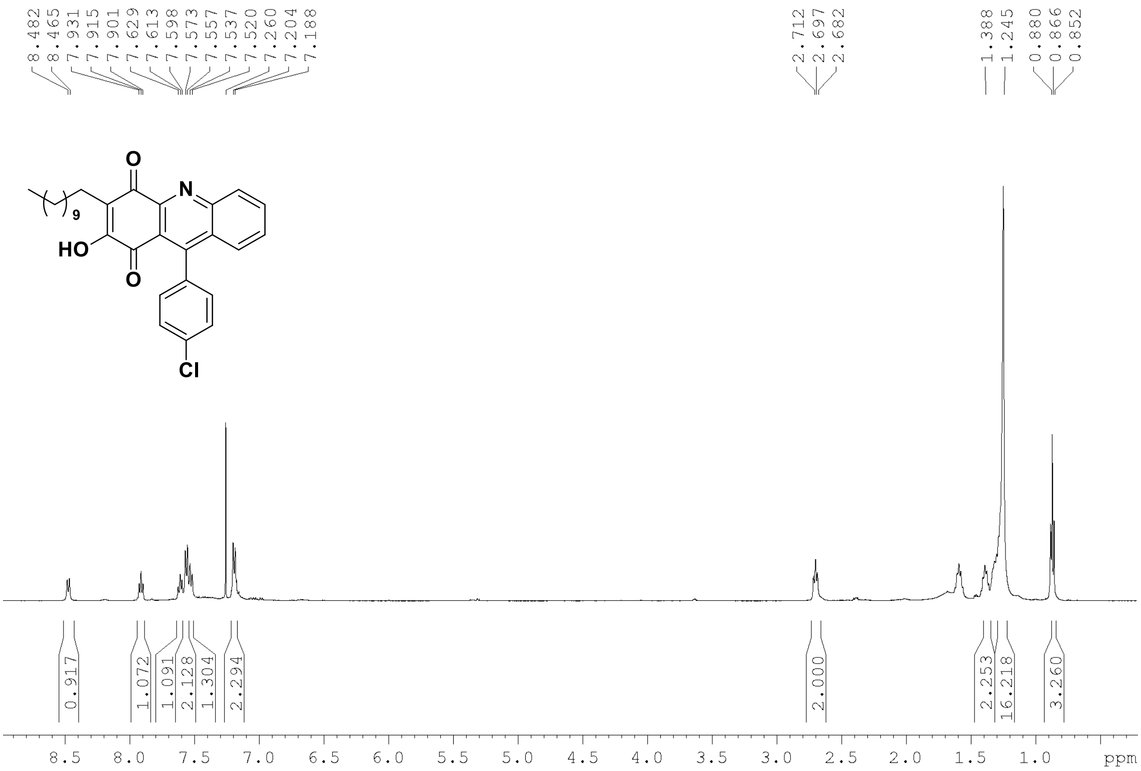

Figure S72. ^{13}C-NMR ($CDCl_3$, 150 MHz) spectrum of compound **6c**

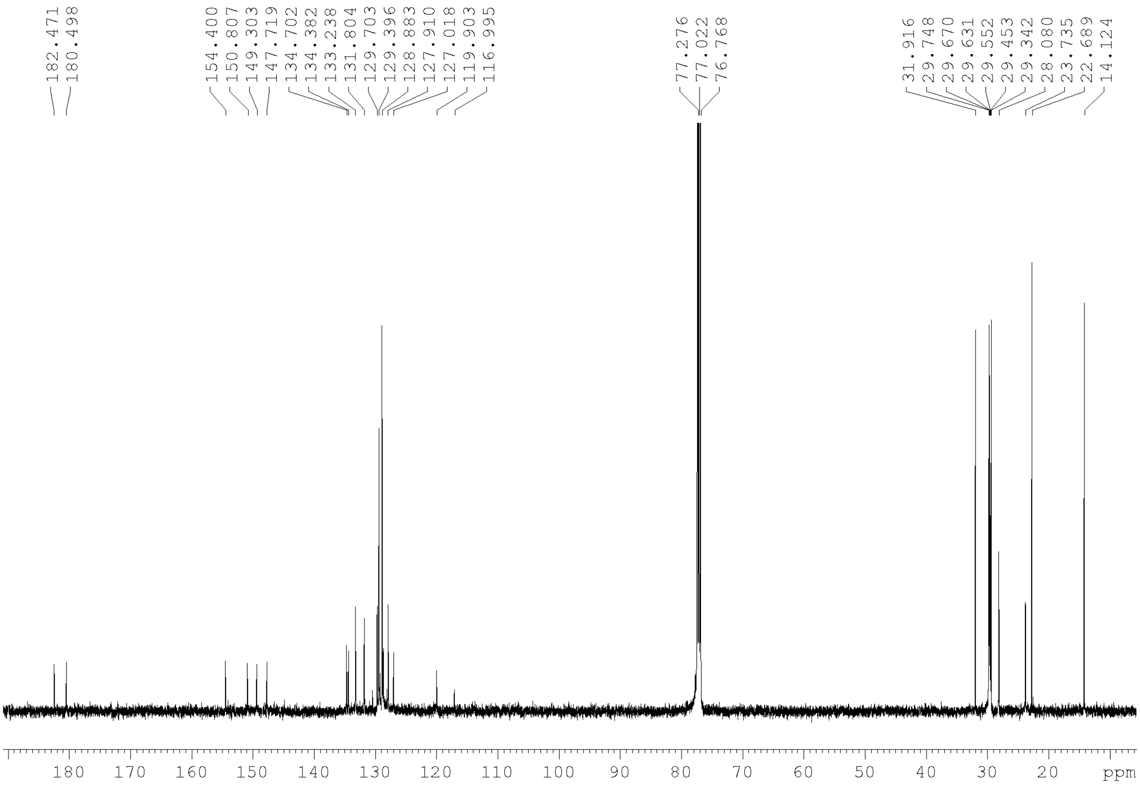

Figure S73. IR spectrum (film) of compound **6c**

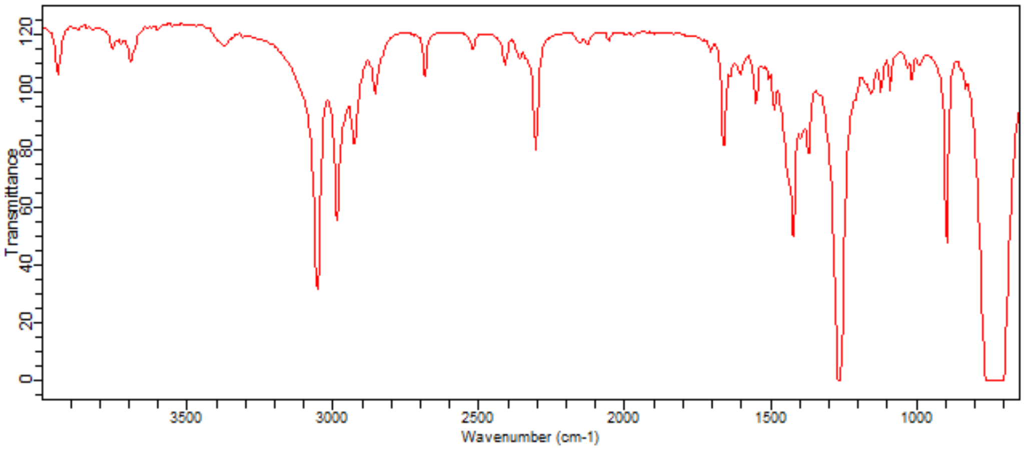

Figure S74. UV spectrum (EtOH) of compound **6c**

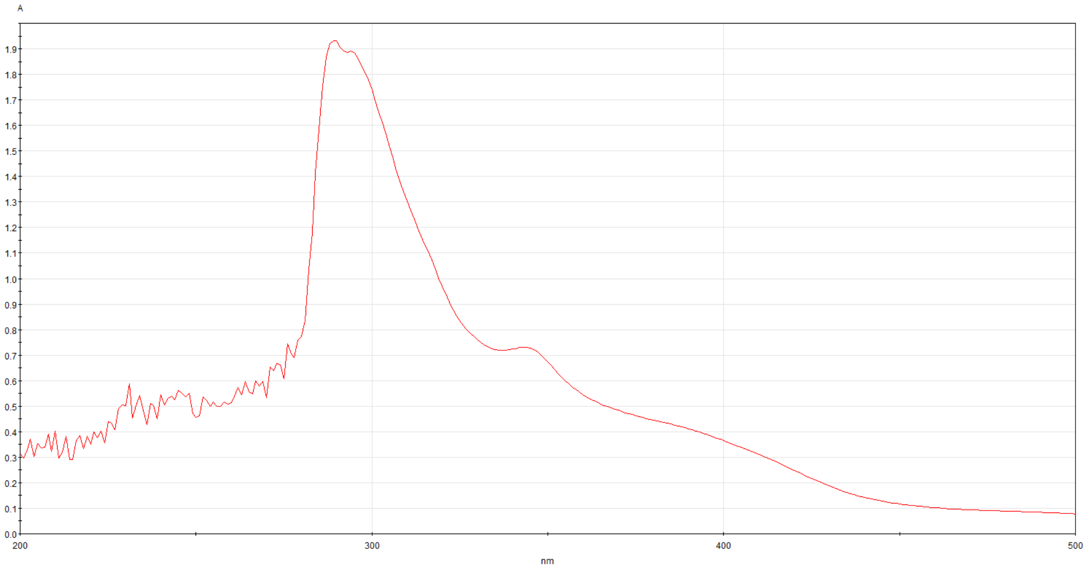

Figure S75. ^{1}H-NMR ($CDCl_3$, 500 MHz) spectrum of compound **6d**

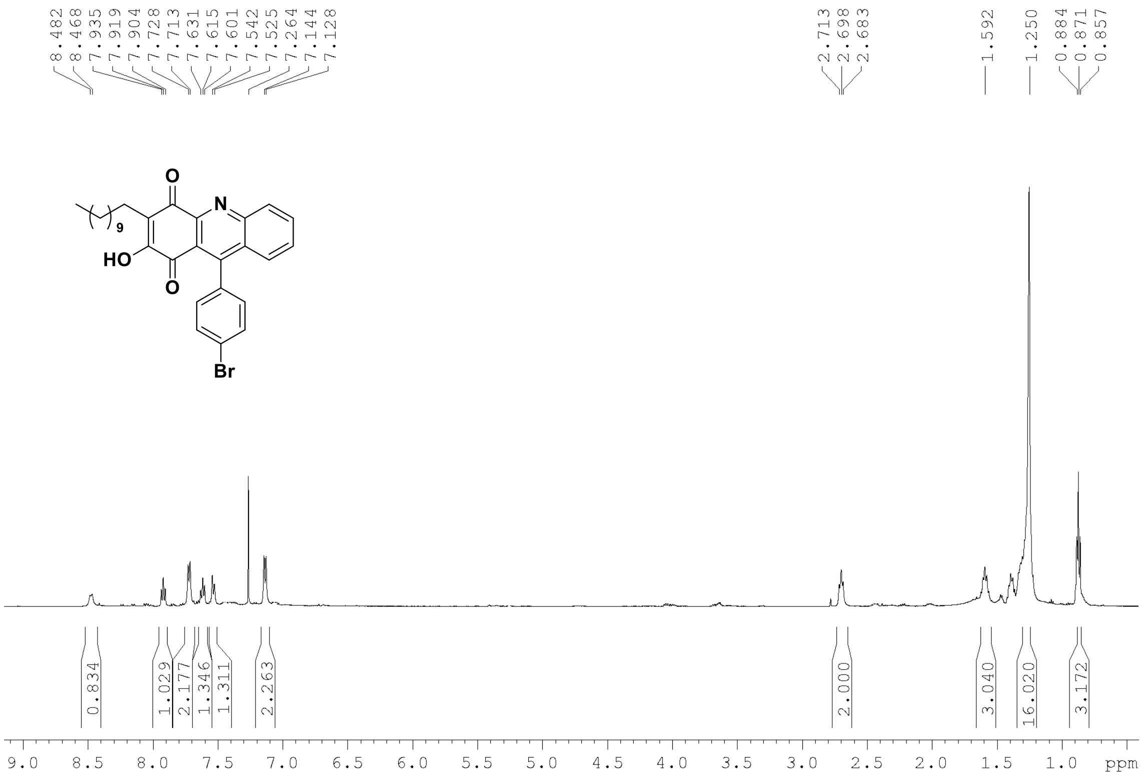

Figure S76. ^{13}C-NMR ($CDCl_3$, 150 MHz) spectrum of compound **6d**

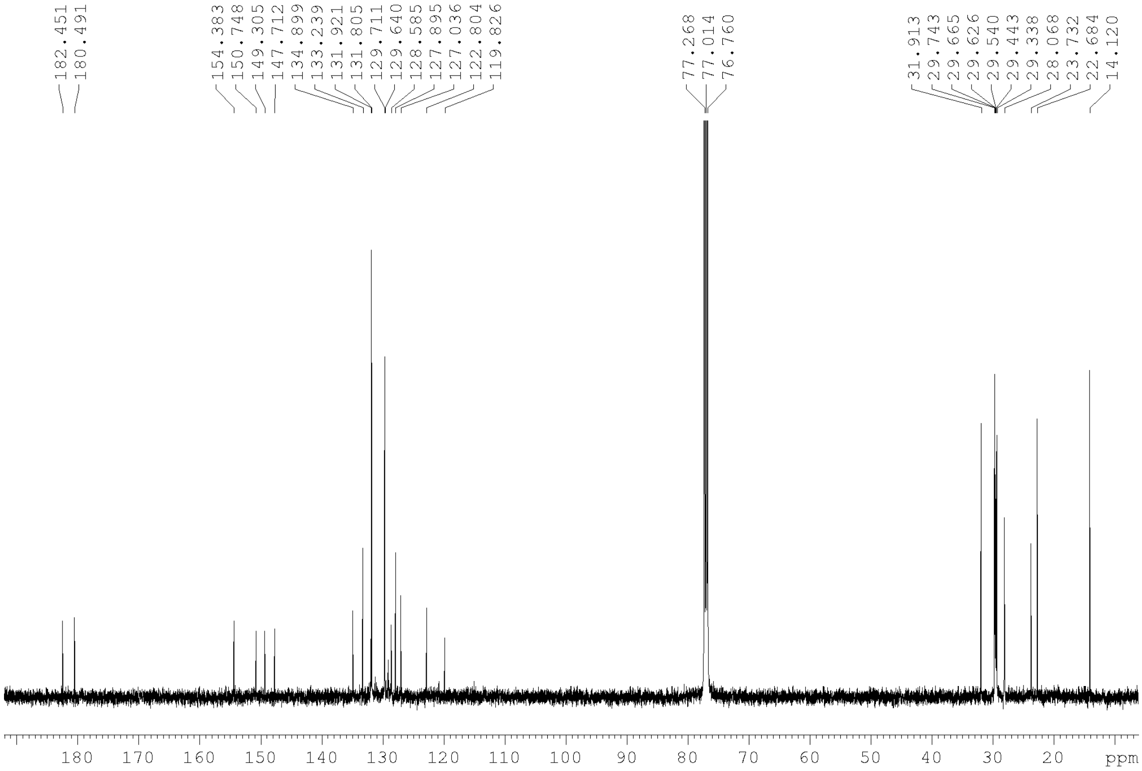

Figure S77. IR spectrum (film) of compound **6d**

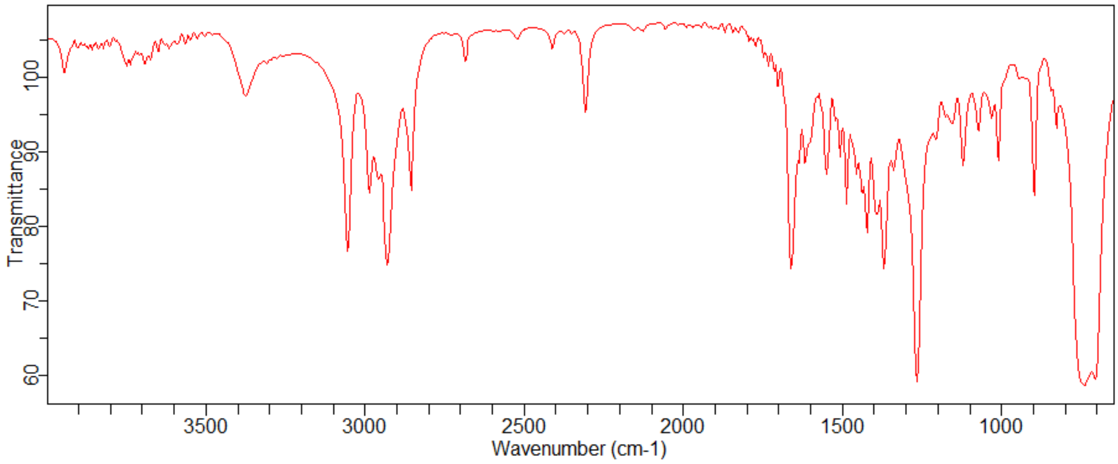

Figure S78. UV spectrum (EtOH) of compound **6d**

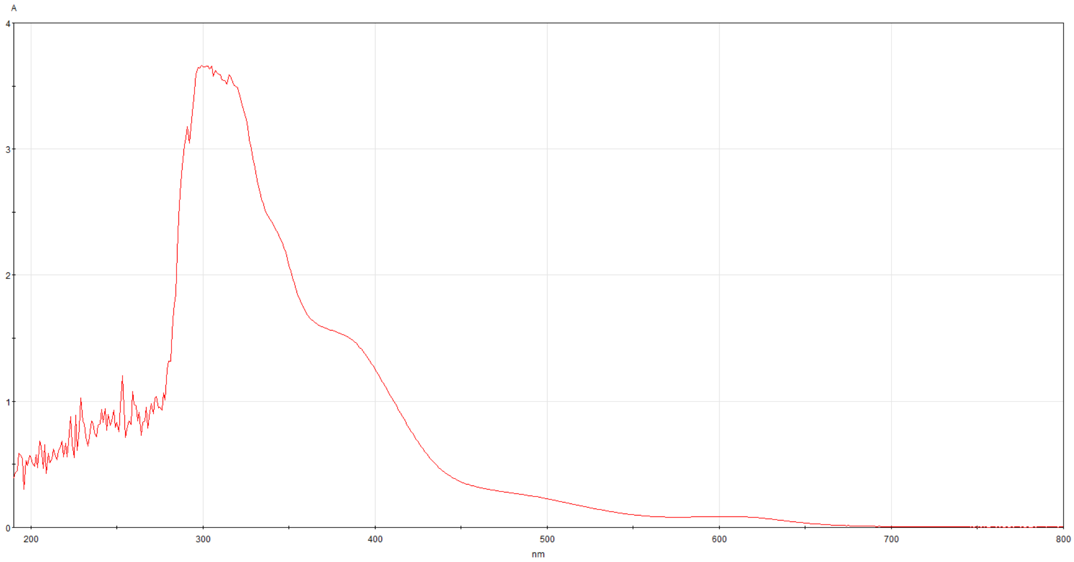

Figure S79. ^{1}H-NMR ($CDCl_3$, 500 MHz) spectrum of compound **6e**

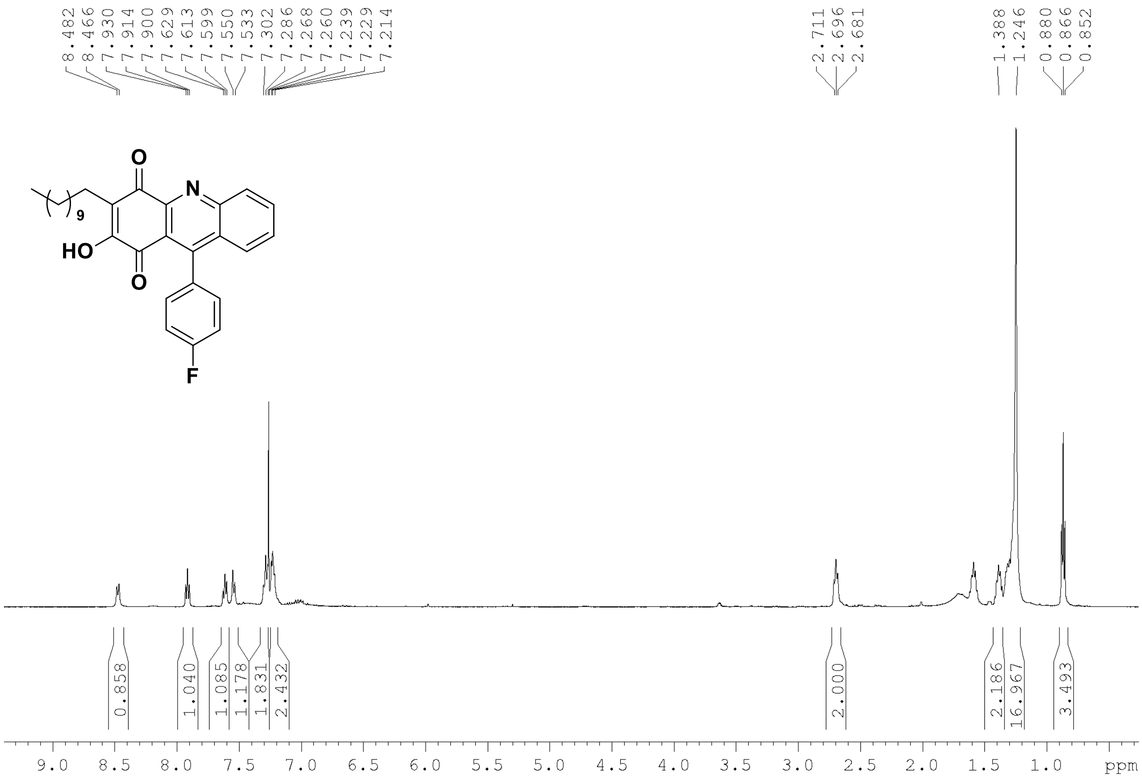

Figure S80. ^{13}C-NMR ($CDCl_3$, 150 MHz) spectrum of compound **6e**

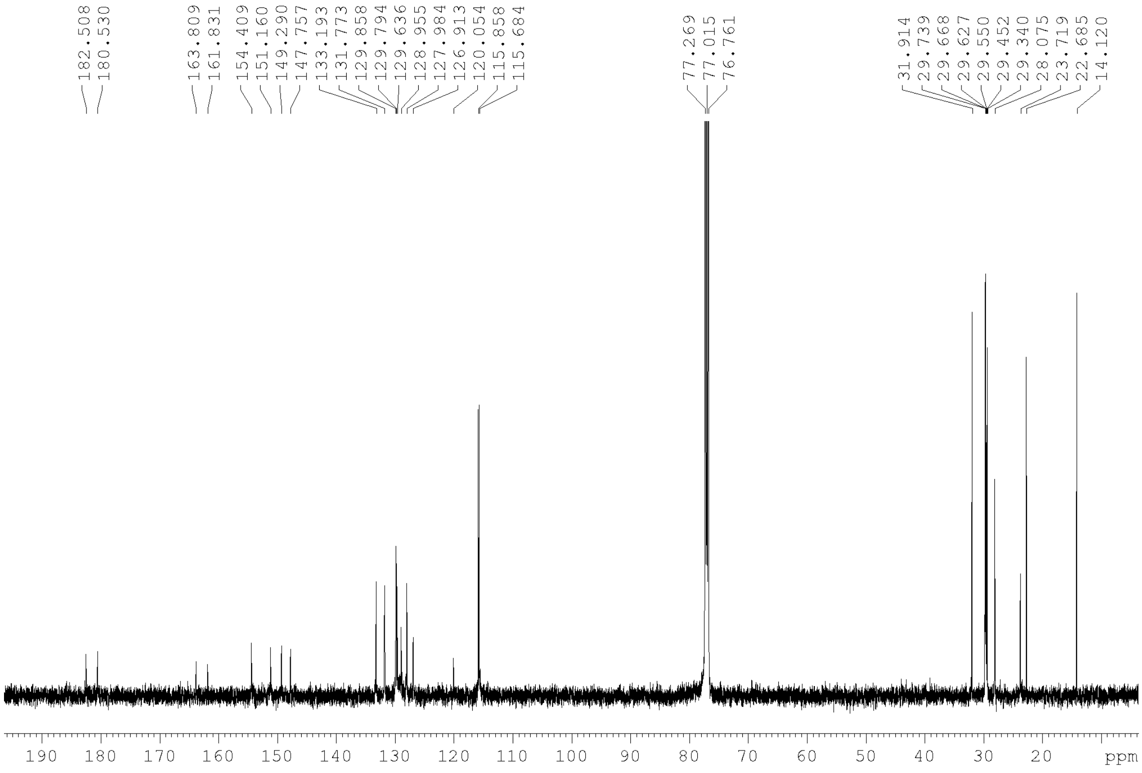

Figure S81. IR spectrum (film) of compound **6e**

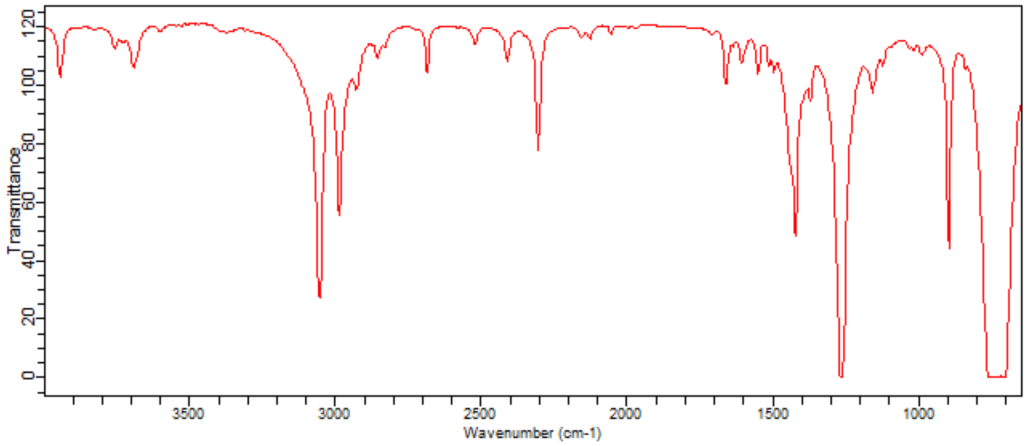

Figure S82. UV spectrum (EtOH) of compound **6e**

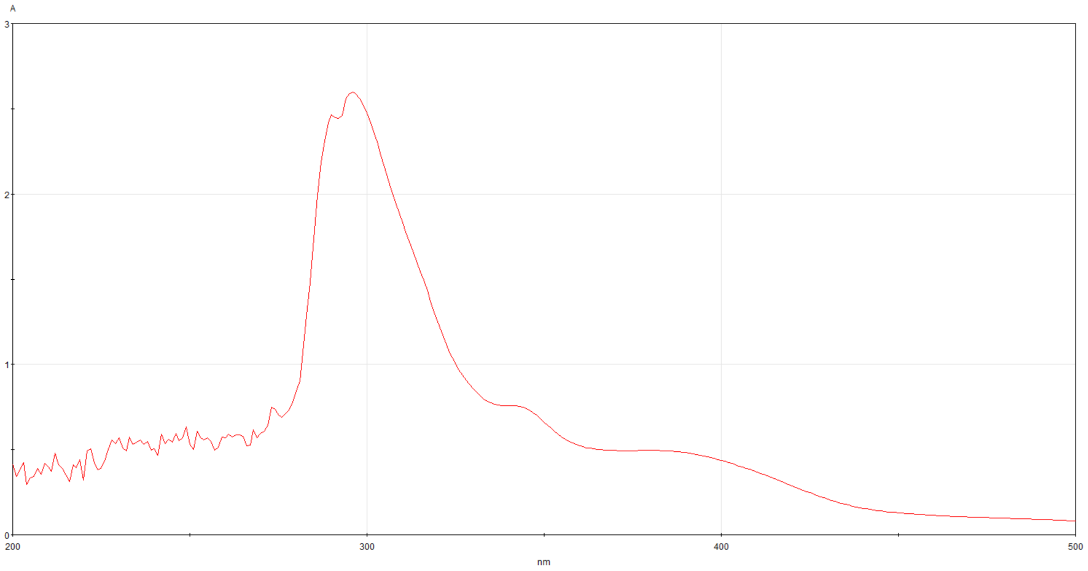

Figure S83. ^{1}H-NMR ($CDCl_3$, 500 MHz) spectrum of compound **6f**

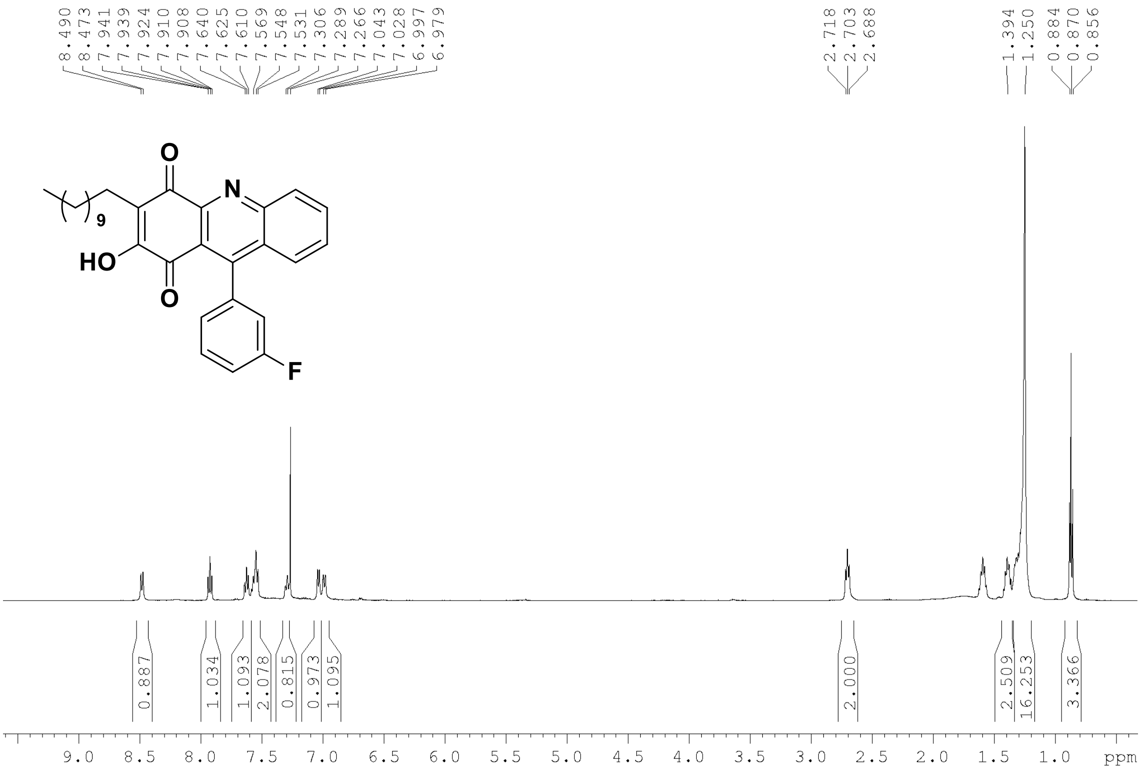

Figure S84. ^{13}C-NMR ($CDCl_3$, 125 MHz) spectrum of compound **6f**

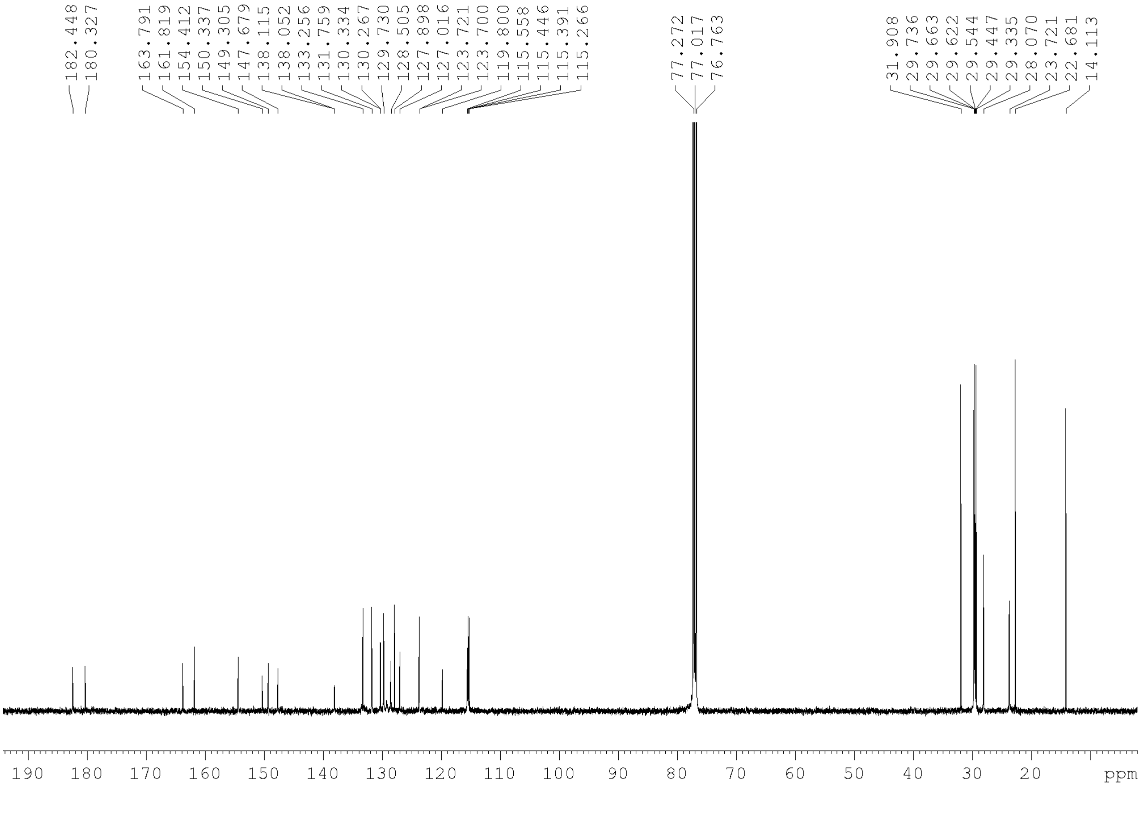

Figure S85. IR spectrum (film) of compound **6f**

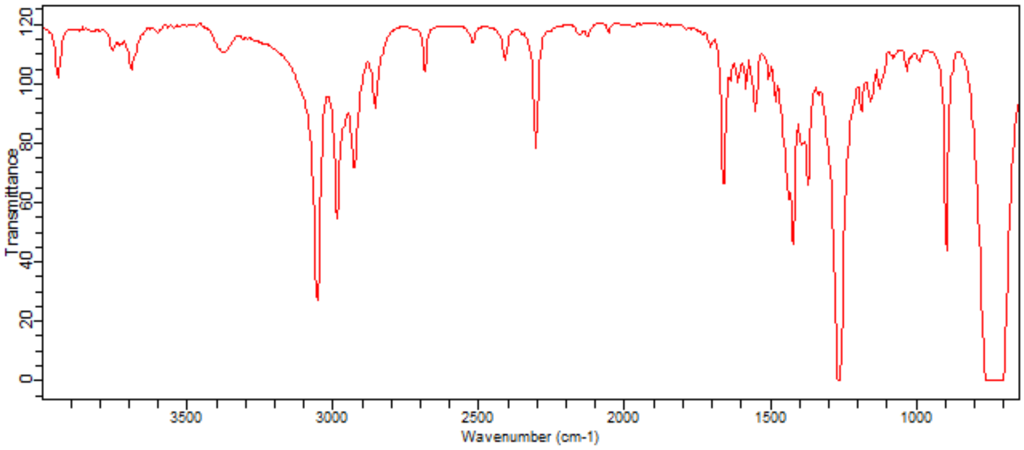

Figure S86. UV spectrum (EtOH) of compound **6f**

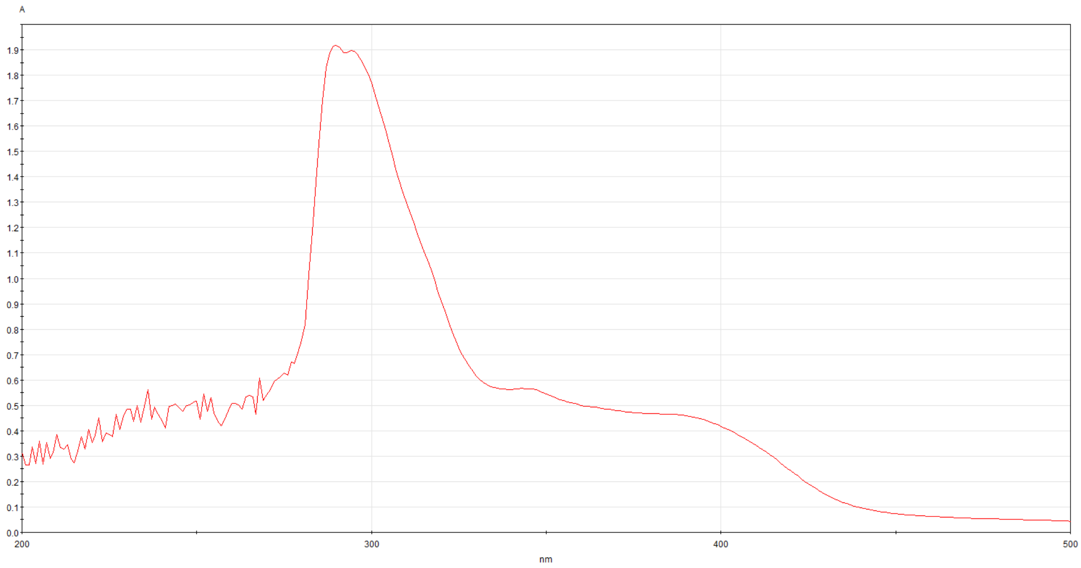

Figure S87. ^{1}H-NMR ($CDCl_3$, 500 MHz) spectrum of compound **6g**

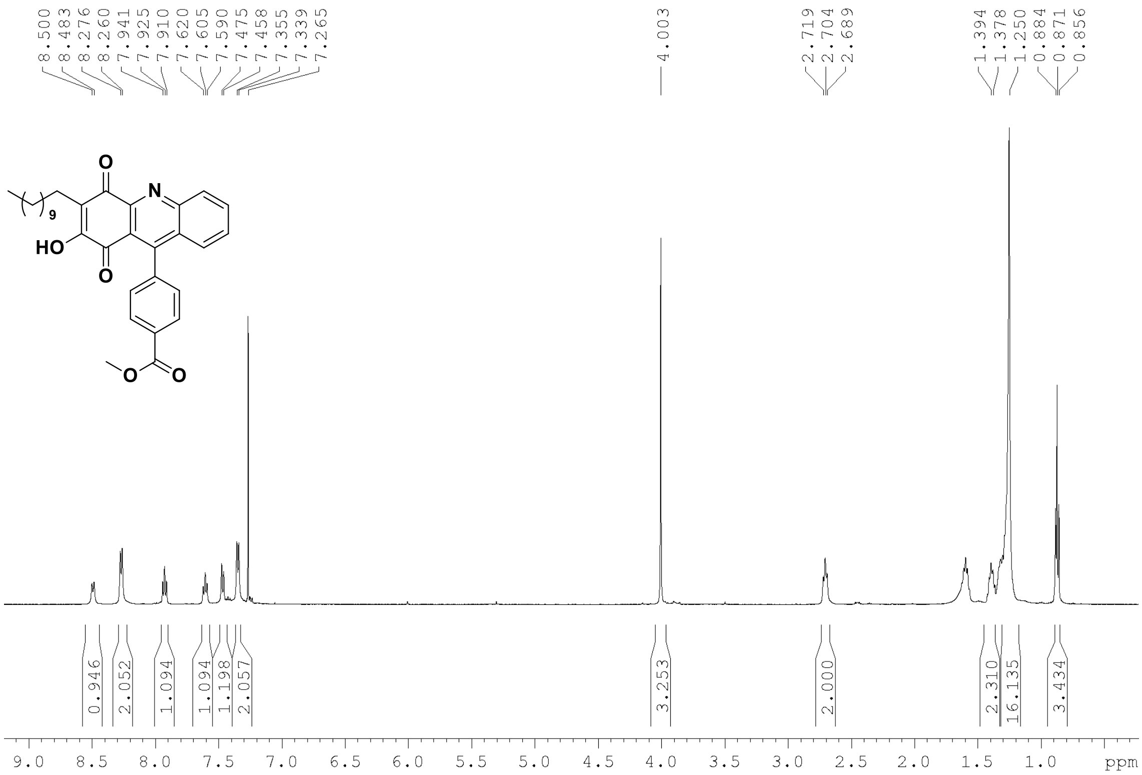

Figure S88. ^{13}C-NMR ($CDCl_3$, 150 MHz) spectrum of compound **6g**

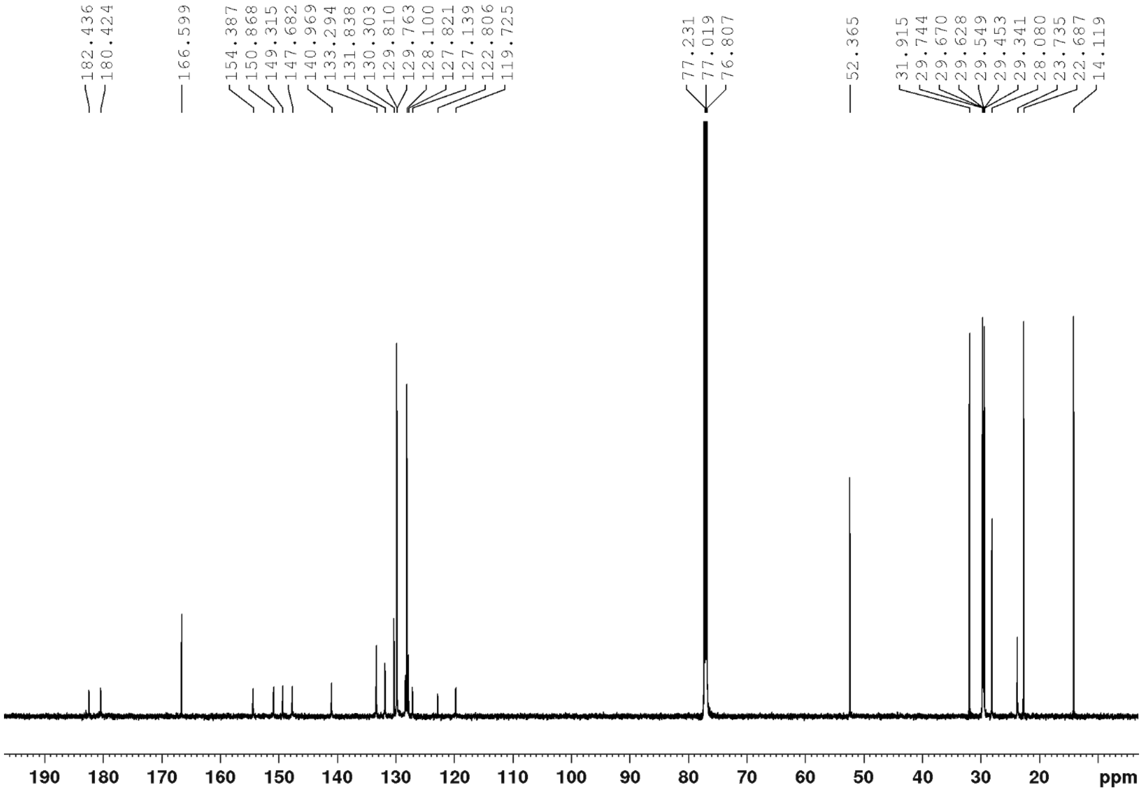

Figure S89. IR spectrum (film) of compound **6g**

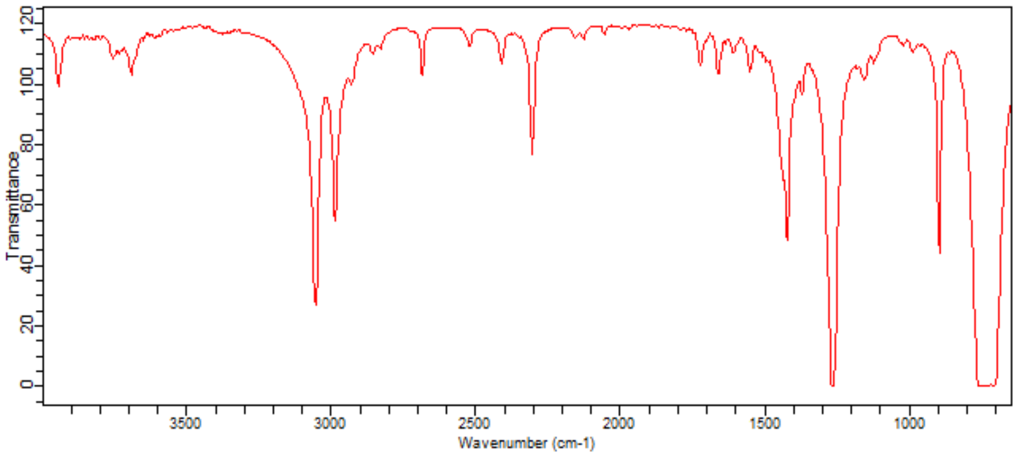

Figure S90. UV spectrum (EtOH) of compound **6g**

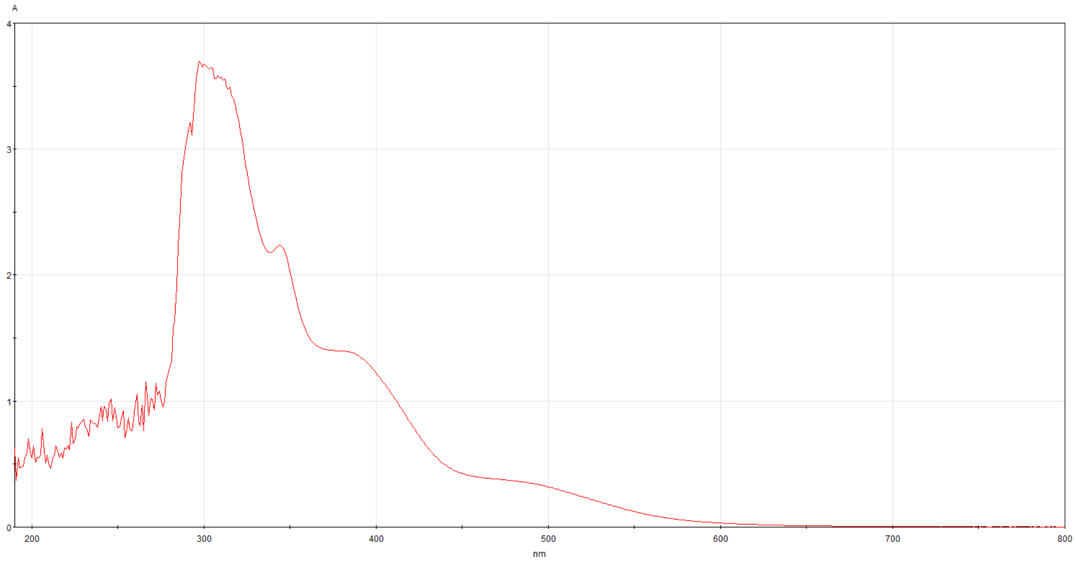

Figure S91. ^{1}H-NMR ($CDCl_3$, 500 MHz) spectrum of compound **6h**

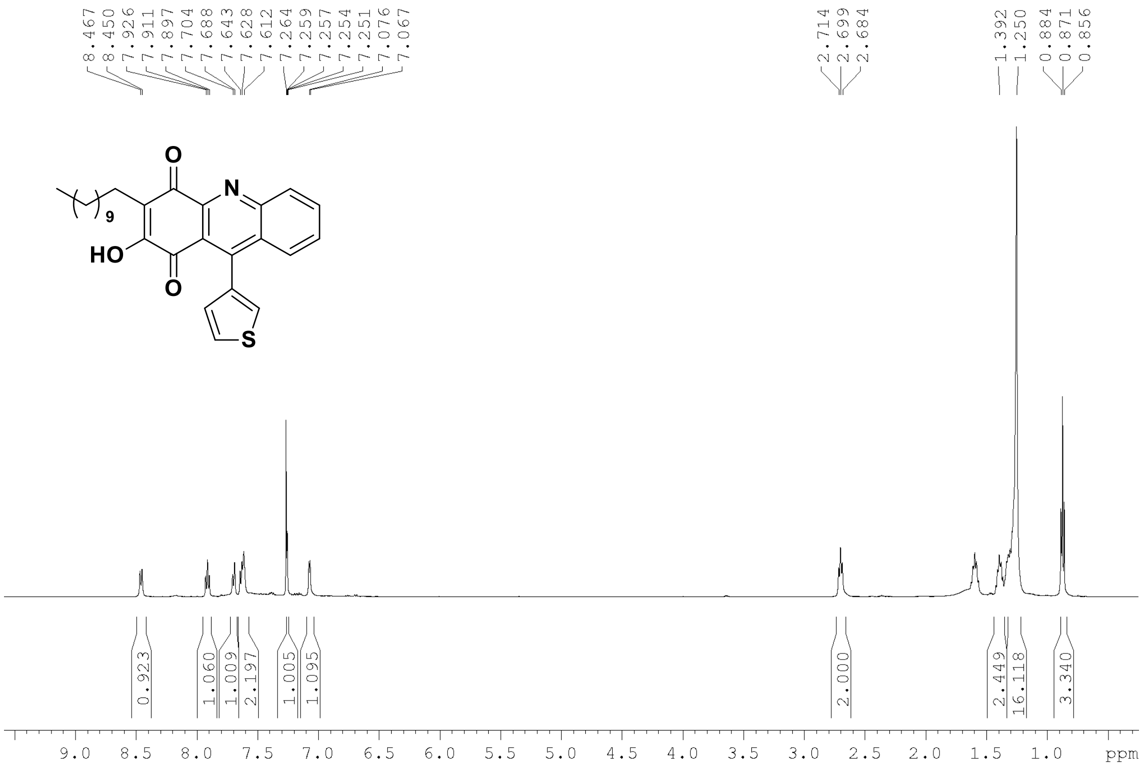

Figure S92. ^{13}C-NMR ($CDCl_3$, 125 MHz) spectrum of compound **6h**

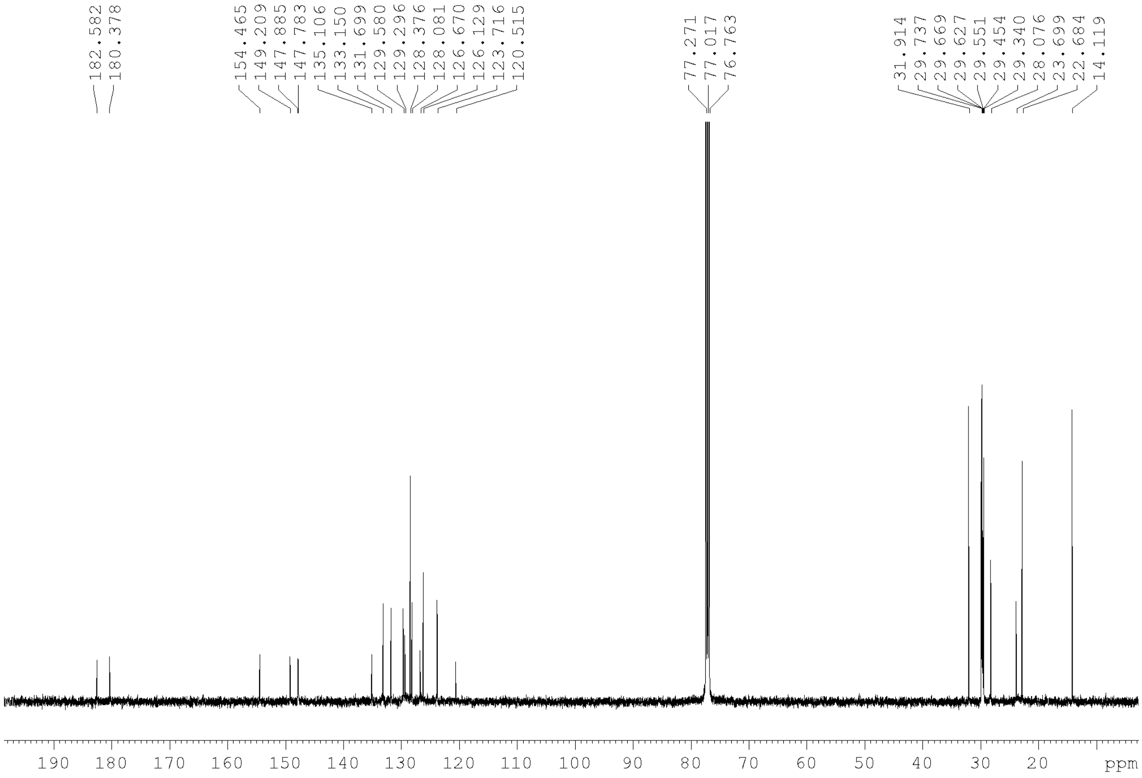

Figure S93. IR spectrum (film) of compound **6h**

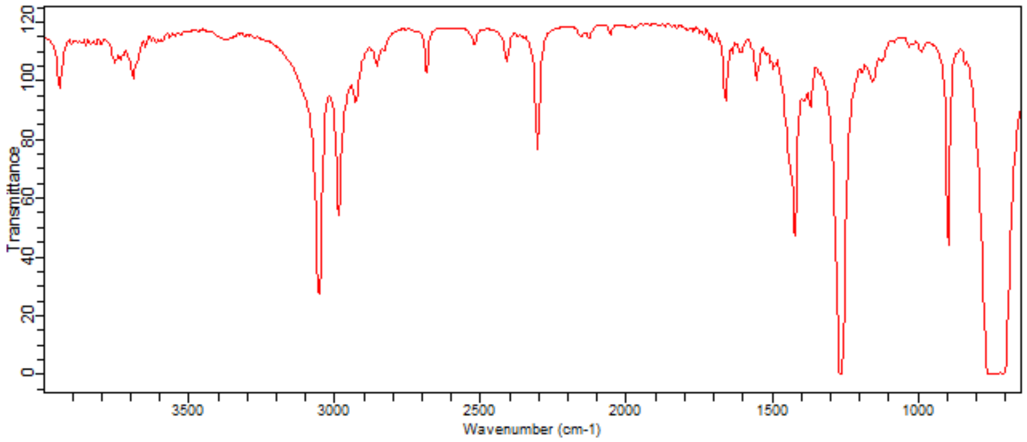

Figure S94. UV spectrum (EtOH) of compound **6h**

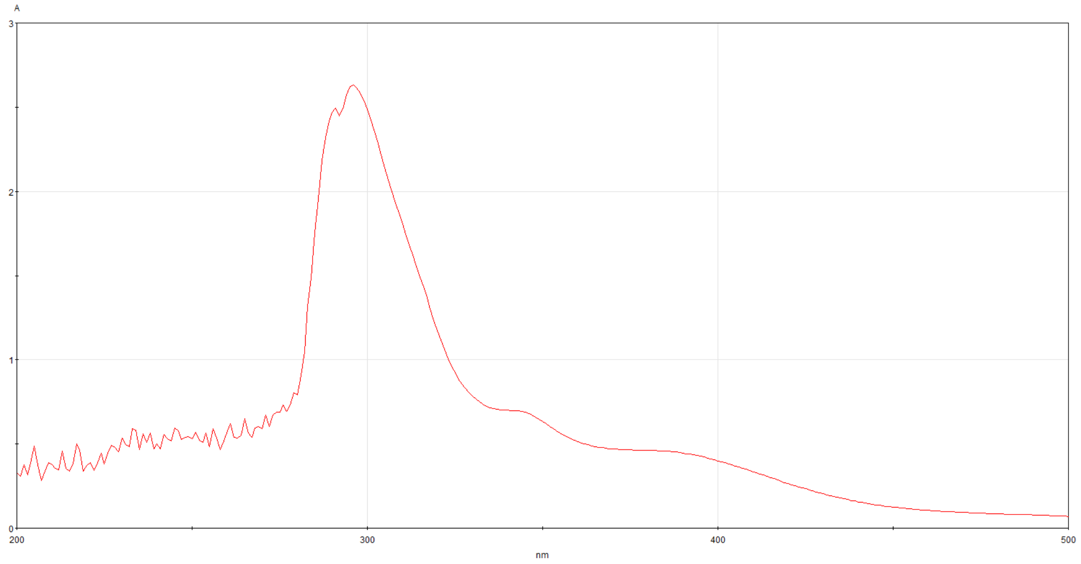

Figure S95. ^{1}H-NMR ($CDCl_3$, 500 MHz) spectrum of compound **6i**

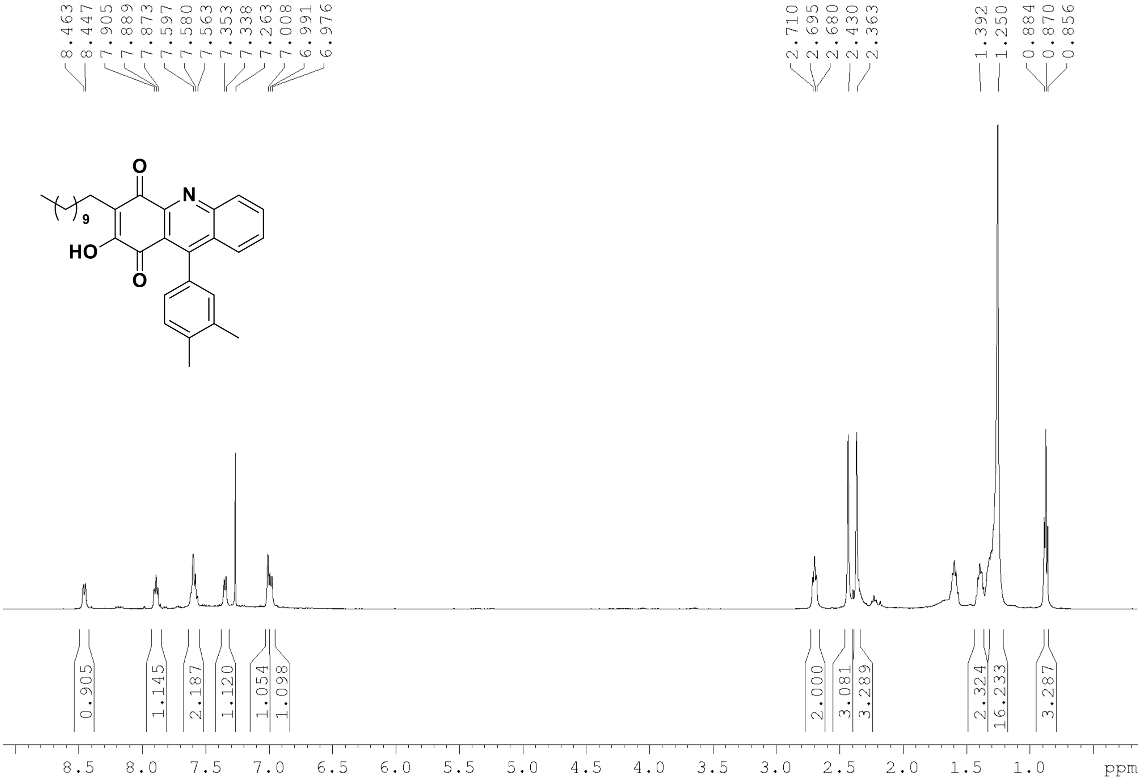

Figure S96. ^{13}C-NMR ($CDCl_3$, 125 MHz) spectrum of compound **6i**

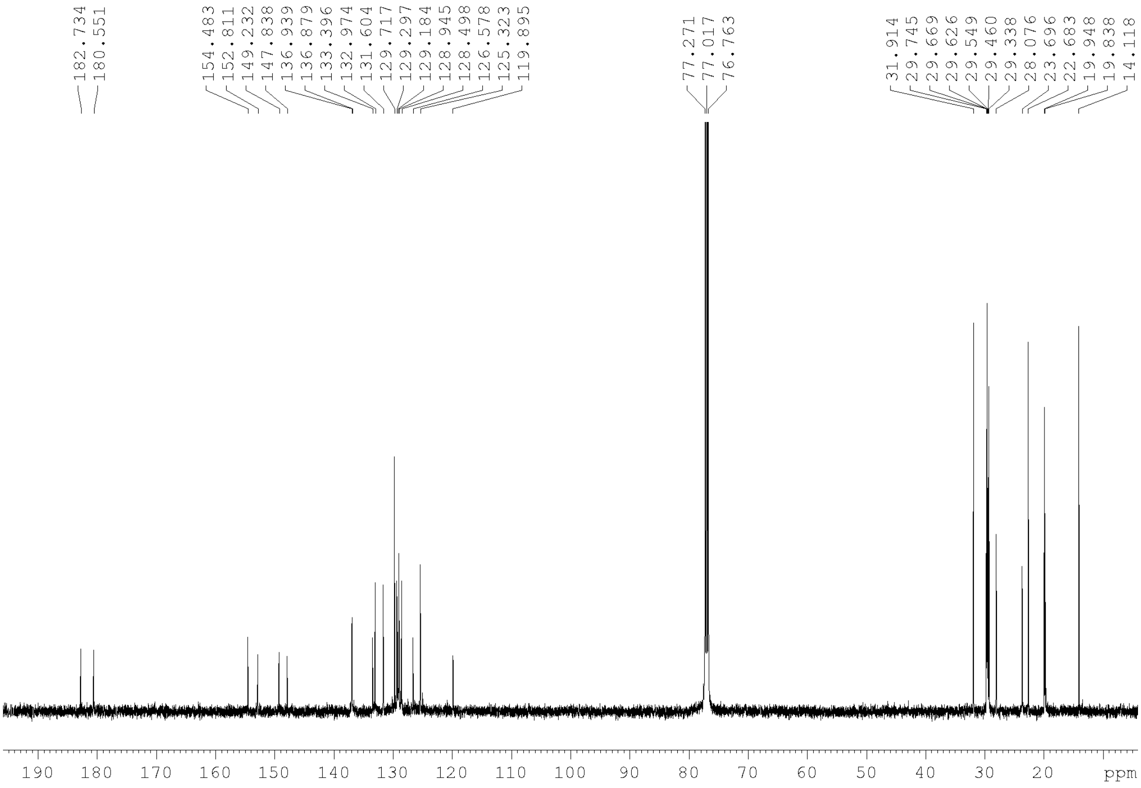

Figure S97. IR spectrum (film) of compound **6i**

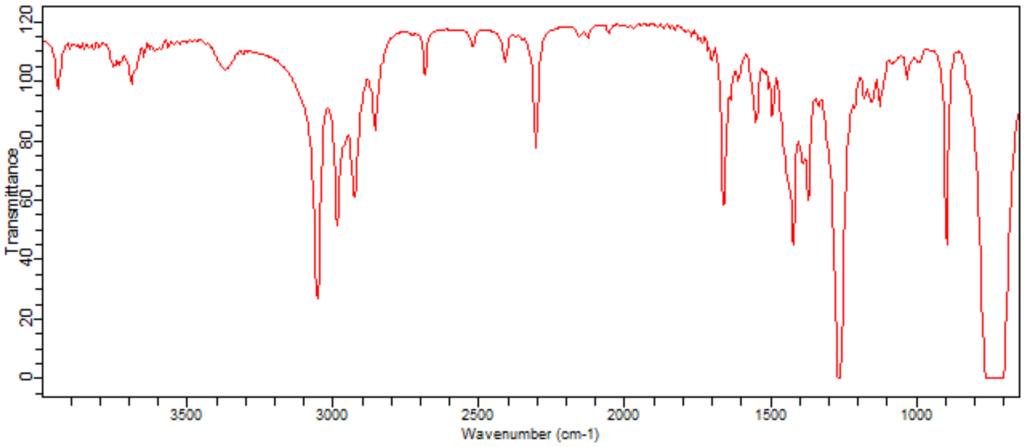

Figure S98. UV spectrum (EtOH) of compound **6i**

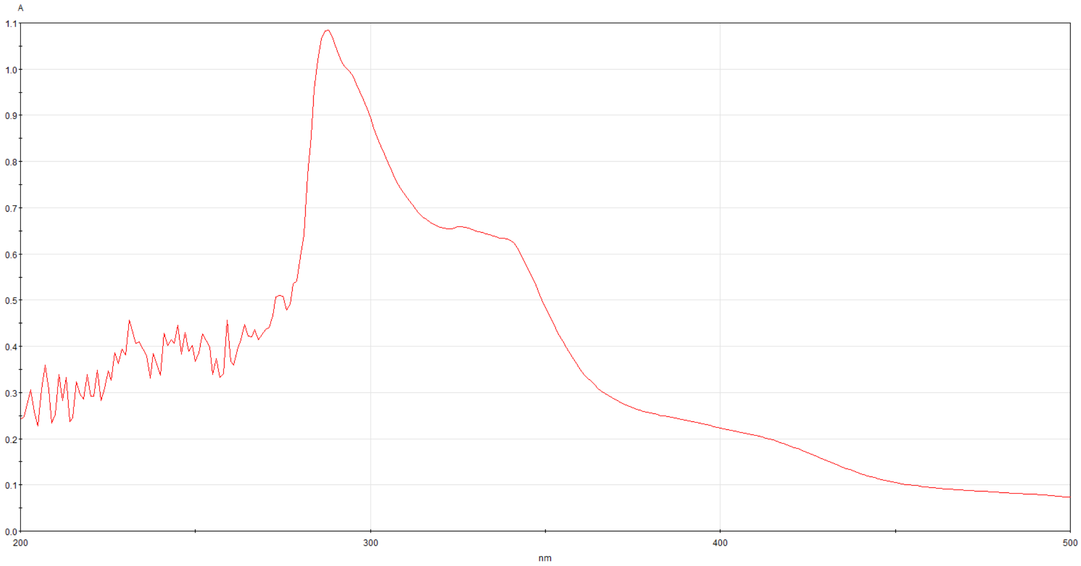

Figure S99. ^{1}H-NMR ($CDCl_3$, 500 MHz) spectrum of compound **6j**

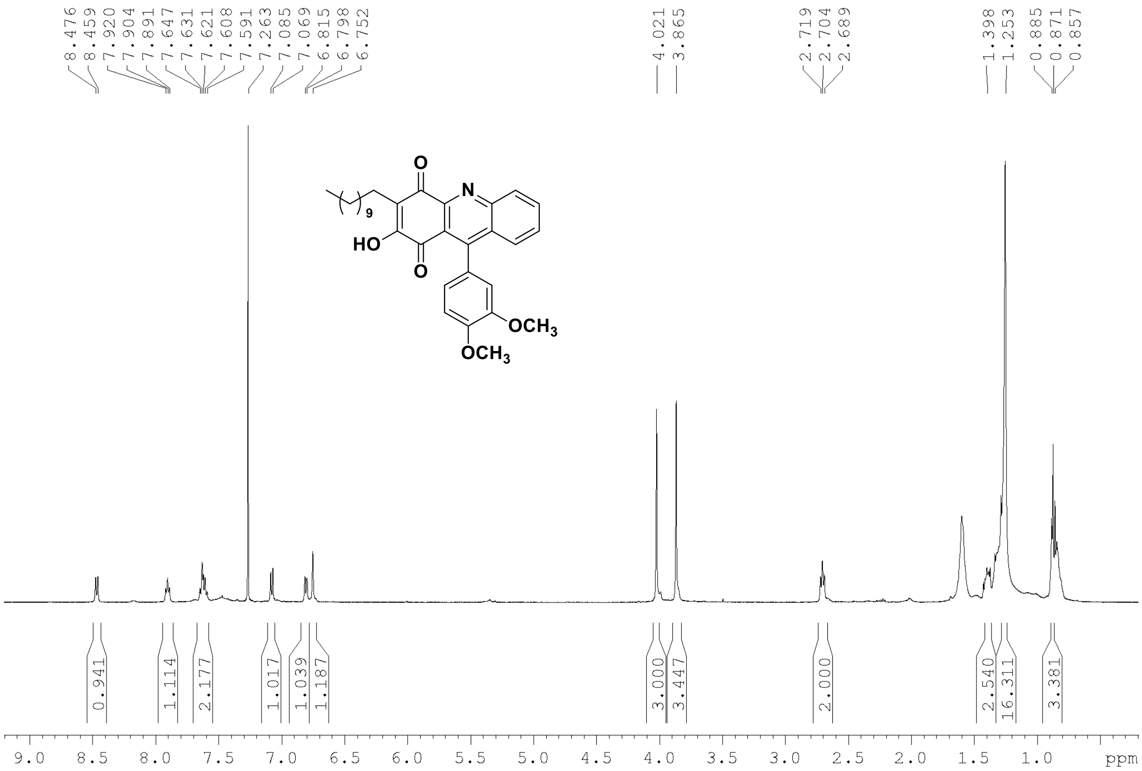

Figure S100. ^{13}C-NMR ($CDCl_3$, 125 MHz) spectrum of compound **6j**

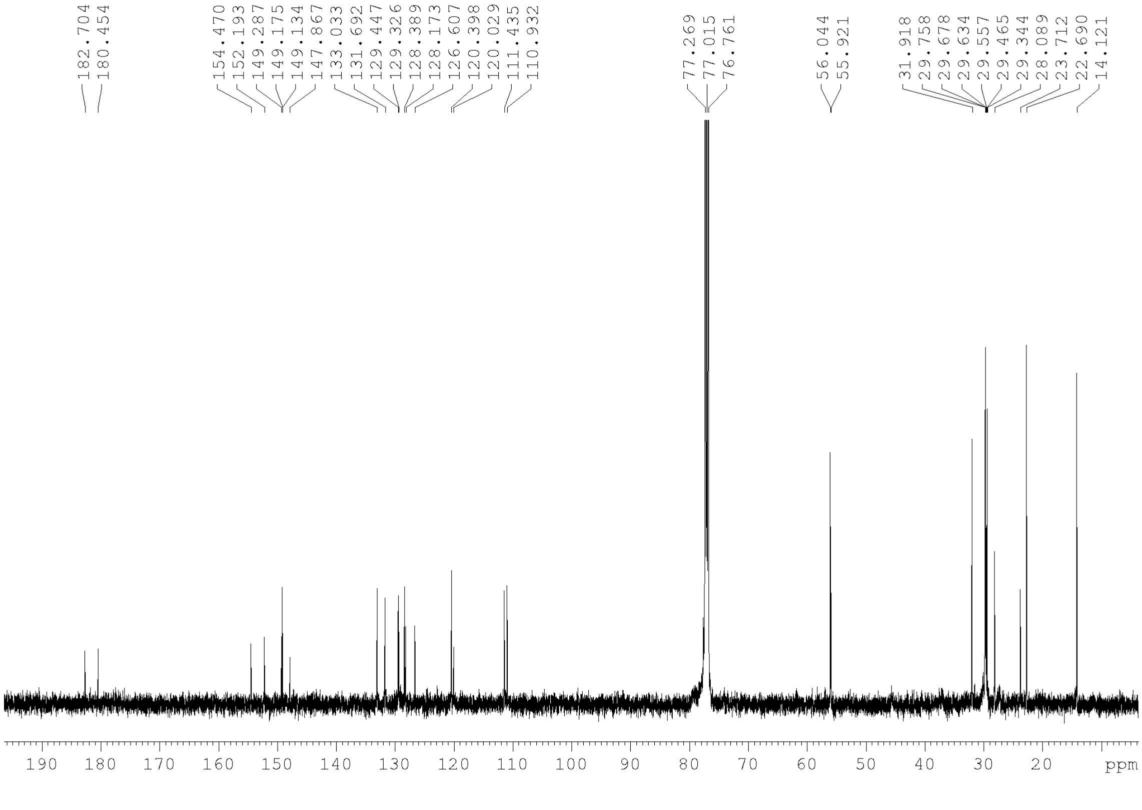

Figure S101. IR spectrum (film) of compound **6j**

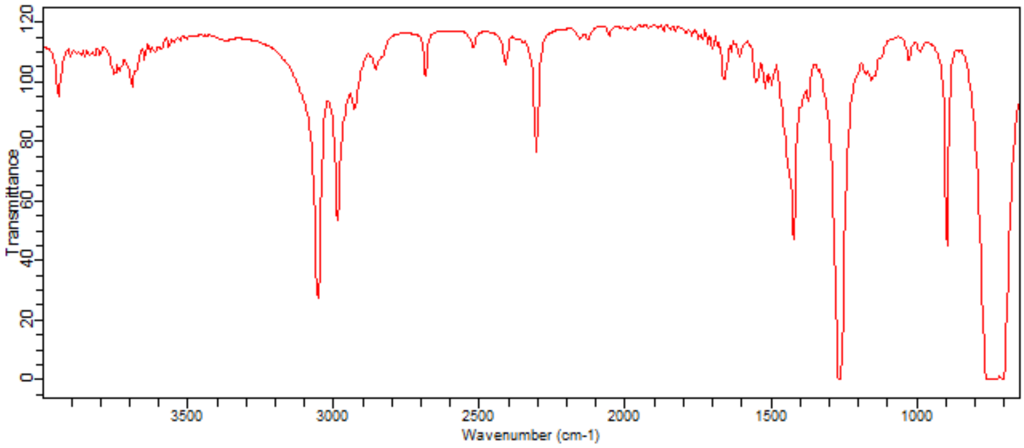

Figure S102. UV spectrum (EtOH) of compound **6j**

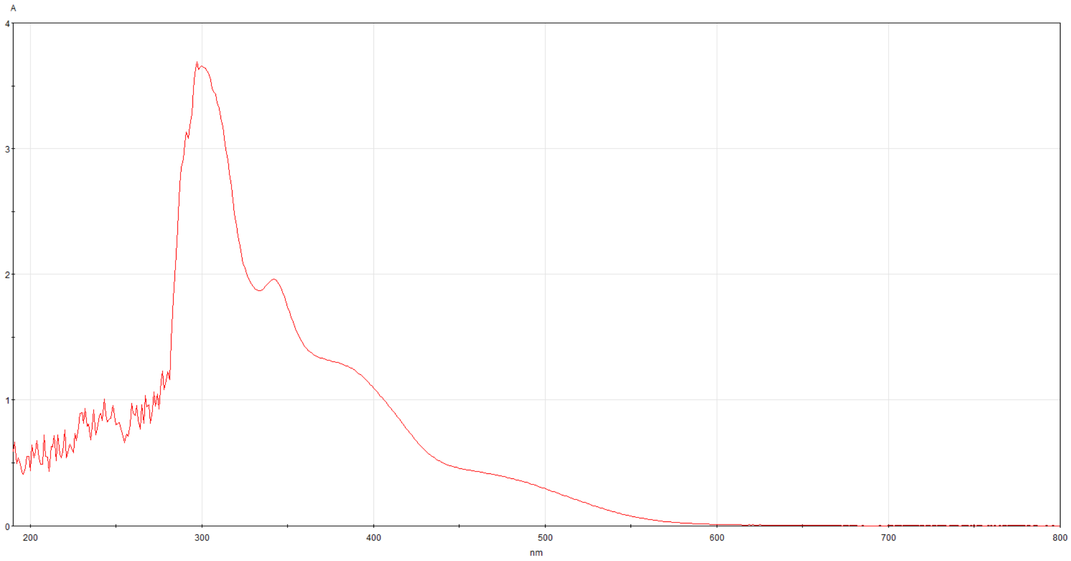

Figure S103. ^{1}H-NMR ($CDCl_3$, 500 MHz) spectrum of compound **6k**

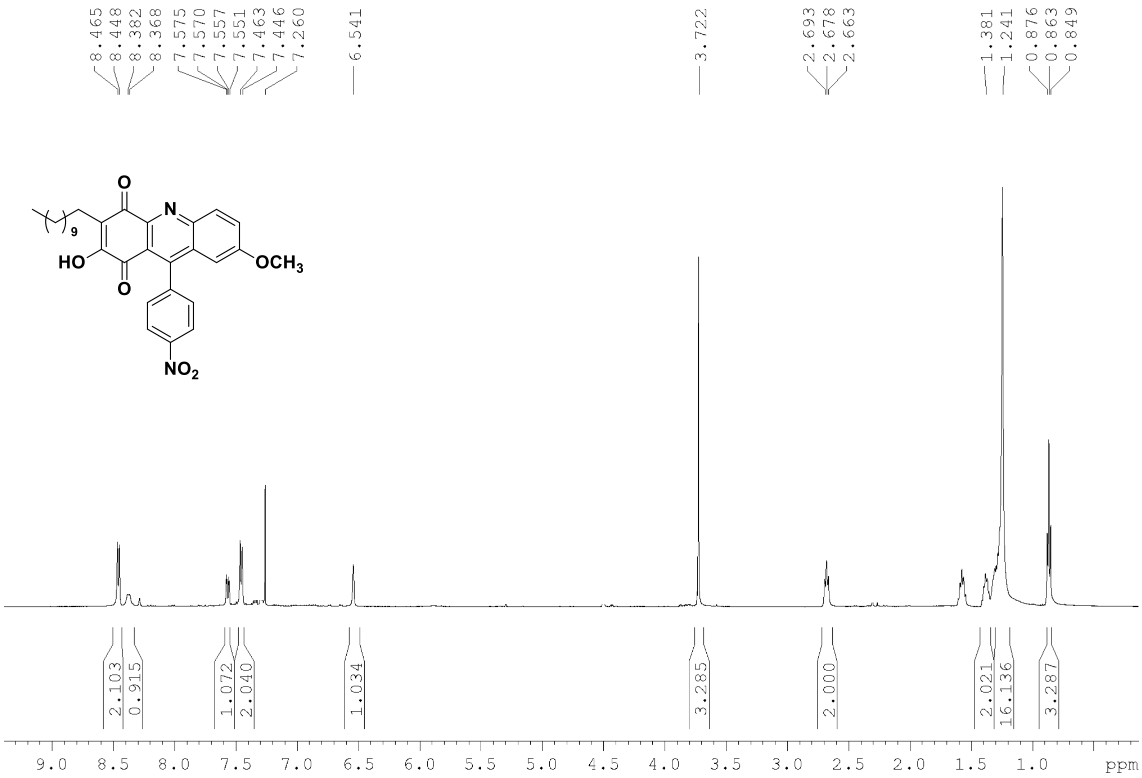

Figure S104. ^{13}C-NMR ($CDCl_3$, 125 MHz) spectrum of compound **6k**

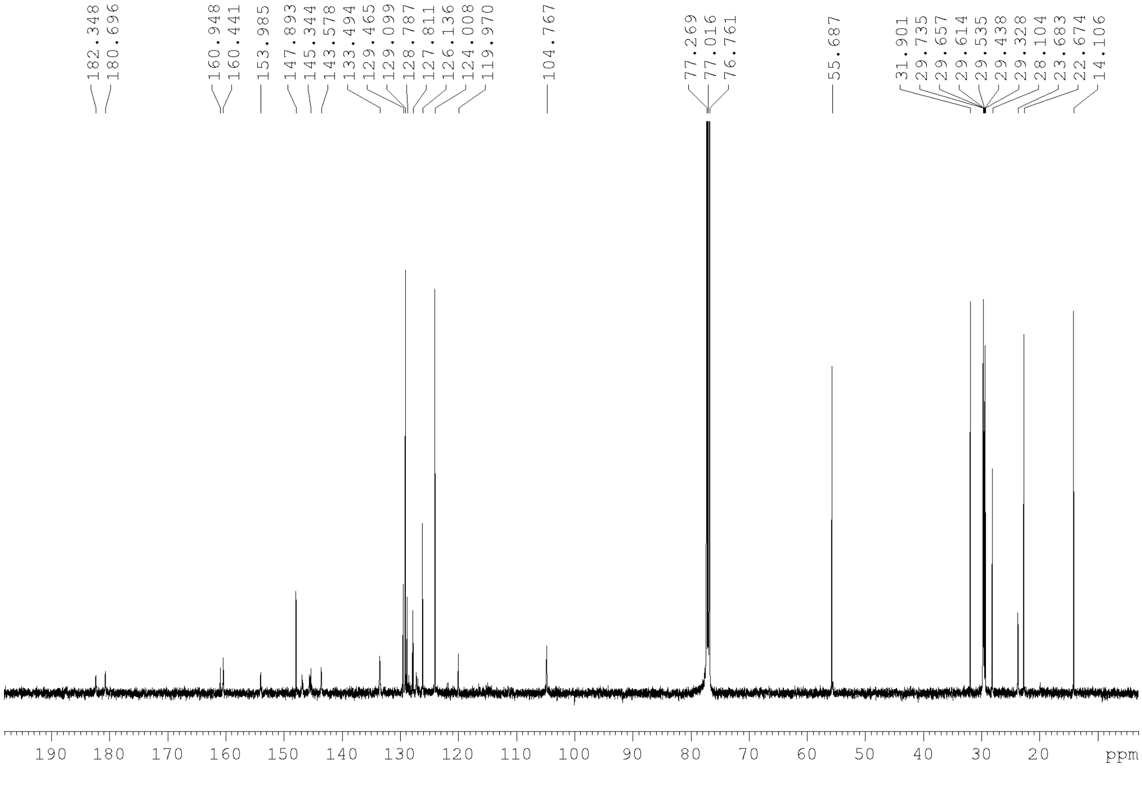

Figure S105. IR spectrum (film) of compound **6k**

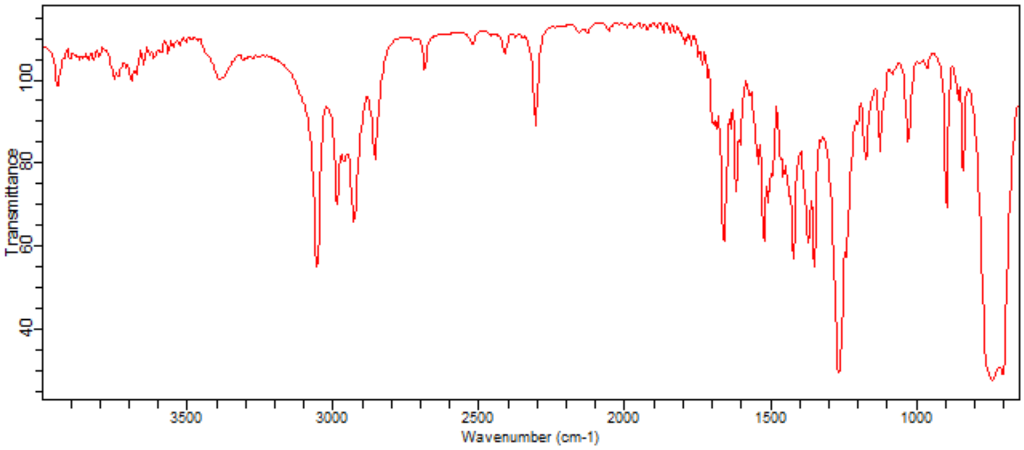

Figure S106. UV spectrum (EtOH) of compound **6k**

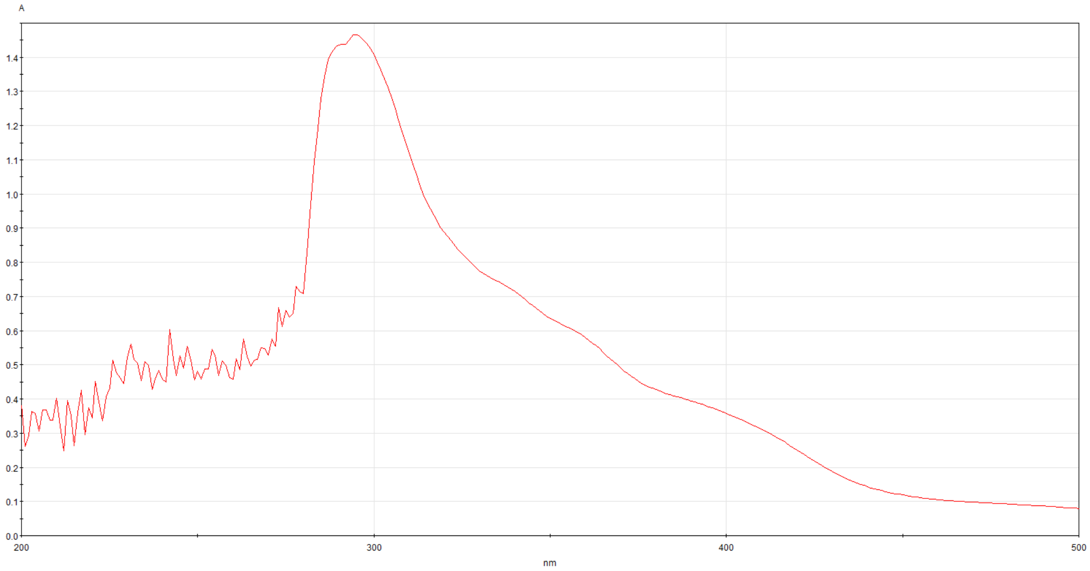

Figure S107. ^{1}H-NMR ($CDCl_3$, 500 MHz) spectrum of compound **6l**

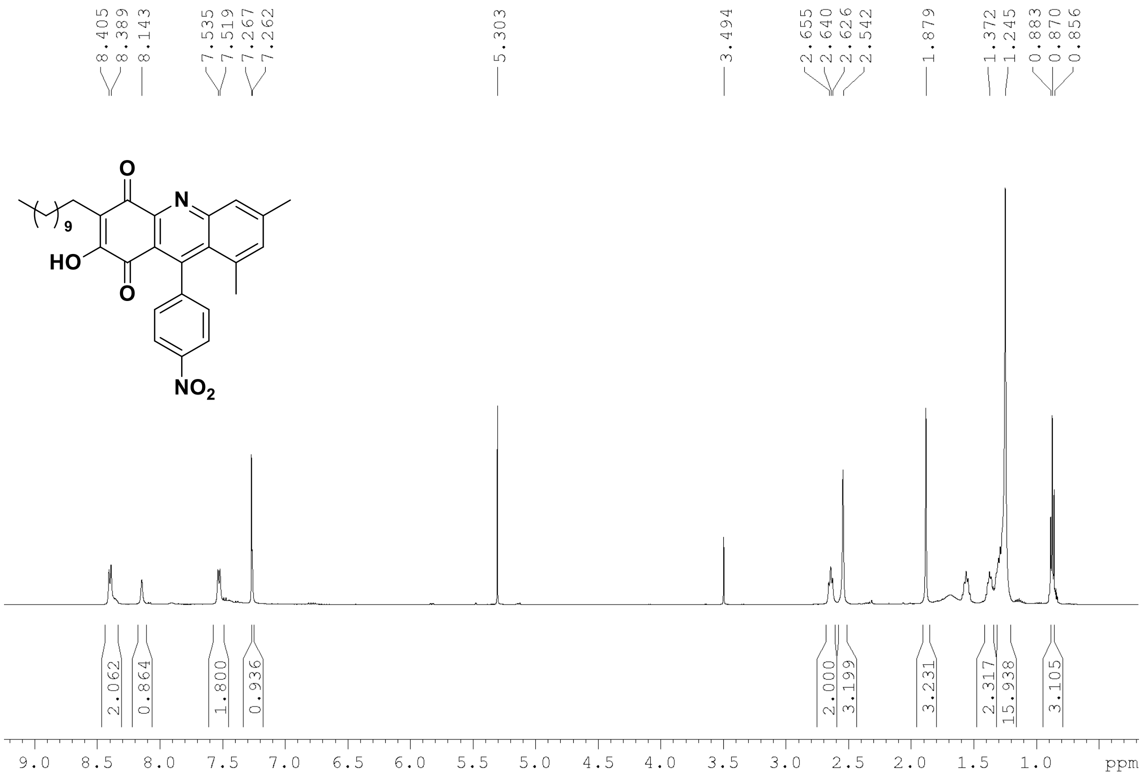

Figure S108. ^{13}C-NMR ($CDCl_3$, 125 MHz) spectrum of compound **6l**

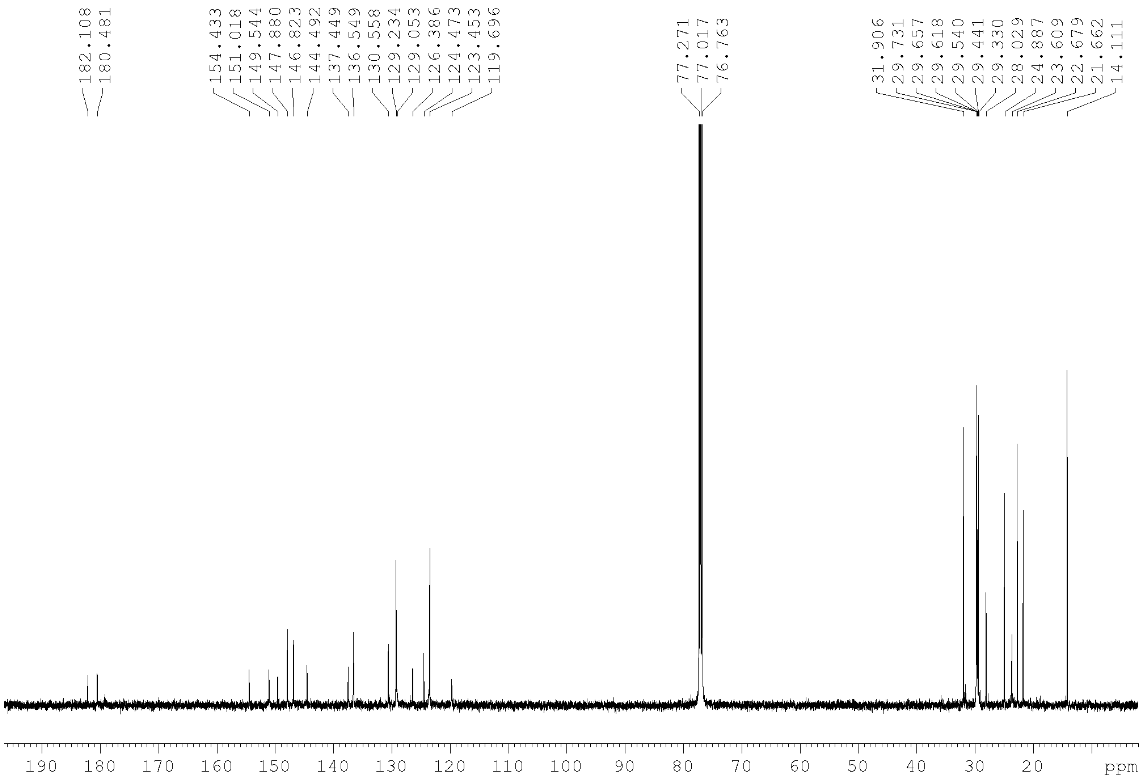

Figure S109. IR spectrum (film) of compound **6l**

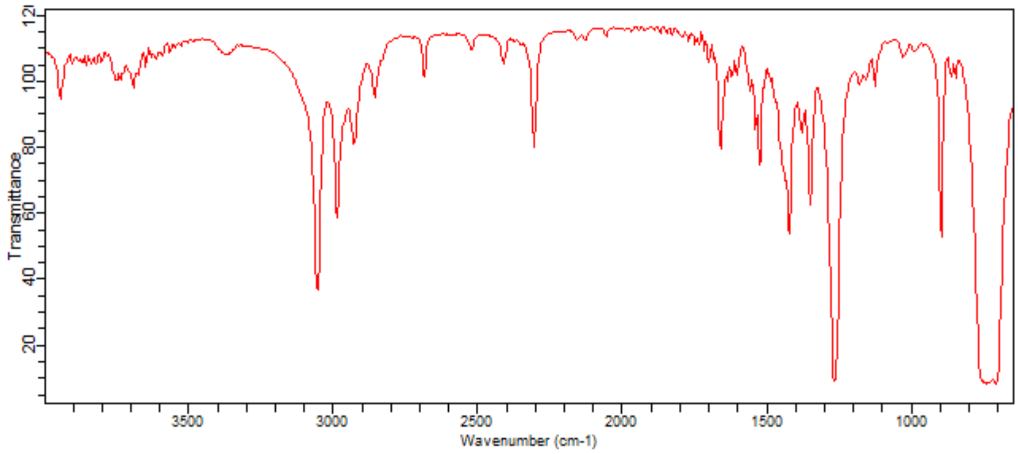

Figure S110. UV spectrum (EtOH) of compound **6l**

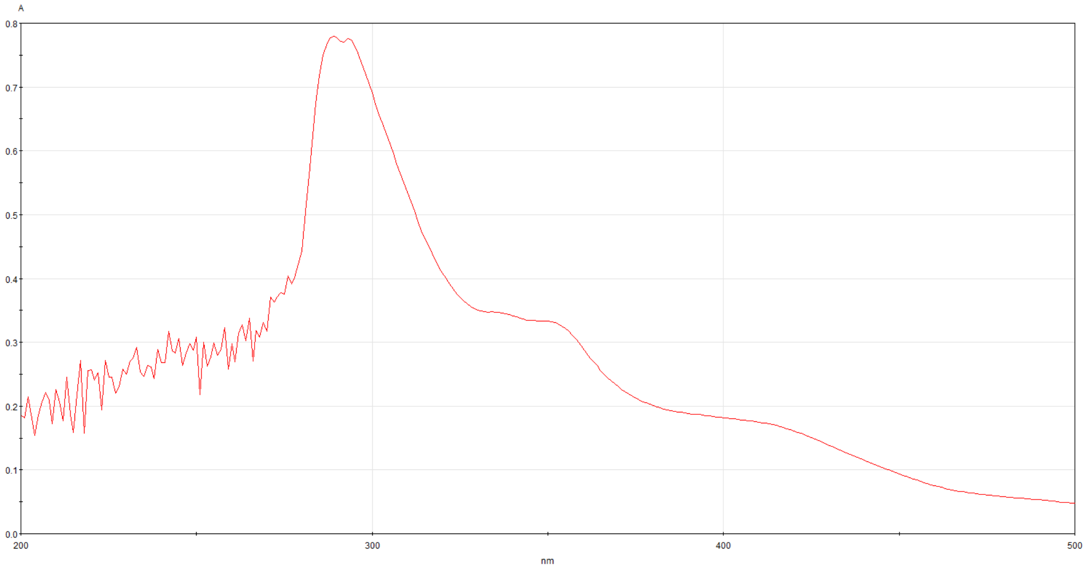

Figure S111. ^{1}H-NMR ($CDCl_3$, 500 MHz) spectrum of compound **6m**

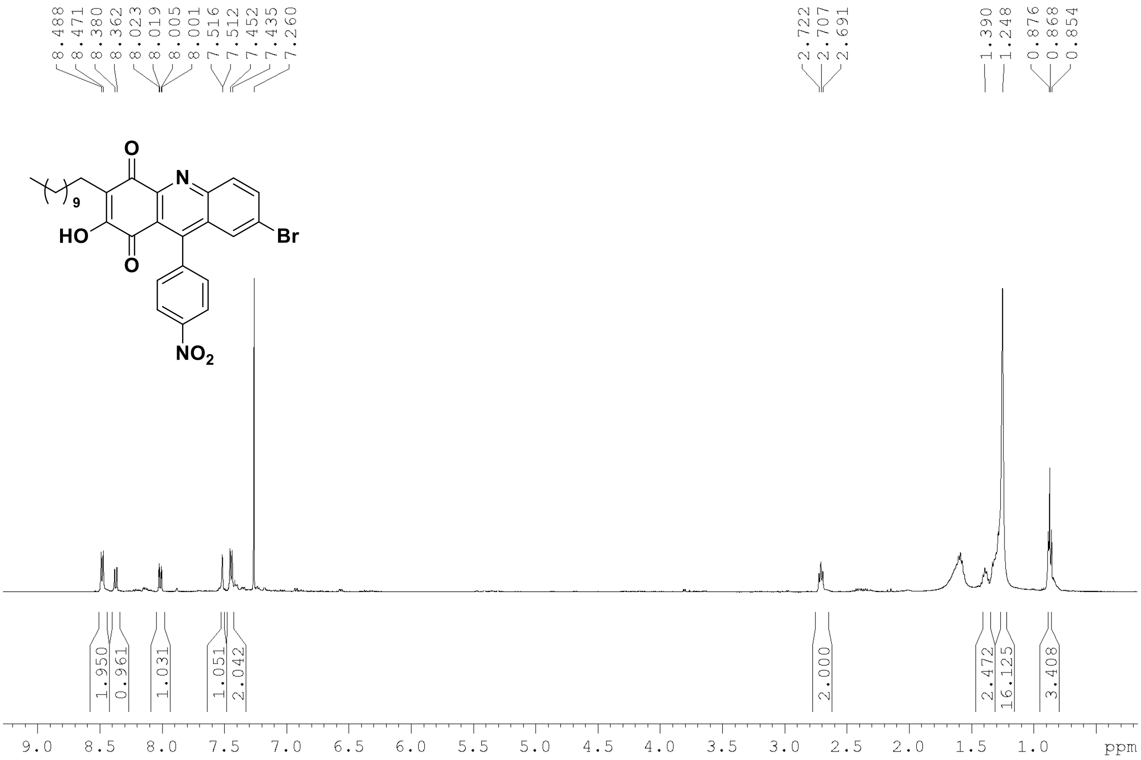

Figure S112. ^{13}C-NMR ($CDCl_3$, 125 MHz) spectrum of compound **6m**

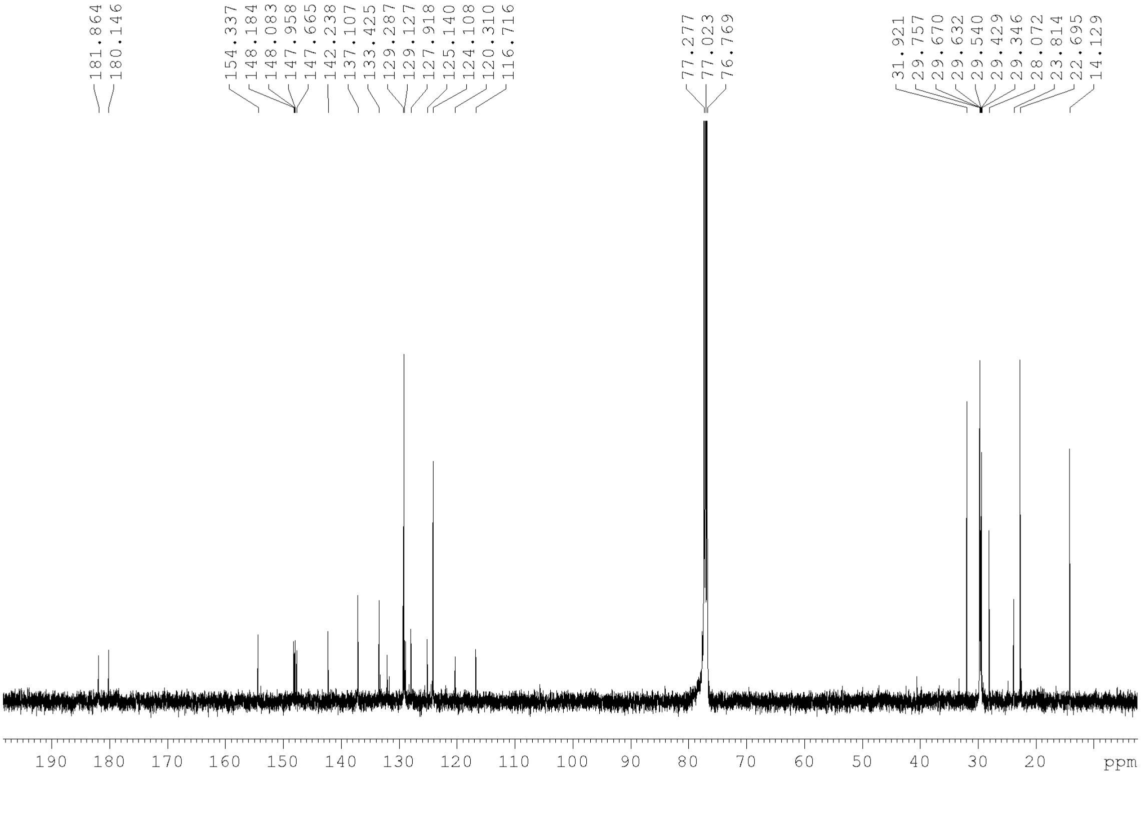

Figure S113. IR spectrum (film) of compound **6m**

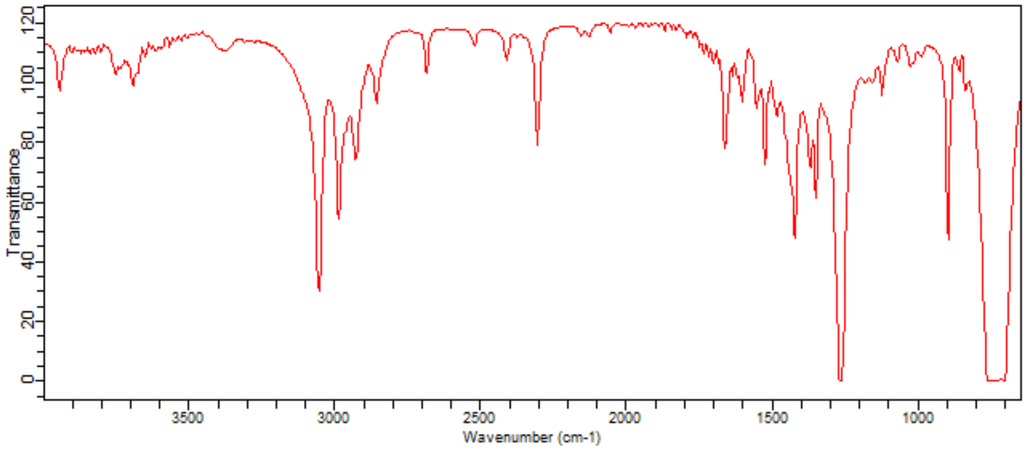

Figure S114. UV spectrum (EtOH) of compound **6m**

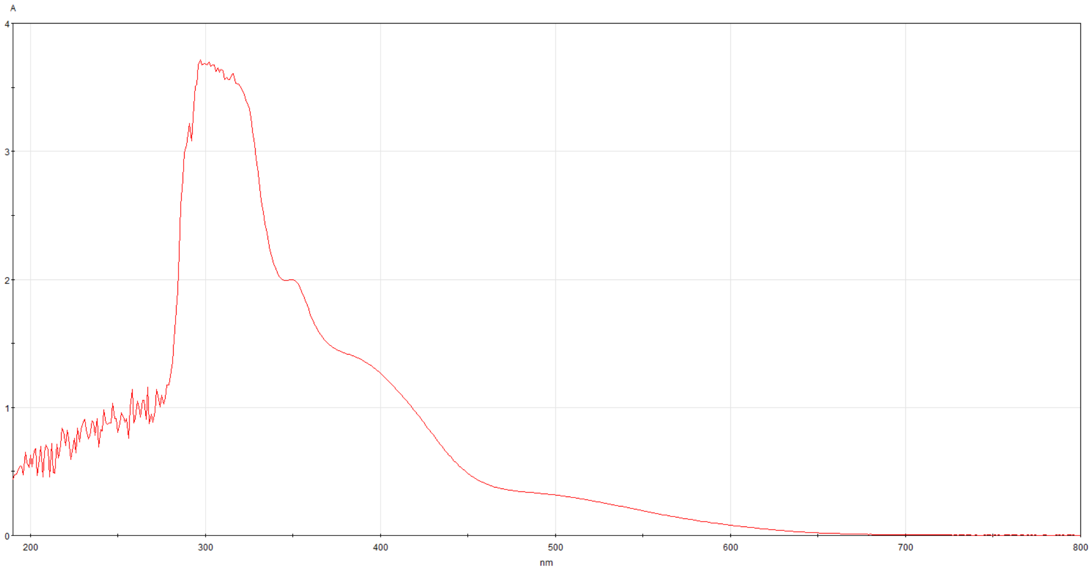

Supplement: Supplementary file 1 — np2c00924_si_001.pdf [file np2c00924_si_001.pdf]
